# Imidazoquinoline Derivatives as Potential Inhibitors of InhA Enzyme and *Mycobacterium tuberculosis*

**DOI:** 10.3390/molecules29133076

**Published:** 2024-06-27

**Authors:** Pascal Hoffmann, Joëlle Azéma-Despeyroux, Fernanda Goncalves, Alessandro Stamilla, Nathalie Saffon-Merceron, Frédéric Rodriguez, Giulia Degiacomi, Maria Rosalia Pasca, Christian Lherbet

**Affiliations:** 1 Laboratoire de Synthèse et Physico-Chimie de Molécules d’Intérêt Biologique (SPCMIB), UMR5068, CNRS, Université Paul Sabatier Toulouse III, 31062 Toulouse, France; pascal.hoffmann@univ-tlse3.fr (P.H.); joelle.azema-despeyroux@univ-tlse3.fr (J.A.-D.); fernanda.goncalves@cnrs.fr (F.G.); frederic.rodriguez@univ-tlse3.fr (F.R.); 2 Department of Biology and Biotechnology “Lazzaro Spallanzani”, University of Pavia, 27100 Pavia, Italy; alessandro.stamilla@unipv.it (A.S.); giulia.degiacomi@unipv.it (G.D.); mariarosalia.pasca@unipv.it (M.R.P.); 3 Institut de Chimie de Toulouse, ICT-UAR2599, Université Paul Sabatier Toulouse III, 31062 Toulouse, France; nathalie.saffon@univ-tlse3.fr

**Keywords:** mycobacterium tuberculosis, InhA enzyme, inhibitor, imidazoquinoline, triazolophthalazine

## Abstract

Tuberculosis is a serious public health problem worldwide. The search for new antibiotics has become a priority, especially with the emergence of resistant strains. A new family of imidazoquinoline derivatives, structurally analogous to triazolophthalazines, which had previously shown good antituberculosis activity, were designed to inhibit InhA, an essential enzyme for *Mycobacterium tuberculosis* survival. Over twenty molecules were synthesized and the results showed modest inhibitory efficacy against the protein. Docking experiments were carried out to show how these molecules could interact with the protein’s substrate binding site. Disappointingly, unlike triazolophthlazines, these imidazoquinoline derivatives showed an absence of inhibition on mycobacterial growth.

## 1. Introduction

Tuberculosis (TB) is a worldwide scourge, with one third of the total world population infected by Mycobacterium tuberculosis (MTB). According to a WHO report, 10 million people developed TB disease in 2022 and a total of 1.3 million died from TB [1]. Despite significant and long-term efforts, tuberculosis represents an ongoing public health threat. Drug resistance could be observed and especially multidrug resistance (MDR). MDR tuberculosis is a form caused by mycobacteria that did not respond to the most used first-line drugs, i.e., isoniazid (INH) and rifampicin. The discovery of new drugs has become a necessity and represents a challenge for the scientific community. Fortunately, new drugs have appeared on the market in recent years, such as bedaquiline [2], nitroimidazoles pretomanid [3], and delamanid [4], for which resistances are already emerging. Other promising drugs such as PBTZ169 and TBA7371, potent inhibitors of decaprenylphosphoryl-β-D-ribose 2′-epimerase or Q203, an inhibitor with high specificity for the cytochrome bc1 complex, are in clinical trials. Despite these apparent progresses, there is an urgent need for new anti-TB drugs.

Enzymes belonging to the FAS-II system required for the biosynthesis of mycolic acids represent promising targets for the discovery of new anti-TB drugs. Among them, the enoyl ACP-reductase InhA is the primary target of the first-line anti-TB drug INH. INH acts as a prodrug that requires activation by the catalase-peroxidase KatG. For several years, resistance has been observed for INH, mainly due to mutations on KatG. So, to overcome these resistance problems, direct inhibitors of InhA, requiring no prior activation step, have been designed and shown to be promising, such as triclosan (TCL) derivatives [5,6,7,8,9,10], rhodanines [11], Genz-10850 (also called GEQ) and analogs [12,13], pyridomycin [14,15], and 4-hydroxy-2-pyridones [16]. 

**Figure 1:**
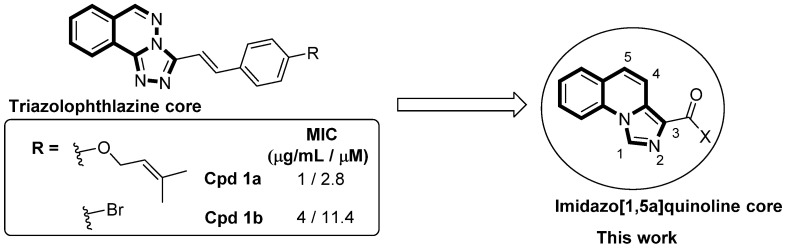
Design of imidazo [1,5-*a*]quinoline inhibitors.

We recently described the synthesis of derivatives bearing triazole scaffolds exhibiting promising anti-TB activities [17,18,19]. Among all derivatives investigated so far, tricyclic triazolophthalazine compounds, synthesized in a few steps, were found to be the most interesting. Indeed, a series of three-substituted triazolophthalazines were evaluated for their antimycobacterial activities. Among them, compounds **Cpd 1a** and **Cpd 1b** have been shown to inhibit the MTB strain H37Rv with a minimum inhibitory concentration (MIC) of less than 5 µg/mL (Figure 1).

Based on these results, we decided to explore imidazoquinoline as an isostere of the triazolophthalazine scaffold. Imidazo [1,5-a]quinoline, as for triazolophthalazine, is a tricyclic core found in many heterocyclic compounds that have shown several applications in the medicinal and material chemistry fields [20]. Some of them have been designed and synthesized as neurokinin (NK1) receptor ligands [21] or highly potent ligands of central benzodiazepine receptors [22]. 

Therefore, we envisioned two purposes for these imidazoquinoline molecules: (1) as novel pharmacophores for InhA inhibitors and (2) as isostere scaffolds of triazolophthalazines to achieve inhibition of mycobacterial strains.

## 2. Results

### 2.1. Chemistry

**Scheme 1:**
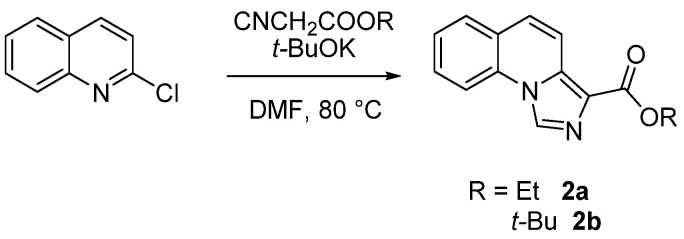
Synthesis of esters **2a** and **2b**.

**Scheme 2:**
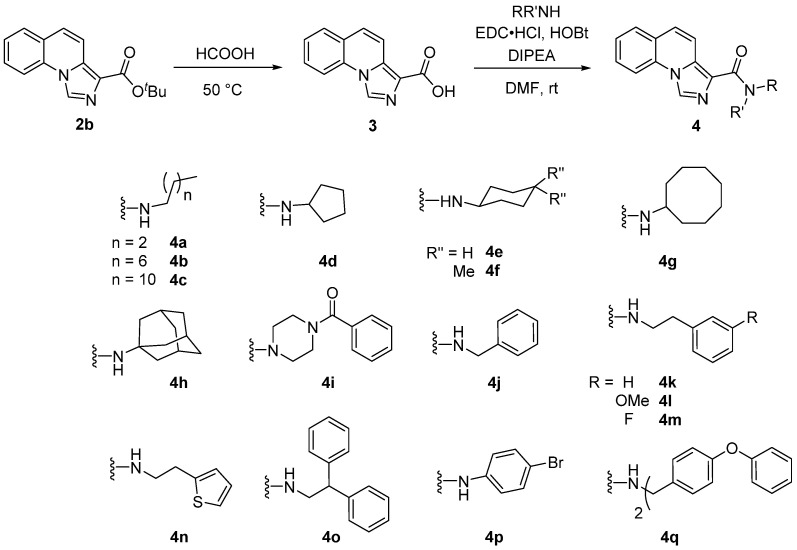
Synthesis of amide derivatives **4a**–**4p**.

One of the limitations for the synthesis of triazolophthalazine derivatives was their low solubility, which makes chemical modifications difficult. On the other hand, functionalized imidazoquinoline derivatives could be easily synthesized in two steps with the introduction of an ester or amide group in position 3 on imidazole. As outlined in Scheme 1 and Scheme 2, we considered the preparation of triazolophthalazine-like compounds bearing different amide groups directly bound to the heterocyclic moiety. Imidazo [1,5a]quinoline could be easily prepared by different reported methods [22,23].

**Figure 2:**
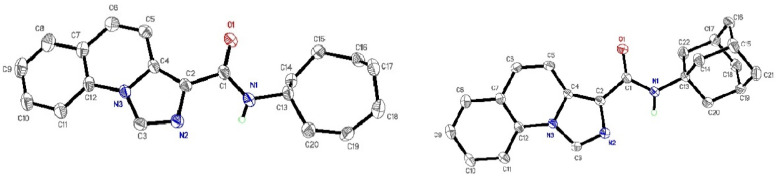
Molecular view of the X-ray crystal structures of amides **4g** (**Left**) and **4h** (**right**) (Scheme 2), with thermal ellipsoids drawn at the 30% probability level. Except for H atom on N1, hydrogen atoms were omitted for clarity.

**Scheme 3:**
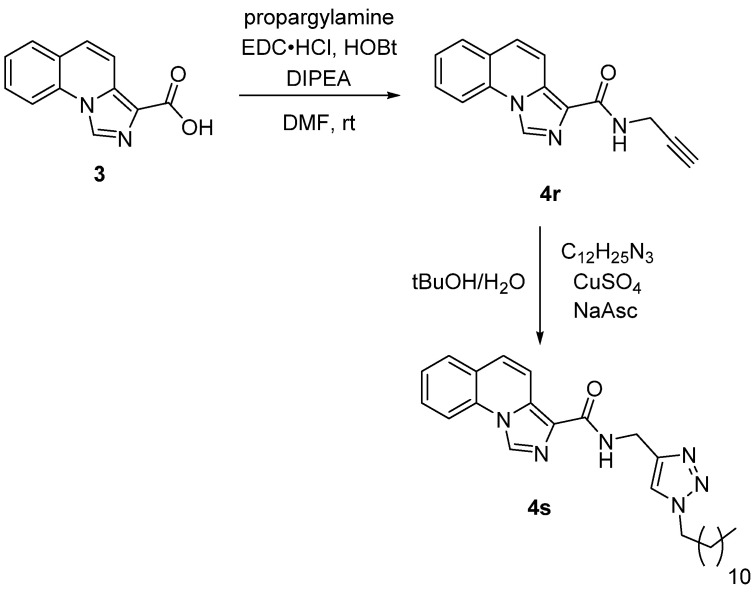
Synthesis of amide derivatives **4r**–**4s**.

The general synthesis of imidazoquinoline compounds **4** is outlined in Scheme 1, Scheme 2 and Scheme 3. Esters **2a** and **2b** were synthesized in one step from commercially available 2-chloroquinoline through an imidazole annulation with ethyl isocyanoacetate or tert-butyl isocyanoacetate in the presence of potassium tert-butoxide (Scheme 1). After hydrolysis of ester **2b** in acidic conditions, compound **3** was coupled to different cyclic and acyclic amines using the EDC•HCl/HOBt couple as a coupling agent in the presence of the Hünig base DIPEA (Scheme 2). The structures of compounds **4g** and **4h** were confirmed by X-ray crystallography (Figure 2) [24].

The alkyne derivative **4r**, obtained from compound **3** and propargylamine, was further coupled to dodecyl azide by Huisgen Cu(I)-catalyzed alkyne-azide cycloaddition to afford compound **4s** in 66% yield (Scheme 3).

### 2.2. Biological Activity

#### 2.2.1. InhA Inhibition

Compounds **4** were then evaluated as inhibitors of MTB InhA. These molecules can combine both the presence of hydrophobic chains that can interact with the hydrophobic pocket of InhA and an amide bond that has the potential to interact with tyrosine 158 of the protein. Triclosan was used as a control (Table 1).

Different kinds of substituents on the amide group were screened in an attempt to obtain active molecules. Except for compounds **4d** (bearing a cyclopentenyl moiety), **4h** (adamantyl), **4i** (benzoylpiperazinyl), and **4l** and **4m** (substituted phenethyl), which exhibit poor inhibition activity of 10% or less, all other compounds were found to significantly inhibit the enzymatic reaction at 50 µM but with activities much lower than that obtained with triclosan. Even compound **4q**, which possesses the biarylether moiety mimicking the two molecules of triclosan able to bind in the substrate binding site of InhA, is not very active, with only 34% inhibition at 50 µM. The derivatives showing the most inhibitory activity were compounds **4a** (C3-alkyl chain, 40% inhibition), **4r** (propargyl, 49% inhibition), and **4n** bearing a thiophene moiety with 66% inhibition.

#### 2.2.2. Inhibition of MTB Growth

**Figure 3:**
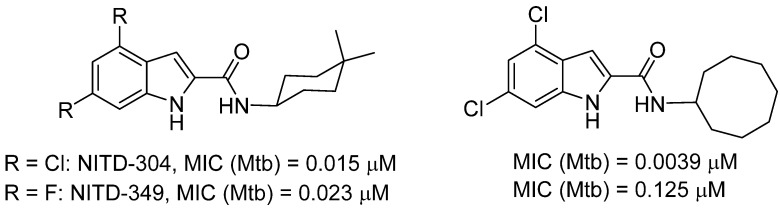
Inhibitors of MmpL3.

The newly synthesized imidazoquinoline compounds were evaluated for their inhibitory activity against the MTB H37Rv strain, using a dilution method. Minimum inhibitory concentrations (MICs) are shown in Table S1 (Supplementary Materials). Streptomycin was used as a control for MTB H37Rv (MIC = 0.25 µg/mL) strain. None of them showed antimycobacterial activities below 40 μg/mL, i.e., the cut-off concentration used in the tests. It has to be noted that compound **4p**, analog to **Cpd1b** (Figure 1), showed no inhibitory activity against the mycobacteria. Even compounds **4g** and **4h,** structurally related to some molecules inhibiting mycobacterial membrane protein Large 3 (MmpL3), such as indole derivatives NITD-304 or NIT-349 [25,26], were found to be not active (Figure 3). From these results, it appears that the imidazoquinoline moiety seems incompatible and detrimental to good activity against mycobacterial strains. This lack of activity may be due to the inability of this heterocyclic core to cross the membrane cell wall or the extrusion out of the bacterial cell by efflux. 

### 2.3. Docking Studies

**Figure 4:**
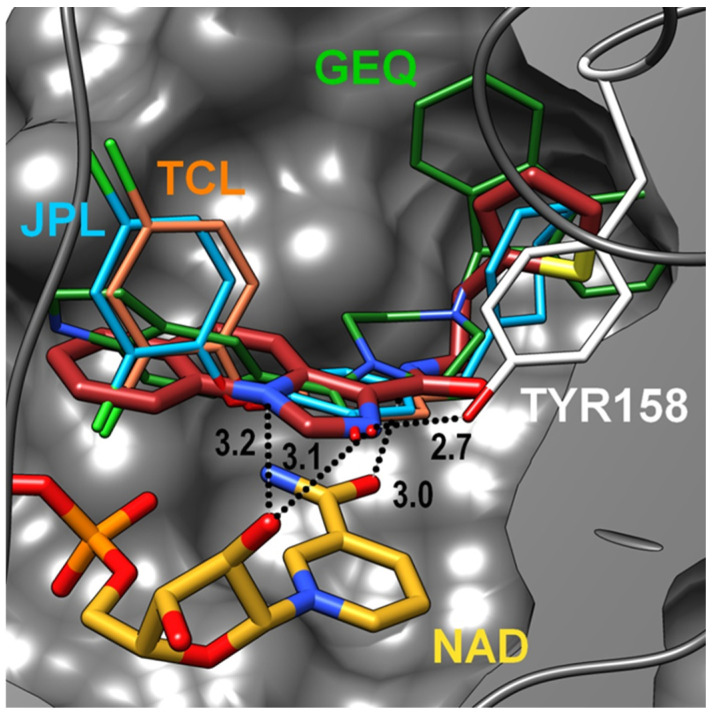
Best docked pose of compound **4n** in 1P45: A structure. The protein (ribbons and molecular surface) is depicted in gray, compound **4n** in dark red, the cofactor (NAD^+^, lower part) in gold, and the TYR158 in white. The reference ligands are shown using small sticks, orange for TCL500 (from 1P45:B), blue for JPL (from 3FNG), and green for GEQ (from 1P44). The interatomic distances are expressed in angstroms.

In order to give insights on activity, docking experiments were carried out on compound **4n**, which was found to have the best inhibitory activity on InhA. The best ranking docking poses (four of the five first OPT protocol poses, three of five for MSE protocol, see Experimental Section for details) are those corresponding to a conformation in which the imidazoquinoline ring interacts with the nicotinamide group of NAD^+^ through a π-stacking interaction and is positioned towards the entry of the major portal (Figure 4). These docking poses can reasonably be considered acceptable (best poses for both protocols) according to this ligand–cofactor interaction and to the limited fluctuations of the thiophene group. It should also be noted that the position of **4n** in the substrate binding site is comparable to that of the ligand GEQ co-crystallized in InhA (PDB entry 1P44) [13], that is, the thiophene moiety is positioned in the same way as the central five-membered cycle of the carbazole. Moreover, the tilted imidazoquinoline scaffold is positioned in the same place as the indole part of the GEQ. Compared to triclosan derivatives (i.e., JPL from 3FNG [27] or TCL from 1P45 [13]), it is interesting to underline that the imidazoquinoline heterocycle is in the same plane than the phenol ring of these TCL ligands and that the nitrogen atoms are positioned in approximatively the same positions than the oxygens atoms of TCL, JPL and GEQ, sharing the same hydrogen bonds with ribose of the cofactor. The interaction with the positively charged nitrogen of the nicotinamide group has disappeared, but the interaction with TYR158 is maintained. A hydrogen bond is probably also formed between the nitrogen of the amide group of compound **4n** and the oxygen of the nicotinamide group of the cofactor. An additional π-sulfur type interaction of the thiophene group and MET155 and MET199 could also be involved.

## 3. Experimental

### 3.1. Materials and Methods

All chemicals were purchased from Aldrich-Sigma (Burlington, MA, USA) or Alfa-Aesar (Ward Hill, MA, USA) and used without further purification. Anhydrous solvents were freshly distilled before use or were obtained from the M. Braun Solvent Purification System (MB-SPS-800). ^1^H NMR spectra were recorded on a Bruker spectrometer at 300 or 500 MHz for ^1^H NMR experiments and at 75 or 125 MHz for ^13^C NMR experiments. For ^1^H NMR and ^13^C NMR, the residual solvent was used as an internal reference: CDCl_3_ δ = 7.26 ppm, 77.0 ppm and DMSO = 2.50 ppm, 39.52 ppm. Proton coupling patterns are abbreviated as follows: s (singlet), d (doublet), t (triplet), q (quartet), and m (multiplet). Coupling constants (J) are reported in Hz. High-resolution mass spectra (HRMS) were recorded with an MAT 95XL spectrometer (ThermoFisher, Waltham, MA, USA) or on a UPLC Xevo G2 Q-TOF Waters using electrospray ionization methods. Desired products were purified by flash column chromatography with puriFlash 430 system using puriFlash^®^ columns from Interchim (Montluçon, France). 

### 3.2. Chemistry

#### 3.2.1. General Procedure for the Synthesis of Imidazole Compounds

To a solution of potassium *tert*-butoxide (4.58 mmol, 1.5 eq) in anhydrous DMF (10 mL/mmol) at 4 °C, 2-chloroquinoline (0.5 g, 3.05 mmol) and isocyanoacetate (4.58 mmol, 1.5 eq) were added. This reaction mixture was stirred for 30 min at 4 °C, then 1 h at room temperature, and finally 20 h at 80 °C. After cooling at room temperature, acetic acid (1 mL/mmol) was added and the mixture was stirred for 20 min. The resulting solution was poured into cooled water and the product was extracted with ethyl acetate. The organic phases were combined and were washed three times with water, dried over MgSO_4_, filtered, and concentrated under reduced pressure. The desired product was purified by flash chromatography.

Ethyl imidazo [1,5-a]quinoline-3-carboxylate (**2a**). The desired product was isolated after flash chromatography (petroleum ether/ethyl acetate 7/3 to 2/8 in 15 min) as a white powder (0.441 g, 78%). ^1^H NMR (300 MHz, CDCl_3_) δ 8.51 (s, 1H); 7.89 (t, *J* = 7.9 Hz, 1H); 7.62 (d, *J* = 7.8 Hz, 1H); 7.52 (td, *J* = 7.8 Hz, 1.3 Hz, 1H); 7.38 (t, *J* = 7.6 Hz, 1.1 Hz, 1H); 7.23 (d, *J* = 9.5 Hz, 1H); 4.40 (q, *J* = 7.2 Hz, 2H); 1.40 (t, *J* = 7.2 Hz, 3H); ^13^C NMR (75 MHz, CDCl_3_) δ 163.2; 132.4; 130.2; 129.3; 128.8; 127.8; 126.0; 125.6; 124.1; 123.8; 117.0; 114.6; 60.3; 14.4; HRMS Calculated for C_14_H_13_N_2_O_2_ (DCI-CH_4_, M+H^+^): 241.0977. Found: 241.0970.

*tert*-Butyl imidazo [1,5-a]quinoline-3-carboxylate (**2b**). The same protocol as for compound **2a** was used. The desired product was isolated after flash chromatography (petroleum ether/ethyl acetate 7/3 to 2/8 in 15 min) as a yellow solid upon standing (0.654 g, Yield 80%). ^1^H NMR (300 MHz, CDCl_3_) δ 8.55 (s, 1H); 7.95 (d, *J* = 9.6 Hz, 1H); 7.93 (d, *J* = 8.3 Hz, 1H); 7.67 (d, *J* = 7.9 Hz, 1.5 Hz, 1H); 7.55 (td, *J* = 7.9 Hz, 1.5 Hz, 1H); 7.41 (td, *J* = 7.6 Hz, 1.1 Hz, 1H); 7.27 (d, *J* = 9.6 Hz, 1H); 1.65 (s, 9H); ^13^C NMR (75 MHz, CDCl_3_) δ 162.6; 132.0; 130.3; 129.3; 128.8; 127.7; 126.0; 125.5; 125.3; 123.9; 117.5; 114.6; 81.0; 28.4; HRMS Calculated for C_16_H_16_N_2_O_2_ (DCI-CH_4_, M): 268.1212. Found: 268.1208. 

Imidazo [1,5-a]quinoline-3-carboxylic acid (**3**). A solution of compound **2b** (0.273 g, 3.72 mmol) in formic acid (2 mL) was stirred at 50 °C for overnight. After reaction completion as indicated by TLC analysis, the reaction mixture was evaporated to dryness. The crude product was used in the next step without further purification

#### 3.2.2. General Procedure for the Coupling of the Different Amines with Compound **3** (**4a**–**4s**)

Typically, to a solution of compound **3** (0.070 g, 0.330 mmol) in anhydrous DMF at 4 °C, N-(3-dimethylaminopropyl)-N′-ethylcarbodiimide hydrochloride, EDC•HCl (0. 396 mmol, 1.2 eq), HOBt (0.396 mmol, 1.2 eq), amine (0.396 mmol, 1.2 eq), and DIPEA (0.825 mmol, 2.5 eq) were successively added. The reaction mixture was allowed to warm up to room temperature and was stirred for 20 h. Water was added and the product was extracted with ethyl acetate. The organic phases were combined and were washed three times with water, dried over MgSO_4_, filtered, and concentrated under reduced pressure. The desired product was purified by flash chromatography.

N-Butylimidazo [1,5-a]quinoline-3-carboxamide (**4a**). The desired product was isolated after flash chromatography (petroleum ether/ethyl acetate 7/3 to 2/8 in 15 min) as a white powder (52 mg, 57%). R_f_ = 0.41 (petroleum ether/ethyl acetate 6/4). ^1^H NMR (300 MHz, CDCl_3_) δ 8.50 (s, 1H); 8.20 (d, *J* = 9.5 Hz, 1H); 7.97 (d, *J* = 8.3 Hz, 1H); 7.73 (dd, *J* = 7.9 Hz, 1.4 Hz, 1H); 7.60 (td, *J* = 7.7 Hz, 1.4 Hz, 1H); 7.47 (td, *J* = 7.5 Hz, 1.1 Hz, 1H); 7.28 (d, *J* = 9.6 Hz, 1H); 7.24 (br peak, 1H); 13C NMR (75 MHz, CDCl_3_) δ 163.3; 130.5; 130.1; 129.1; 127.2; 126.3; 126.1; 124.6; 124.3; 118.2; 114.6; 38.6; 32.0; 20.2; 13.8; HRMS Calculated for C_16_H_18_N_3_O (DCI-CH_4_, M+H^+^): 268.1450. Found: 268.1443. Purity (HPLC): >99% at 254 nm (tR = 2.18 min).

N-Octylimidazo [1,5-a]quinoline-3-carboxamide (**4b**). The desired product was isolated after flash chromatography (petroleum ether/ethyl acetate 8/2 to 2/8 in 15 min) as a white powder (70 mg, 70%). R_f_ = 0.47 (petroleum ether/ethyl acetate 6/4). 1H NMR (300 MHz, CDCl_3_) δ 8.49 (s, 1H); 8.20 (d, *J* = 9.4 Hz, 1H); 7.96 (d, *J* = 8.2 Hz, 1H); 7.72 (dd, *J* = 7.8 Hz, 1.2 Hz, 1H); 7.59 (td, *J* = 7.8 Hz, 1.3 Hz, 1H); 7.46 (td, *J* = 7.6 Hz, 1.1 Hz, 1H); 7.23–7.29 (m, 2H); 3.47 (q, *J* = 6.7 Hz, 2H); 1.64 (m, 2H); 1.20–1.52 (m, 10 H); 0.86 (t, *J* = 6.7 Hz, 3H); 13C NMR (75 MHz, CDCl_3_) δ 163.3; 130.5; 130.1; 129.0; 127.2; 126.3; 126.0; 124.6; 124.3; 118.2; 114.6; 38.9; 31.8; 29.9; 29.3; 29.2; 27.0; 22.6; 14.0; HRMS Calculated for C_20_H_25_N_3_O (DCI-CH_4_, M+H^+^): 324.2076. Found: 324.2065. Purity (HPLC): >99% at 254 nm (tR = 4.62 min).

N-Dodecylimidazo [1,5-a]quinoline-3-carboxamide (**4c**). The desired product was isolated after flash chromatography (petroleum ether/ethyl acetate 7/3 to 2/8 in 15 min) as a white powder (92 mg, 69%). ^1^H NMR (300 MHz, CDCl_3_) δ 8.49 (s, 1H); 8.20 (d, *J* = 9.7 Hz, 1H); 7.96 (d, *J* = 8.2 Hz, 1H); 7.73 (dd, *J* = 7.8 Hz, 1.4 Hz, 1H); 7.56–7.62 (m, 1H); 7.44 -749 (m, 1H); 7.28 (d, *J* = 9.7 Hz, 1H); 7.22–7.26 (br m, 1H); 3.47 (m, 2H); 1.65 (m, 2H); 1.24 -1.43 (m, 18H); 0.85 (t, *J* = 6.6 Hz, 3H); ^13^C NMR (75 MHz, CDCl_3_) δ 163.3; 130.5; 130.1; 129.1; 127.2; 126.4; 126.1; 124.6; 124.3; 118.2; 114.6; 38.9; 31.9; 29.9; 29.61; 29.59; 29.57; 29.53; 29.4; 29.3; 27.0; 22.6; 14.1; HRMS Calculated for C_24_H_34_N_3_O (DCI-CH_4_, M+H^+^): 380.2702. Found: 380.2697. Purity (HPLC): 98% at 254 nm (tR = 9.57 min).

N-Cyclopentylimidazo [1,5-a]quinoline-3-carboxamide (**4d**). The desired product was isolated after flash chromatography (petroleum ether/ethyl acetate 8/2 to 2/8 in 15 min) as a white powder (55 mg, 60 %). R_f_ = 0.35 (petroleum ether/ethyl acetate 6/4). ^1^H NMR (300 MHz, CDCl_3_) **δ** 8.48 (s, 1H); 8.20 (d, *J* = 9.6 Hz, 1H); 7.96 (d, *J* = 8.3 Hz, 1H); 7.72 (dd, *J* = 7.8 Hz, 1.1 Hz, 1H); 7.59 (td, *J* = 7.7 Hz, 1.3Hz, 1H); 7.46 (td, *J* = 7.6 Hz, 1.1 Hz, 1H); 7.28 (d, *J* = 9.6 Hz, 1H); 7.18 (d, *J* = 7.3 Hz, 1H); 4.46 (m, 1H); 2.10 (m, 2h); 1.60–1.76 (m, 6H); ^13^C NMR (75 MHz, CDCl_3_) **δ** 162.9; 130.5; 130.1; 127.2; 126.3; 126.1; 124.6; 124.3; 118.2; 114.6; 50.6; 33.3; 23.8; HRMS Calculated for C_17_H_18_N_3_O (DCI-CH_4_, M+H^+^): 280.1450. Found: 280.1439. Purity (HPLC): >99% at 254 nm (tR = 2.22 min).

N-Cyclohexylimidazo [1,5-a]quinoline-3-carboxamide (**4e**). The desired product was isolated after flash chromatography (petroleum ether/ethyl acetate 8/2 to 2/8 in 15 min) as a white powder (71 mg, 71%). R_f_ = 0.45 (petroleum ether/ethyl acetate 6/4). ^1^H NMR (300 MHz, CDCl_3_) δ 8.47 (s, 1H); 8.18 (d, *J* = 9.4 Hz, 1H); 7.94 (d, *J* = 8.4 Hz, 1H); 7.70 (dd, *J* = 7.9 Hz, 1.3 Hz, 1H); 7.57 (td, *J* = 7.8; 1.5 Hz, 1H); 7.44 (td, *J* = 7.6 Hz, 1.1 Hz, 1H); 7.26 (d, *J* = 9.4 Hz, 1H); 7.13 (d, *J* = 8.3 Hz, 1H); 4.00 (m, 2H); 2.04 (m, 2H); 1.75 (m, 2H); 1.63 (m, 1H); 1.15–1.50 (m, 5H); ^13^C NMR (75 MHz, CDCl_3_) δ 162.4; 130.5; 130.1; 129.0; 127.3; 126.3; 126.0; 124.5; 124.3; 118.2; 114.5; 47.6; 33.3; 25.6; 25.0; HRMS Calculated for C_18_H_20_N_3_O (DCI-CH_4_, M+H^+^): 294.1606. Found: 294.1597. Purity (HPLC): >99% at 254 nm (tR = 2.79 min).

N-(4,4-Dimethylcyclohexyl)imidazo [1,5-a]quinoline-3-carboxamide (**4f**). The desired product was isolated after flash chromatography (petroleum ether/ethyl acetate 8/2 to 2/8 in 15 min) as a white powder (82 mg, 77%). R_f_ = 0.59 (petroleum ether/ethyl acetate 5/5). ^1^H NMR (300 MHz, CDCl_3_) δ 8.47 (s, 1H); 8.17 (d, *J* = 9.7 Hz, 1H); 7.93 (d, *J* = 8.3 Hz, 1H); 7.69 (dd, *J* = 7.8 Hz, 1.2 Hz, 1H); 7.56 (m, 1H); 7.44 (m, 1H); 7.25 (d, *J* = 9.5 Hz, 1H); 7.15 (d, *J* = 8.3 Hz, 1H); 3.95 (m, 1H); 1.89 (m, 2H); 1.30–1.57 (m, 6H); 0.94 (s, 3H); 0.93 (s, 3H); ^13^C NMR (75 MHz, CDCl_3_) δ 162.6; 130.5; 130.1; 129.0; 127.2; 126.0; 124.6; 124.2; 118.1; 114.5; 47.8; 37.7; 31.6; 29.5; 28.9; 25.0; HRMS Calculated for C_2_0H_24_N_3_O (DCI-CH4, M+H+): 322.1919. Found: 322.1917. Purity (HPLC): >99% at 254 nm (tR = 2.22 min).

N-Cyclooctylimidazo [1,5-a]quinoline-3-carboxamide (**4g**). The desired product was isolated after flash chromatography (petroleum ether/ethyl acetate 7/3 to 2/8 in 15 min) as a white powder (46 mg, 66%). Crystallization (dichloromethane). R_f_ = 0.72 (petroleum ether/ethyl acetate 4/6). ^1^H NMR (300 MHz, CDCl_3_) δ 8.49 (s, 1H); 8.20 (d, *J* = 9.5 Hz, 1H); 7.95 (d, *J* = 8.4 Hz, 1H); 7.71 (dd, *J* = 7.8 Hz, 1.2 Hz, 1H); 7.59 (td, *J* = 7.8 Hz, 1.5 Hz, 1H); 7.46 (td, *J* = 7.6 Hz, 1.2 Hz, 1H); 7.27 (d, *J* = 9.5 Hz, 1H); 7.21 (d, *J* = 8.2 Hz, 1H); 4.24 (m, 1H); 1.97 (m, 2H); 1.60 (m, 12H); 13C NMR (75 MHz, CDCl_3_) δ 162.3; 130.5; 130.1; 129.04; 129.02; 127.3; 126.3; 126.0; 124.6; 124.3; 118.2; 114.6; 48.7; 32.5; 27.2; 25.5; 23.8; HRMS Calculated for C_20_H_24_N_3_O (DCI-CH_4_, M+H^+^): 322.1919. Found: 322.1906. Purity (HPLC): >99% at 254 nm (tR = 3.84 min).

N-(Adamantan-1-yl)imidazo [1,5-a]quinoline-3-carboxamide (**4h**). The desired product was isolated after flash chromatography (petroleum ether/ethyl acetate 7/3 to 2/8 in 15 min) as a white powder (47 mg, 39%). R_f_ = 0.49 (petroleum ether/ethyl acetate 6/4). ^1^H NMR (300 MHz, CDCl_3_) δ 8.47 (s, 1H); 8.18 (d, *J* = 9.6 Hz, 1H); 7.95 (d, *J* = 8.3 Hz, 1H); 7.71 (dd, *J* = 7.8 Hz, 1.3 Hz, 1H); 7.58 (td, *J* = 7.8 Hz, 1.5 Hz, 1H); 7.46 (td, *J* = 7.8 Hz, 1.2 Hz, 1H); 7.25 (d, *J* = 7.6 Hz, 1H); 2.20 (m, 6H); 2.13 (m, 3H); 1.68–1.78 (m, 6H); ^13^C NMR (75 MHz, CDCl_3_) δ 162.8; 130.6; 129.9; 129.0; 127.9; 126.0; 124.4; 124.3; 118.3; 114.6; 51.7; 41.9; 36.4; 29.5; HRMS Calculated for C_22_H_24_N_3_O (DCI-CH_4_, M+H^+^): 346.1919. Found: 346.1919. Purity (HPLC): 97% at 254 nm (tR = 4.51 min).

1-Benzoyl-4-({imidazo [1,5-a]quinolin-3-yl}carbonyl)piperazine (**4i**). The desired product was isolated after flash chromatography (petroleum ether/ethyl acetate 2/8 to 100% ethyl acetate in 15 min) as a white powder (94 mg, 74%). R_f_ = 0.27 (ethyl acetate). ^1^H NMR (300 MHz, CDCl_3_) δ 8.53 (br s, 1 H); 8.09 (d, *J* = 9.5 Hz, 1H); 7.99 (d, *J* = 8.4 Hz, 1H); 7.74 (dd, *J* = 7.7 Hz, 1.1 Hz, 1H); 7.61 (m, 1H); 7.48 (m, 1H); 7.43 (br m, 5H); 7.30 (d, *J* = 9.5 Hz, 1H); 3.59–4.41 (m, 8H); ^13^C NMR (75 MHz, CDCl_3_) δ 170.6; 163.5; 135.4; 133.0; 130.4; 129.9; 129.2; 129.0; 128.5; 127.3; 127.0; 126.2; 124.8; 124.3; 118.2; 114.6; The carbons of the piperazine ring are missing. Calculated for C_23_H_21_N_4_O_2_ (DCI-CH_4_, M+H^+^): 385.1665. Found: 385.1655. Found: 385.1655. Purity (HPLC): 94% at 254 nm (tR = 1.51 min).

N-Benzylimidazo [1,5-a]quinoline-3-carboxamide (**4j**). The desired product was isolated after flash chromatography (petroleum ether/ethyl acetate 8/2 to 2/8 in 15 min) as a white powder (81 mg, 91%). R_f_ = 0.44 (petroleum ether/ethyl acetate 6/4). 1H NMR (300 MHz, CDCl_3_) δ 8.47 (s, 1H); 8.23 (d, *J* = 9.6 Hz, 1H); 7.95 (d, *J* = 8.6 Hz, 1H); 7.74 (dd, *J* = 7.8 Hz, 1.2 Hz, 1H); 7.58–7.63 (m, 2H); 7.48 (td, *J* = 7.6 Hz, 1.1 Hz, 1H); 7.24–7.42 (m, 6H); 4.69 (d, *J* = 5.9 Hz, 2H); 13C NMR (75 MHz, CDCl_3_) δ 163.2; 138.7; 130.5; 130.3; 128.6; 127.8; 127.3; 126.9; 126.5; 126.1; 124.8; 124.3; 118.1; 114.6; 42.9; HRMS Calculated for C_19_H_16_N_3_O (DCI-CH_4_, M+H^+^): 302.1293. Found: 302.1288. Purity (HPLC): >99% at 254 nm (tR = 2.36 min).

N-(2-Phenylethyl)imidazo [1,5-a]quinoline-3-carboxamide (**4k**). The desired product was isolated after flash chromatography (petroleum ether/ethyl acetate 8/2 to 2/8 in 15 min) as a white powder (88 mg, 87 %). R_f_ = 0.36 (petroleum ether/ethyl acetate 6/4). ^1^H NMR (300 MHz, CDCl_3_) δ 8.48 (s, 1H); 8.21 (d, *J* = 9.5 Hz, 1H); 7.96 (d, *J* = 8.3 Hz, 1H); 7.73 (dd, *J* = 7.8 Hz, 1.2Hz, 1H); 7.60 (td, *J* = 7.8 Hz, 1.5 Hz, 1H); 7.47 (td, *J* = 7.6 Hz, 1.2 Hz, 1H); 7.19–7.38 (m, 7H); 3.75 (m, 2H); 2.97 (t, *J* = 7.4 Hz, 1H); ^13^C NMR (75 MHz, CDCl_3_) δ 163.3; 139.2; 130.5; 130.2; 129.1; 129.07; 128.8; 128.6; 127.0; 126.43; 126.35; 126.1; 124.8; 124.3; 118.1; 114.6; 40.3; 36.2; HRMS Calculated for C_19_H_18_N_3_O (DCI-CH_4_, M+H^+^): 316.1450. Found: 316.1449. Purity (HPLC): >99% at 254 nm (tR = 2.64 min).

N-[2-(3-Methoxyphenyl)ethyl]imidazo [1,5-a]quinoline-3-carboxamide (**4l**). The desired product was isolated after flash chromatography (petroleum ether/ethyl acetate 8/2 to 2/8 in 15 min) as a white powder (72 mg, 77%). R_f_ = 0.32 (petroleum ether/ethyl acetate 4/6). ^1^H NMR (300 MHz, CDCl_3_) δ 8.48 (s, 1H); 8.21 (d, *J* = 9.5 Hz, 1H); 7.96 (d, *J* = 8.2 Hz, 1H); 7.74 (dd, *J* = 7.8 Hz, 1.2 Hz, 1H); 7.61 (m, 1H); 7.48 (m, 1H); 7.36 (m, 1H); 7.32 (d, *J* = 9.6 Hz, 1H); 7.22 (d, *J* = 7.8 Hz, 1H); 6.76–6.88 (m, 3H); 3.79 (s, 3H); 3.75 (m, 2H); 2.95 (t, *J* = 7.4 Hz, 2H); ^13^C NMR (75 MHz, CDCl3) δ 163.4; 159.7; 140.8; 130.6; 130.2; 129.5; 129.1; 127.1; 126.5; 126.1; 124.8; 124.3; 121.1; 118.1; 114.6; 114.2; 112.1; 55.2; 40.2; 36.3; HRMS Calculated for C_21_H_20_N_3_O_2_ (DCI-CH_4_, M+H^+^): 346.1556. Found: 346.1562. Purity (HPLC): 98% at 254 nm (tR = 2.57 min).

N-[2-(3-Fluorophenyl)ethyl]imidazo [1,5-a]quinoline-3-carboxamide (**4m**). The desired product was isolated after flash chromatography (petroleum ether/ethyl acetate 8/2 to 2/8 in 15 min) as a white powder (98 mg, 89%). R_f_ = 0.39 (petroleum ether/ethyl acetate 5/5). ^1^H NMR (300 MHz, CDCl_3_) δ 8.48 (s, 1H); 8.19 (d, *J* = 9.6Hz, 1H); 7.95 (d, *J* = 8.3 Hz, 1H); 7.72 (dd, *J* = 7.8 Hz, 1.3 Hz, 1H); 7.59 (m, 1H); 7.47 (m, 1H); 7.37 (t broad, *J* = 5.9 Hz, 1H); 7.29 (d, *J* = 9.6 Hz, 1H); 7.2–7.30 (m, 1H); 7.04 (d, *J* = 7.7 Hz, 1H); 6.98 (m, 1H); 6.88–6.94 (m, 1H); 3.73 (m, 2H); 2.96 (t, *J* = 7.4 Hz, 2H); ^13^C NMR (75 MHz, CDCl_3_) δ 163.3; 162.9 (d, *J* = 94.9 Hz); 141.7 (d, *J* = 7.2 Hz); 130.5; 130.2; 130.0 (d, *J* = 8.3 Hz); 129.14; 129.06; 126.8; 126.4; 126.1; 124.9; 124.4 (d, *J* = 2.8 Hz); 124.3; 118.0; 115.6 (d, *J* = 21.0 Hz); 114.6; 113.2 (*J* = 20.9 Hz); 40.0; 35.9 (d, *J* = 1.6 Hz); ^19^F NMR (282 MHz, CDCl_3_) δ −113.4; HRMS Calculated forC_20_H_17_N_3_OF (DCI-CH_4_, M+H^+^): 334.1356. Found: 334.1348. Purity (HPLC): 97% at 254 nm (tR = 3.01 min).

N-[2-(Thiophen-2-yl)ethyl]imidazo [1,5-a]quinoline-3-carboxamide (**4n**). The desired product was isolated after flash chromatography (petroleum ether/ethyl acetate 7/3 to 2/8 in 15 min) as a brownish powder (72 mg, 68%). ^1^H NMR (300 MHz, CDCl_3_) δ 8.47 (s, 1H); 8.19 (d, *J* = 9.6 Hz, 1H); 7.95 (d, *J* = 8.3 Hz, 1H); 7.72 (dd, *J* = 7.8 Hz, 1.2 Hz, 1H); 7.56–7.62 (m, 1H); 7.44–7.47 (m, 1H); 7.40 (br m, 1H); 7.29 (d, *J* = 9.6 Hz, 1H); 7.16 (dd, *J* = 5.0 Hz, 1.3 Hz, 1H); 6.94–6.96 (m, 1H); 6.90 (m, 1H); 3.74–3.80 (m, 2H); 3.18 (t, *J* = 7.0 Hz, 2H); ^13^C NMR (75 MHz, CDCl_3_) δ 163.3; 141.5; 130.5; 130.3; 129.2; 129.1; 126.8; 126.5; 126.2; 125.3; 124.9; 124.3; 123.8; 118.0; 114.6; 40.4; 30.4; HRMS Calculated for C_18_H_16_N_3_OS (DCI-CH_4_, M+H^+^): 322.1014. Found: 322.1015. Purity (HPLC): >99% at 254 nm (tR = 2.67 min).

N-(2,2-Diphenylethyl)imidazo [1,5-a]quinoline-3-carboxamide (**4o**). The desired product was isolated after flash chromatography (petroleum ether/ethyl acetate 70/30 to 0/100 in 15 min) as a white powder (109 mg, 84%). ^1^H NMR (300 MHz, CDCl_3_) δ 8.35 (s, 1H); 8.18 (d, *J* = 9.5 Hz, 1H); 7.85 (d, *J* = 8.2 Hz, 1H); 7.68 (dd, *J* = 7.8 Hz, 1.2 Hz, 1H); 7.51–7.57 (m, 1H); 7.43 (m, 1H); 7.19–7.33 (m, 12H); 4.39 (t, *J* = 8.0 Hz, 1H); 4.13–4.17 (m, 2H); 13C NMR (75 MHz, CDCl_3_) δ 163.3; 124.1; 130.4; 130.1; 129.0; 128.9; 128.6; 128.1; 126.6; 126.0; 124.6; 124.1; 117.9; 114.5; 50.9; 43.3; HRMS Calculated for C_26_H_22_N_3_O: 392.1776 (DCI-CH_4_, M+H+). Found: 392.1772. Purity (HPLC): 99% at 254 nm (tR = 4.61 min).

N-(4-Bromophenyl)imidazo [1,5-a]quinoline-3-carboxamide (**4p**). The desired product was isolated after flash chromatography (petroleum ether/ethyl acetate 7/3 to 2/8 in 15 min) as a white powder (69 mg, 51%). R_f_ = 0.44 (petroleum ether/ethyl acetate 6/4). ^1^H NMR (500 MHz, DMSO) δ 10.29 (s, 1H); 9.34 (s, 1H); 8.56 (d, *J* = 8.2 Hz, 1H); 8.10 (d, *J* = 9.5 Hz, 1H); 7.97 (dd, *J* = 7.9 Hz, 1.2Hz, 1H); 7.92 (d, *J* = 8.9 Hz, 2H); 7.77 (td, *J* = 8.5 Hz, 1.3 Hz, 1H); 7.59–7.62 (m, 2H); 7.51 (d, *J* = 8.9 Hz, 2H); ^13^C NMR (125 MHz, DMSO) δ 161.4; 138.5; 131.3; 130.41; 130.39; 129.8; 129.0; 126.4; 126.1; 125.7; 123.7; 116.9; 115.9; 114.8; HRMS Calculated for C_18_H_13_BrN_3_O (ESI, M+H^+^): 366.0242. Found: 366.0238. Purity (HPLC): 98% at 254 nm (tR = 4.66 min).

N,N-Bis[(4-phenoxyphenyl)methyl]imidazo [1,5-a]quinoline-3-carboxamide (**4q**). The desired product was isolated after flash chromatography (petroleum ether/ethyl acetate 7/3 to 2/8 in 15 min) as a white powder (112 mg, 59%). R_f_ = 0.56 (petroleum ether/ethyl acetate 5/5). ^1^H NMR (300 MHz, CDCl_3_) δ 8.55 (s, 1H); 8.29 (d, *J* = 9.7 Hz, 1H); 7.99 (d, *J* = 8.2 Hz, 1H); 7.76 (dd, *J* = 7.8 Hz, 1.1 Hz, 1H); 7.62 (m, 1H); 7.49 (m, 1H); 7.30–7.35 (m, 9H); 7.09 (td, *J* = 8.5 Hz, 1.2 Hz, 2H); 6.97–7.03 (m, 8H); 5.41 (s, 2H); 4.71 (s, 2H); ^13^C NMR (75 MHz, CDCl_3_) δ 164.5; 157.26; 157.18; 156.46; 156.32; 133.1; 132.8; 132.6; 130.5; 129.8 (br peak); 129.7; 129.3 (br peak); 129.1; 129.0; 128.0; 126.1; 124.5; 124.4; 123.1; 119.0 (br peak); 118.8 (br peak); 114.6; 50.3 47.5; HRMS Calculated for C_38_H_30_N_3_O_3_ (DCI-CH_4_, M+H^+^): 576.2287. Found: 576.2293. Purity (HPLC): 97% at 254 nm (tR = 9.14 min).

N-(Prop-2-yn-1-yl)imidazo [1,5-a]quinoline-3-carboxamide (**4r**). The desired product was isolated after flash chromatography (petroleum ether/ethyl acetate 7/3 to 2/8 in 15 min) as a white powder (92 mg, 78%). R_f_ = 0.52 (petroleum ether/ethyl acetate 5/5). ^1^H NMR (300 MHz, CDCl_3_) δ 8.52 (s, 1H); 8.18 (d, *J* = 9.4 Hz, 1H); 7.99 (d, *J* = 8.4 Hz, 1H); 7.75 (dd, *J* = 7.7 Hz, 1.2 Hz, 1H); 7.63 (m, 1H); 7.50 (m, 1H); 7.42 (m, 1H); 7.33 (d, *J* = 9.4 Hz, 1H); 4.29 (dd, *J* = 5.5, 2.5 Hz, 2H); 2.26 (t, *J* = 2.4 Hz, 1H); ^13^C NMR (75 MHz, CDCl_3_) δ 163.0; 130.5; 129.3; 129.2; 126.6; 126.4; 126.2; 125.1; 124.3; 117.9; 114.7; 79.9; 71.3; 28.6; 1 carbon is missing. HRMS Calculated for C_15_H_12_N_3_O (DCI-CH_4_, M+H^+^): 250.0980. Found: 250.0971. Purity (HPLC): >99% at 254 nm (tR = 1.49 min).

N-[(1-Dodecyl-1H-1,2,3-triazol-4-yl)methyl]imidazo [1,5-a]quinoline-3-carboxamide (**4s**). The desired product was isolated after flash chromatography (petroleum ether/ethyl acetate 7/3 to 0/10 in 15 min) as a white powder (48 mg, 66%). R_f_ = 0.24 (petroleum ether/ethyl acetate 3/7). ^1^H NMR (300 MHz, CDCl_3_) δ 8.60 (s, 1H); 8.20 (d, *J* = 9.7 Hz, 1H); 8.03 (d, *J* = 8.2 Hz, 1H); 7.97 (t, *J* = 6.0 Hz, 1H); 7.76 (dd, *J* = 7.9 Hz, 1Hz, 1H); 7.61–7.67 (m, 2H); 7.51 (m, 1H); 7.33 (d, *J* = 9.6 Hz, 1H); 4.80 (d, *J* = 6.1 Hz, 2H); 4.33 (t, *J* = 7.1 Hz, 2H); 1.90 (m, 2H); 1.25 (m, 18H); 0.89 (m, 3H); ^13^C NMR (75 MHz, CDCl_3_) v 163.4; 145.4; 130.5; 130.3; 129.2; 129.0; 126.8; 126.7; 126.1; 124.9; 124.2; 122.1; 117.8; 114.7; 50.3; 34.5; 31.8; 30.2; 29.5; 29.4; 29.3; 29.2; 28.9; 26.4; 22.6; 14.1; HRMS Calculated for C_27_H_37_N_6_O (DCI-CH_4_, M+H^+^): 461.3029. Found: 461.3039. Purity (HPLC): 97% at 254 nm (tR = 7.84 min).

#### 3.2.3. Purity of the Compounds

The analyses were performed using UPLC PDA SQD Simple Quadrupole Mass Spectrometer (Waters, Milford, MA, USA). For LC separation, the chromatographic column was a BEH C18 column (100 mm × 2.1 mm, 1.7 μm, Waters); the mobile phase consisted of water (containing 0.1% formic acid) (A) and acetonitrile (containing 0.1% formic acid) (B) with a gradient elution at a flow rate of 0.3 mL/min. The gradient elution was set as follows: 0 min, 50% B; 1 min, 50% B; 11 min, 100% B; 12 min, 100% B; 13 min, 50% B. The column temperature was 40 °C. MS responses of target analytes were evaluated by electrospray ionization (ESI) in positive ion mode. Nitrogen was used as the nebulizer. The instrument settings were as follows: capillary voltage: 2.5 kV; cone voltage: 20V; de-solvent gas flow: 900 L/h; cone gas flow: 50 L/h; ion source temperature: 150 °C; solvent temperature: 450 °C. All final compounds had a purity greater than 94% as determined by LCMS analysis.

### 3.3. Molecular Docking Studies of Compound ***4n***

#### 3.3.1. Molecular Graphics

Molecular graphics were performed with the UCSF Chimera package [28]. Chimera was developed by the Resource for Biocomputing, Visualization, and Informatics at the University of California, San Francisco (supported by the NIGMS P41-GM103311). The protein structures used in this paper were downloaded from the RCSB Protein Database [29] and aligned [30] on a reference structure 1BVR (chain A, formerly 1BVR:A) [31]. The protein structures were prepared (structure checks, rotamers, hydrogenation, splitting of chains) using Biovia (www.3dsbiovia.com, accessed on 4 June 2024) Discovery Studio Visualizer 2021 (DSV), UCSF Chimera, and in-house Python codes. The new compounds were sketched using ChemAxon Marvin 16 (www.chemaxon.com, accessed on 4 June 2024). All ligands were checked (hybridization, hydrogenation, some geometry optimizations, 3D sketching) and merged in SDF libraries using DSV or in-house programs.

#### 3.3.2. Molecular Docking

Molecular modeling studies were carried with Molegro Virtual Docker 6 (www.molexus.io accessed on 4 June 2024) software using aligned 1P45 (chain A, formerly 1P45:A) [13] PDB structure, which is characterized by a binding site widely opened at the major portal side and a closed minor portal. 

Two molecular docking protocols (MSE, OPT) and two internal scoring schemes (Moldock and Rerank) [32] were combined in a multimodal (docking) and consensus (scoring) approach giving two sets of poses per ligand. The protocols share the same set of flexible residues: ALA154, ALA157, ALA164, ALA198, ALA201, ALA206, ARG195, ASN231, ASP148, ASP150, GLN214, GLU219, ILE202, ILE215, LEU217, LEU218, LYS165, MET98, MET103, MET155, MET161, MET199, MET232, PHE149, PHE97, PRO193, THR162, THR195, TRP160, TRP222, TYR158. Softened potentials were used with a tolerance of 1 and a strength 0.9. A search space volume of 17 Å radius was used centered around an averaged position of known InhA ligands after structural alignment of proteins. The structural NAD molecule was treated as a cofactor in calculations and was set as NAD+ with partial negative charges on phosphates and positive charge on the aromatic nicotinamide group. Clustering of poses (tabu clustering) was set with an RMSD threshold of 1.9 Å. A simple template (pharmacophoric profile) was used with a strength of -500 and a grid resolution of 0.4 Å; other similarity measure parameters were let at their default values. This pharmacophoric profile uses two atoms: the oxygen of alcohol group from ligand JPL (3FNG PDB structure [27]) and the oxygen from the keto group of GEQ (1P44 PDB structure [13]). These atoms are representative of conserved hydrogen bond (donor/acceptors) positions involving oxygen atoms along InhA ligands.

In the case of the OPT (differential evolution algorithm) protocol, the docking process used 10,000 iteration steps and a grid resolution of 0.3 Å, along 40 independent runs. Internal parameters (population size, crossover rate, scaling factor …) of the algorithm were let as default. A final minimization (per run) was parameterized using 4000 steps for lateral chains and 2000 steps for protein backbone preceded by minimization and optimization (hydrogen bonds) of ligands. The same parameters were applied to the MSE (Simplex evolution algorithm) protocol, with 40 independent runs, and the internal parameters (population size, number of iterations, energy threshold …) of the algorithm were set as default. These protocols generally perform well with InhA structures, and the typical RMSDs for reference ligands (i.e., TCL from 1P45 or JPL from 3FNG) were in the range of 0.5–1.0 Å for the best poses.

Pose topology analysis was carried out on the best MolDock and Rerank scores. Conformity criterion was set as the ability to reproduce a shared envelop of known ligand and typical interaction network: π-π or π-Alkyl interactions with the nicotinamide group of NAD; hydrogen bonding with TYR158; and hydrogen or electrostatic interactions with cofactor (OH from ribose, amido group, phosphates, charged nitrogen of pyridinium). In the context of consensus docking, if one or more poses combine the best values for each scoring scheme (Moldock and Rerank), they are described as strong poses.

### 3.4. Evaluation of InhA Enzyme Inhibition

Kinetic assays were performed using *trans*-2-dodecenoyl-coenzyme A (DDCoA) and His6-InhA as previously described [9,33]. Briefly, reactions were performed at 25 °C in 30 mM PIPES, 150 mM NaCl, pH 6.8, containing 250 μM of the NADH cofactor, 50 μM of the DDCoA substrate, and the tested compound at different concentrations. Reactions were initiated by addition of InhA (50 nM final) and NADH oxidation followed at 340 nm. The inhibitory activity of each compound was expressed as the percentage of inhibition of the InhA activity (initial velocity of the reaction) with respect to the control reaction without inhibitor. Inhibition = ((slope InhA alone − slope with compound)/slope InhA alone) × 100. All activity assays were performed in duplicate or triplicate.

### 3.5. Minimal Inhibitory Concentration Determination

The drug susceptibility of MTB H37Rv strain was assessed using the resazurin microtiter assay (REMA) in 7H9 Middlebrook medium, including positive (Streptomycin) and negative (7H9 medium w/o bacteria) growth controls on each plate. Serial 2-fold dilutions of the compounds were made in a 96-well black plate (Fluoronuc, Thermo Fisher Scientific), followed by the addition of bacterial cultures in the log phase. After 7 days of incubation at 37 °C, 10 μL of resazurin (0.025% *w*/*v*) was added to each well, and fluorescence was measured after an additional overnight incubation using a Fluoroskan Microplate Fluorometer (Thermo Fisher Scientific, Waltham, MA, USA; excitation = 544 nm; emission = 590 nm). Bacterial viability was determined as a percentage of resazurin turnover in the absence of the compound (internal negative control). Experiments were conducted in duplicate, and MIC90 values were obtained [34]. 

## 4. Conclusions

In summary, a new series of imidazoquinoline compounds designed as potential InhA inhibitors were synthesized in good yields through a three-step procedure. These compounds were evaluated as possible inhibitors of the InhA enzyme, but disappointingly were found to weakly inhibit InhA, with compound 4n showing the best, but moderate, activity. Docking studies showed a possible interaction mode in the protein substrate binding site. Secondly, being isosteric analogs of triazolophthalazine derivatives and for their resemblance for MmpL3 inhibitors, they were evaluated as inhibitors of the MTB strain. But these molecules showed no inhibitory activity. The introduction of various substituents from aryl to alkyl on the amide failed to promote activities against mycobacteria. Other isosteric compounds are currently being synthesized for evaluation against MTB. 

## Figures and Tables

**Table 1:** Inhibitory activity of imidazoquinoline compounds against InhA enzyme.

Cpd	% Inhibition at 50 μM	Purity (%) by LC-MS
**2a**	26	ND ^a^
**2b**	17	ND ^a^
**4a**	40	>99
**4b**	29	>99
**4c**	16	98
**4d**	0	>99
**4e**	23	>99
**4f**	10	>99
**4g**	19	>99
**4h**	4	97
**4i**	9	94
**4j**	31	>99
**4k**	13	>99
**4l**	7	98
**4m**	8	97
**4n**	66 (24) ^b^	>99
**4o**	16	99
**4p**	15	98
**4q**	34	97
**4r**	49	>99
**4s**	33	97
**Triclosan**	98	---

a. ND for not determined; b. Compound tested at 5 µM.

## Data Availability

Data are contained within the article and Supplementary Materials.

## Supplementary Materials

The following supporting information can be downloaded at: https://www.mdpi.com/article/10.3390/molecules29133076/s1, (Inhibitory activities, ^1^H and ^13^C NMR spectra) [35,36,37].
