# Supplementary material for: Imidazoquinoline Derivatives as Potential Inhibitors of InhA Enzyme and Mycobacterium tuberculosis
Source: Molecules. 2024 Jun 27;29(13):3076. doi: 10.3390/molecules29133076 (PMC11243711; doi:10.3390/molecules29133076)
Supplement: Supplementary file 1 [file molecules-29-03076-s001.zip › molecules-3081219-supplementary.pdf]

SUPPLEMENTARY MATERIAL

Imidazoquinoline Derivatives as Potential Inhibitors of InhA Enzyme and *Mycobacterium tuberculosis*

Pascal Hoffmann [1], Joëlle Azéma-Despeyroux [1], Fernanda Goncalves [1], Alessandro Stamilla [2], Nathalie Saffon-Merceron [3], Frédéric Rodriguez [1], Giulia Degiacomi [2], Maria Rosalia Pasca [2] and Christian Lherbet [1,*]

[1] Laboratoire de Synthèse et Physico-Chimie de Molécules d'Intérêt Biologique (SPCMIB), UMR5068, CNRS, Université Paul Sabatier Toulouse III, 31062 Toulouse, France; pascal.hoffmann@univ-tlse3.fr (P.H.); joelle.azema-despeyroux@univ-tlse3.fr (J.A.-D.); fernanda.goncalves@cnrs.fr (F.G.); frederic.rodriguez@univ-tlse3.fr (F.R.)

[2] Department of Biology and Biotechnology "Lazzaro Spallanzani", University of Pavia, 27100 Pavia, Italy; alessandro.stamilla@unipv.it (A.S.); giulia.degiacomi@unipv.it (G.D.); mariarosalia.pasca@unipv.it (M.R.P.)

[3] Institut de Chimie de Toulouse, ICT-UAR2599, Université Paul Sabatier Toulouse III, 31062 Toulouse, France; nathalie.saffon@univ-tlse3.fr

* Correspondence: christian.lherbet@univ-tlse3.fr

	Pages
Inhibitory activity of imidazoquinoline derivatives toward Mtb H37Rv strain	2
^{1}H NMR, ^{13}C NMR spectra and HRMS for all the compounds	3-40
Selected crystallographic data for compounds **4g** and **4h**	41

Table S1. Inhibitory activity of imidazoquinoline derivatives toward Mtb H37Rv strain

CMPs	MIC (μg/ml)
**2a**	**≥40**
**4a**	**≥40**
**4b**	**≥40**
**4c**	**≥40**
**4d**	**>40**
**4e**	**≥40**
**4f**	**>40**
**4g**	**≥40**
**4h**	**≥40**
**4i**	**≥40**
**4j**	**≥40**
**4k**	**≥40**
**4l**	**≥40**
**4m**	**≥40**
**4n**	**≥40**
**4o**	**≥40**
**4p**	**≥40**
**4q**	**≥40**
**4r**	**≥40**
**4s**	**≥40**
**Streptomycin**	**0,25**

Compound 4a

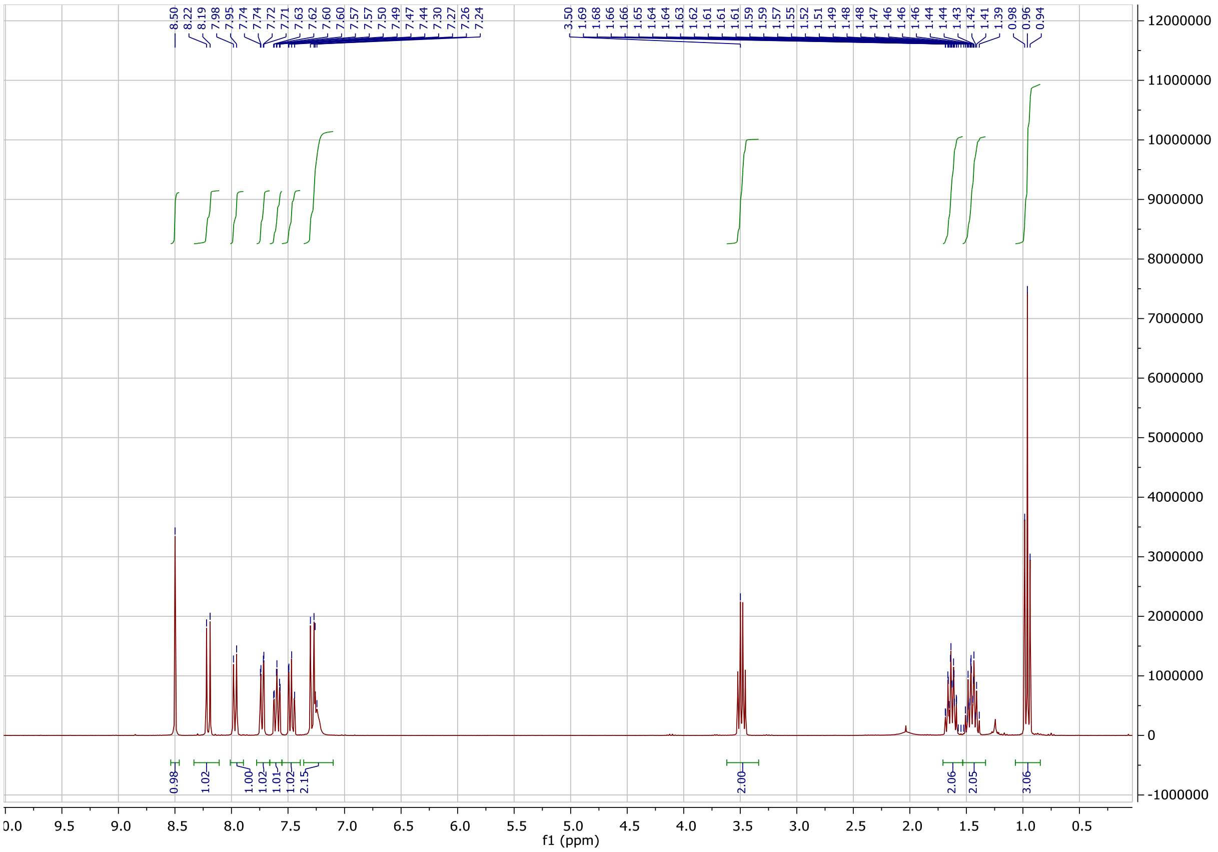

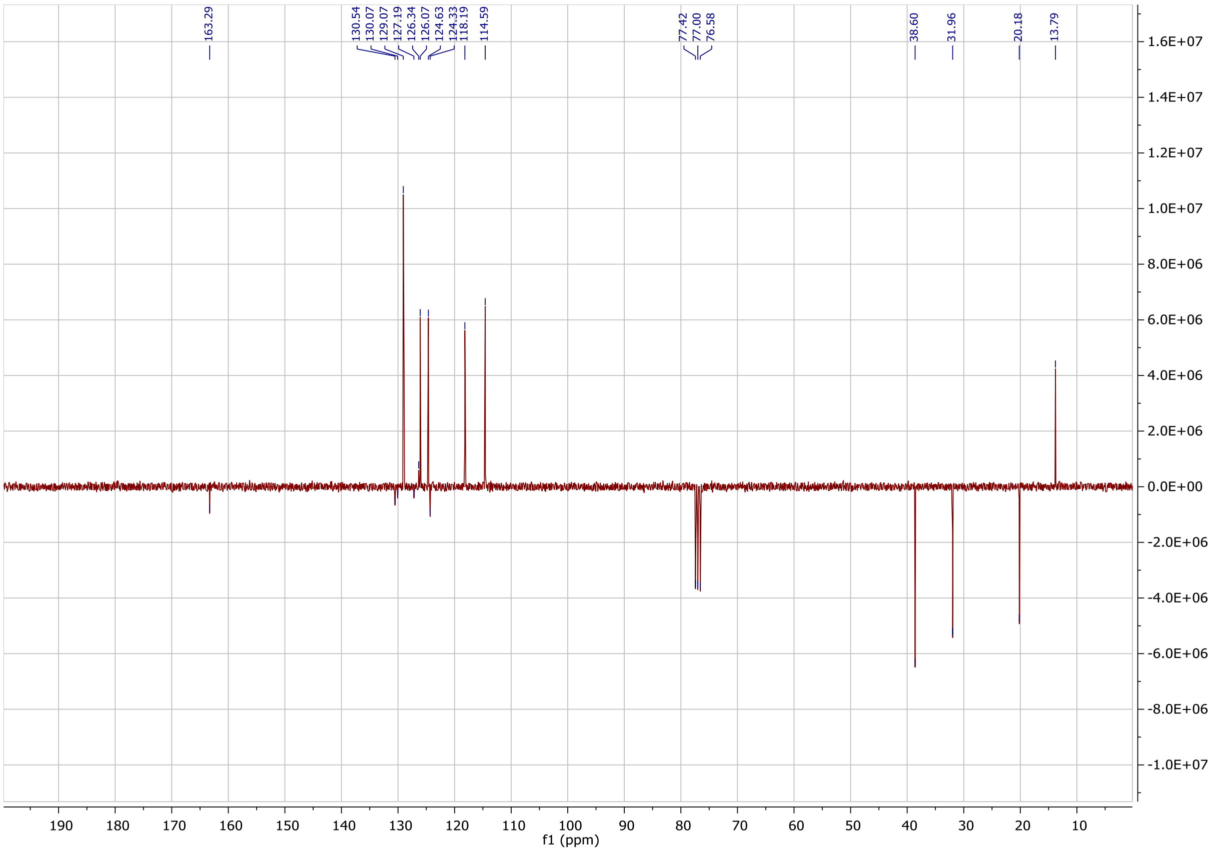

Single Mass Analysis
Tolerance = 5.0 PPM / DBE: min = -1.5, max = 50.0
Element prediction: Off

Monoisotopic Mass, Odd and Even Electron Ions
326 formula(e) evaluated with 2 results within limits (all results (up to 1000) for each mass)
Elements Used:
C: 0-100 H: 0-100 N: 0-10 O: 0-10

DCI-CH4 GCT Premier CAB109 27-Jun-201910:54:17
20190627-CL9-22 16 (0.267) Cm (15:26-102:107x5.000) TOF MS CI+
5.09e+004

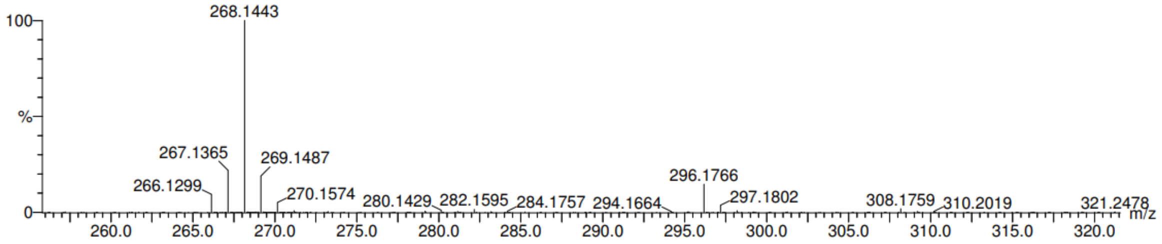

Minimum:				-1.5		
Maximum:		1.4	5.0	50.0		
Mass	Calc. Mass	mDa	PPM	DBE	i-FIT	Formula
268.1443	268.1450	-0.7	-2.6	9.5	464.1	C16 H18 N3 O
	268.1436	0.7	2.6	10.0	602.9	C14 H16 N6

Compound 4b

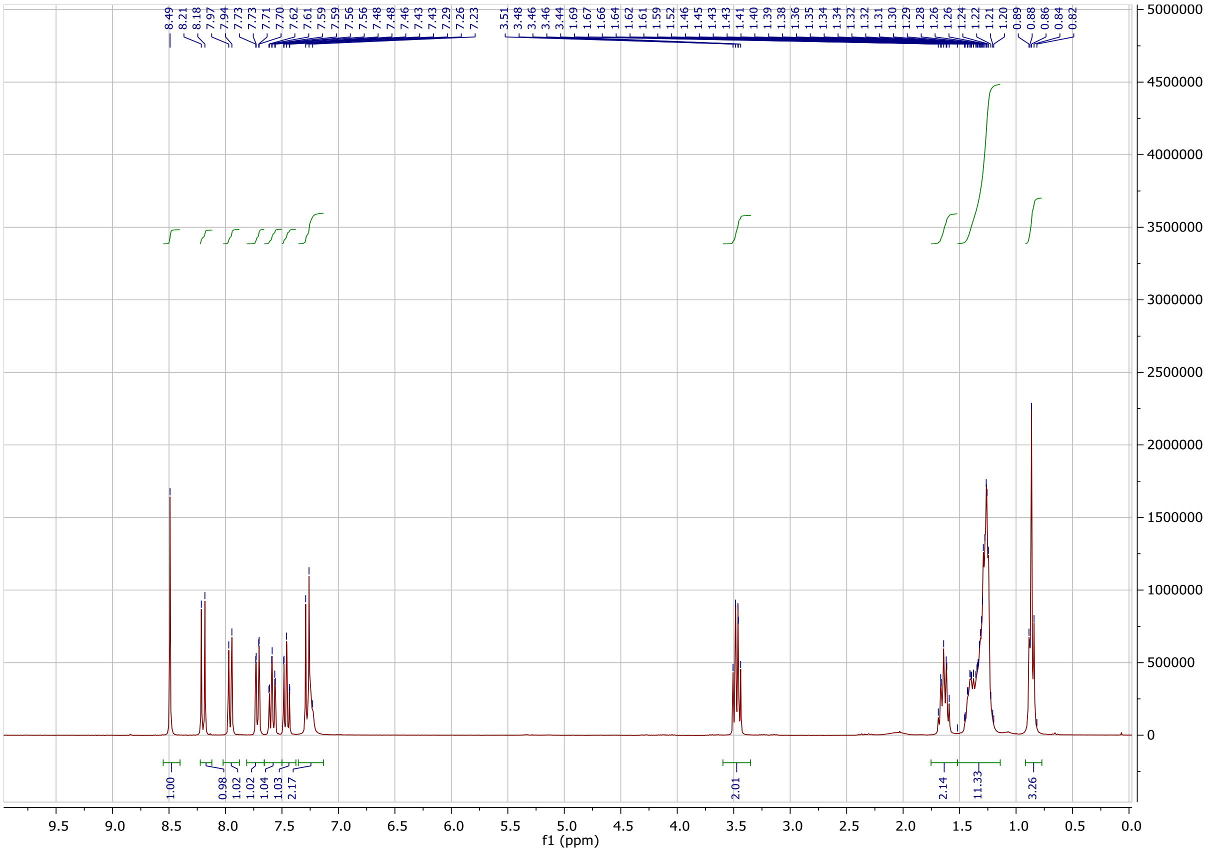

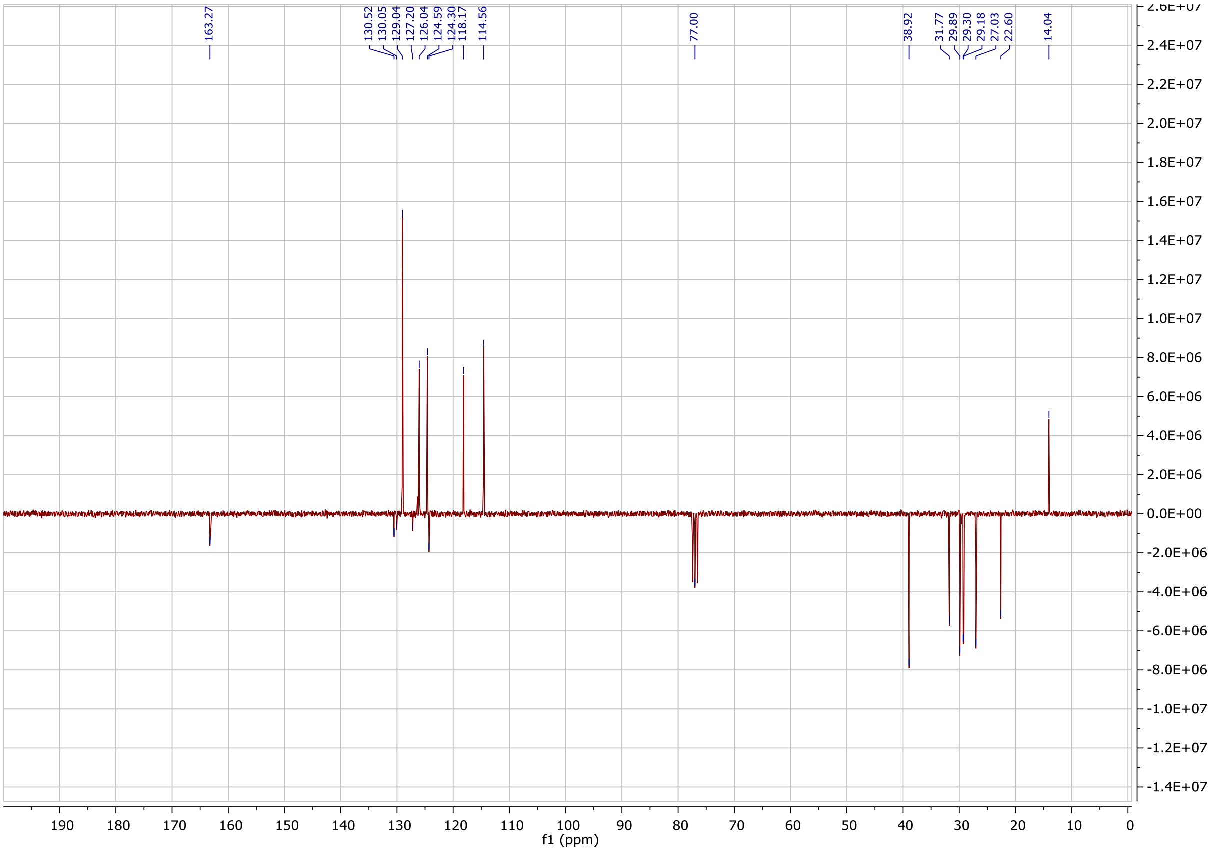

Single Mass Analysis
Tolerance = 5.0 PPM / DBE: min = -1.5, max = 50.0
Element prediction: Off

Monoisotopic Mass, Odd and Even Electron Ions
150 formula(e) evaluated with 2 results within limits (all results (up to 1000) for each mass)
Elements Used:
C: 0-100 H: 0-100 N: 0-5 O: 0-5

DCI-CH4 GCT Premier CAB109 03-Jun-201910:27:55
20190603-CL9-20 19 (0.317) Cm (17:21-93:95x5.000) TOF MS CI+
5.45e+004

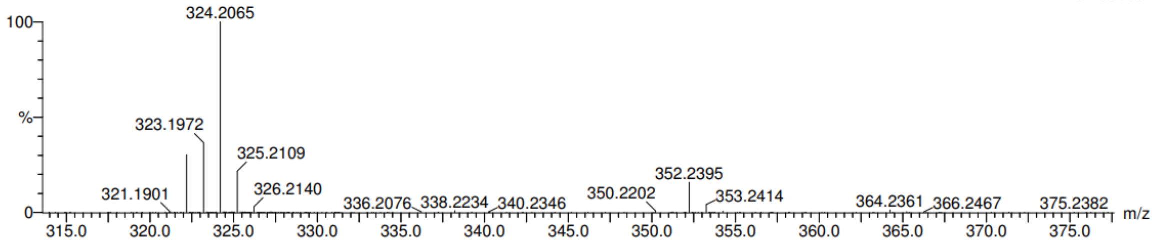

Minimum:				-1.5		
Maximum:		1.4	5.0	50.0		
Mass	Calc. Mass	mDa	PPM	DBE	i-FIT	Formula
324.2065	324.2049	1.6	4.9	5.0	24.1	C17 H28 N2 O4
	324.2076	-1.1	-3.4	9.5	59.8	C20 H26 N3 O

Compound 4c

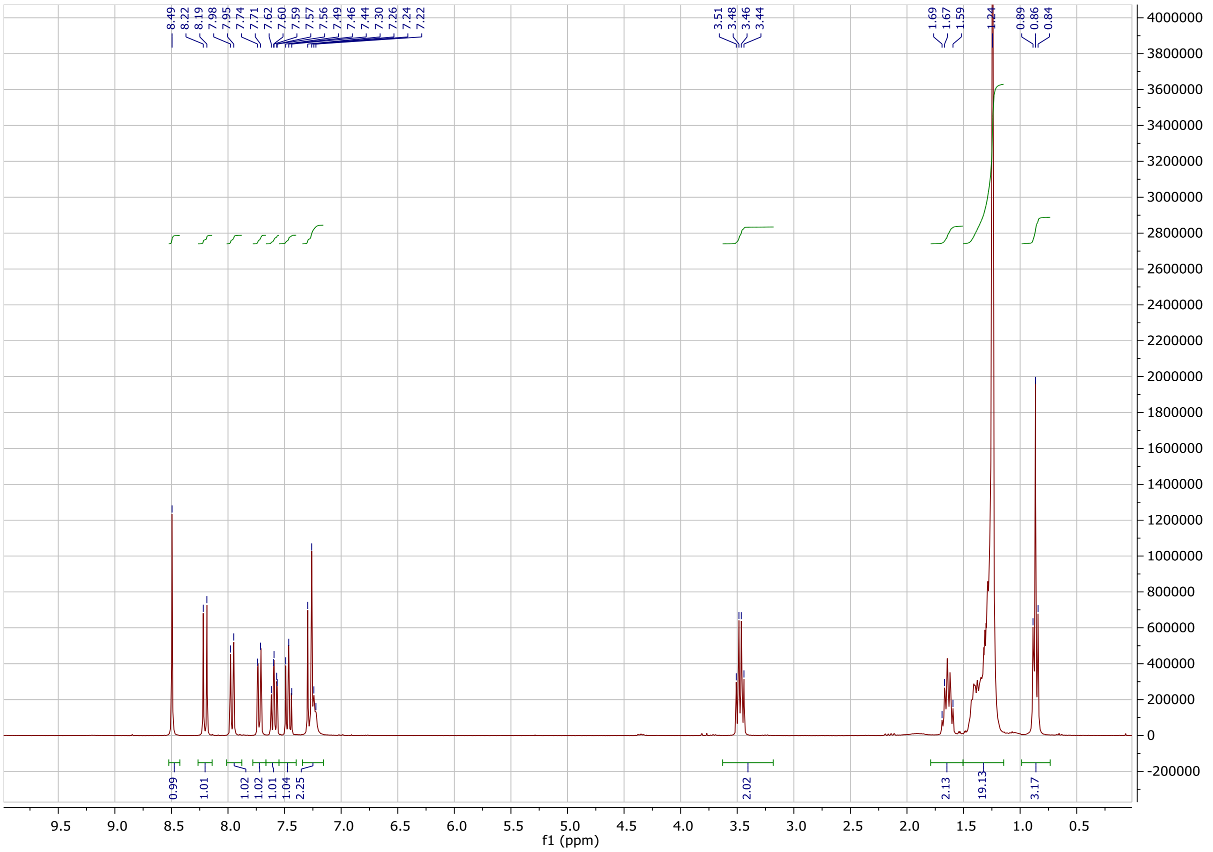
8.49
8.22
8.19
7.98
7.95
7.74
7.71
7.62
7.60
7.59
7.57
7.56
7.49
7.46
7.44
7.30
7.26
7.24
7.22
3.51
3.48
3.46
3.44
1.69
1.67
1.59
1.24
0.89
0.86
0.84
0.99
1.01
1.02
1.02
1.01
1.04
2.25
2.02
2.13
19.13
3.17
f1 (ppm)

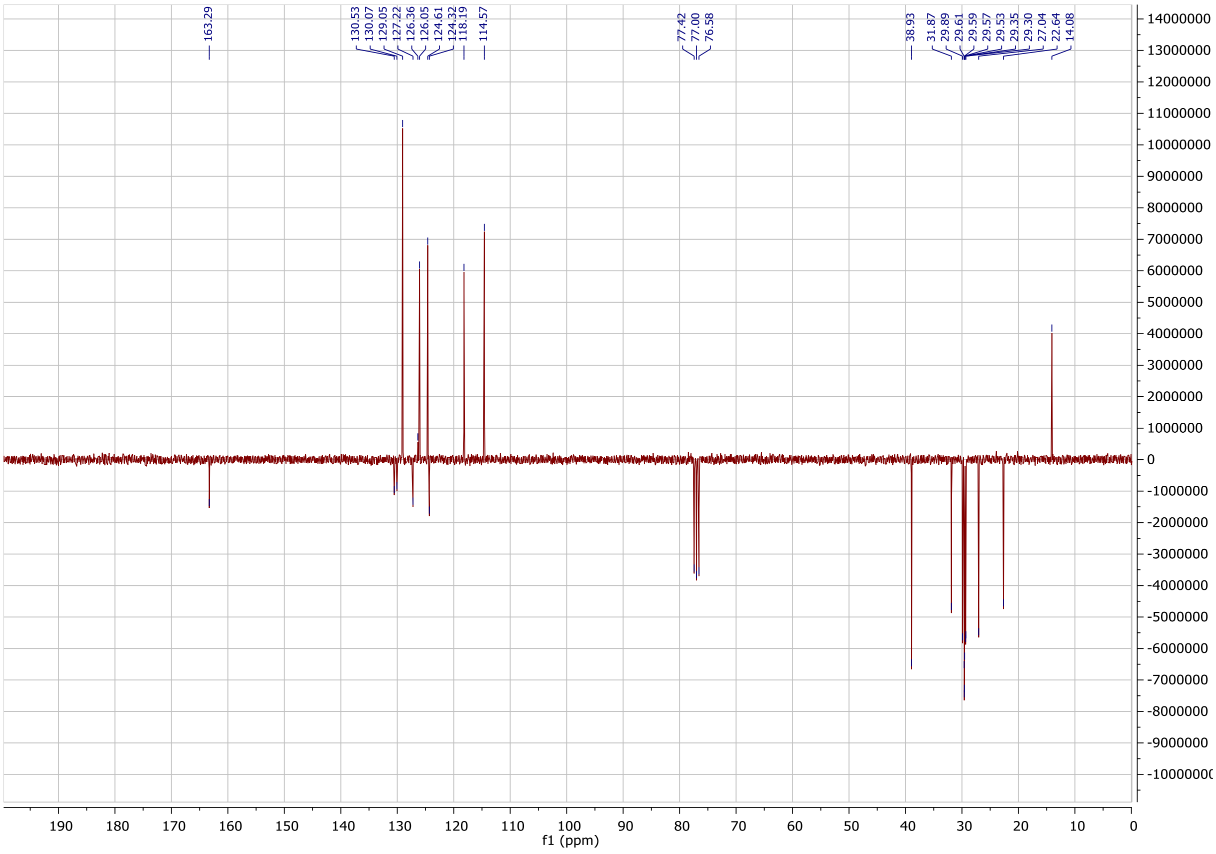
163.29
130.53
130.07
129.05
127.22
126.36
126.05
124.61
124.32
118.19
114.57
77.42
77.00
76.58
38.93
31.87
29.89
29.61
29.59
29.57
29.53
29.35
29.30
27.04
22.64
14.08
f1 (ppm)

Single Mass Analysis
Tolerance = 5.0 PPM / DBE: min = -1.5, max = 80.0
Element prediction: Off

Monoisotopic Mass, Odd and Even Electron Ions
174 formula(e) evaluated with 2 results within limits (all results (up to 1000) for each mass)
Elements Used:
C: 0-100 H: 0-100 N: 0-5 O: 0-5

DCI-CH4
20220708-CL9-48 65 (1.084) Cm (61:70-1:7x5.000)

GCT Premier CAB109

08-Jul-202210:11:06
TOF MS CI+
7.29e+004

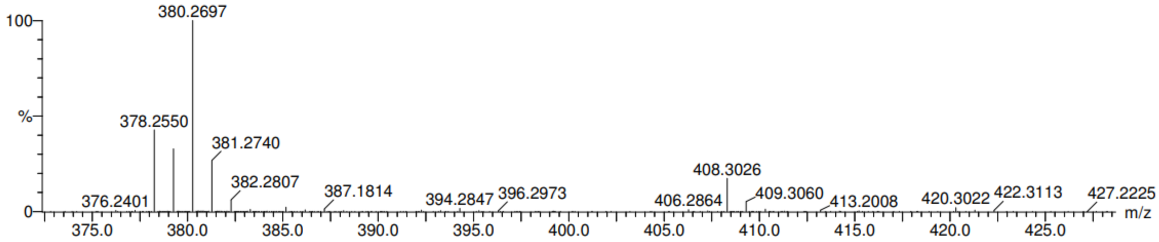

Mass	Calc. Mass	mDa	PPM	DBE	i-FIT	Formula
Minimum:				-1.5		
Maximum:		1.5	5.0	80.0		
380.2697	380.2702	-0.5	-1.3	9.5	265.4	C24 H34 N3 O
	380.2715	-1.8	-4.7	9.0	224.5	C26 H36 O2

Compound 4d

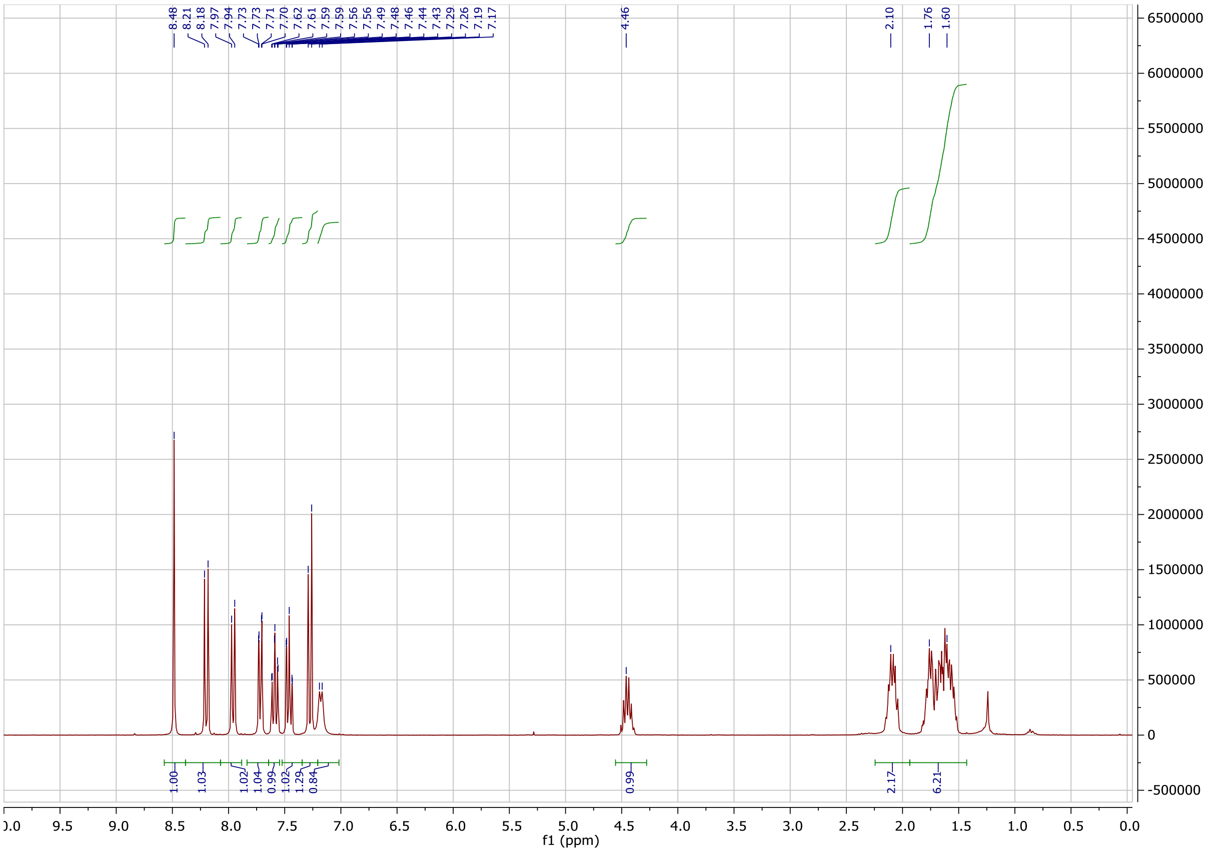
8.48
8.21
8.18
7.97
7.94
7.73
7.73
7.71
7.70
7.62
7.61
7.59
7.59
7.56
7.56
7.49
7.48
7.46
7.44
7.43
7.29
7.26
7.19
7.17
4.46
2.10
1.76
1.60
1.00
1.03
1.02
1.04
0.99
1.02
1.29
0.84
0.99
2.17
6.21
f1 (ppm)

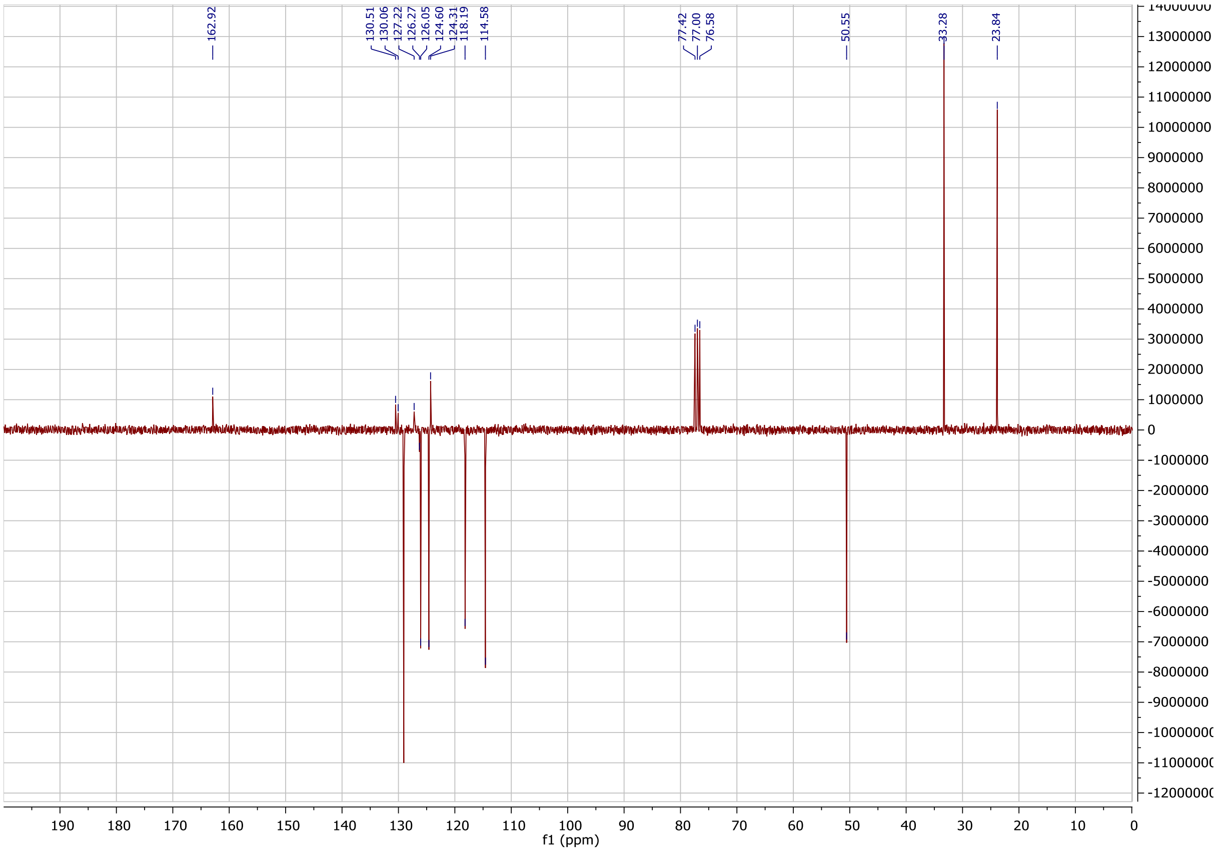
162.92
130.51
130.06
127.22
126.27
126.05
124.60
124.31
118.19
114.58
77.42
77.00
76.58
50.55
33.28
23.84
f1 (ppm)

Single Mass Analysis
Tolerance = 5.0 PPM / DBE: min = -1.5, max = 50.0
Element prediction: Off

Monoisotopic Mass, Odd and Even Electron Ions
346 formula(e) evaluated with 2 results within limits (all results (up to 1000) for each mass)
Elements Used:
C: 0-100 H: 0-100 N: 0-10 O: 0-10

DCI-CH4 GCT Premier CAB109 27-Jun-201910:44:43
20190627-CL9-23 40 (0.667) Cm (35:45-132:137x5.000) TOF MS CI+
1.07e+005

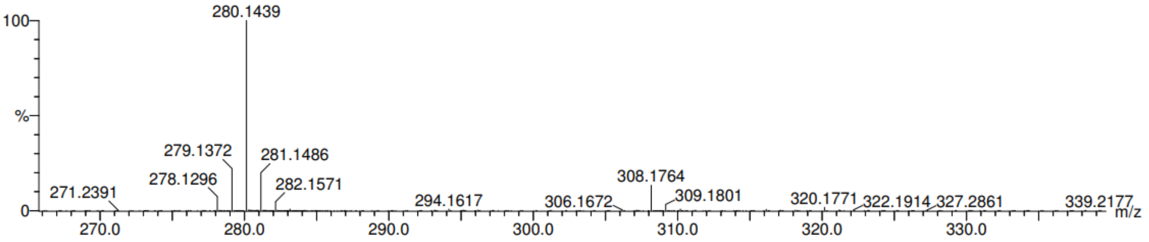

Minimum:				-1.5		
Maximum:		1.4	5.0	50.0		
Mass	Calc. Mass	mDa	PPM	DBE	i-FIT	Formula
280.1439	280.1450	-1.1	-3.9	10.5	661.7	C17 H18 N3 O
	280.1436	0.3	1.1	11.0	914.0	C15 H16 N6

Compound 4e

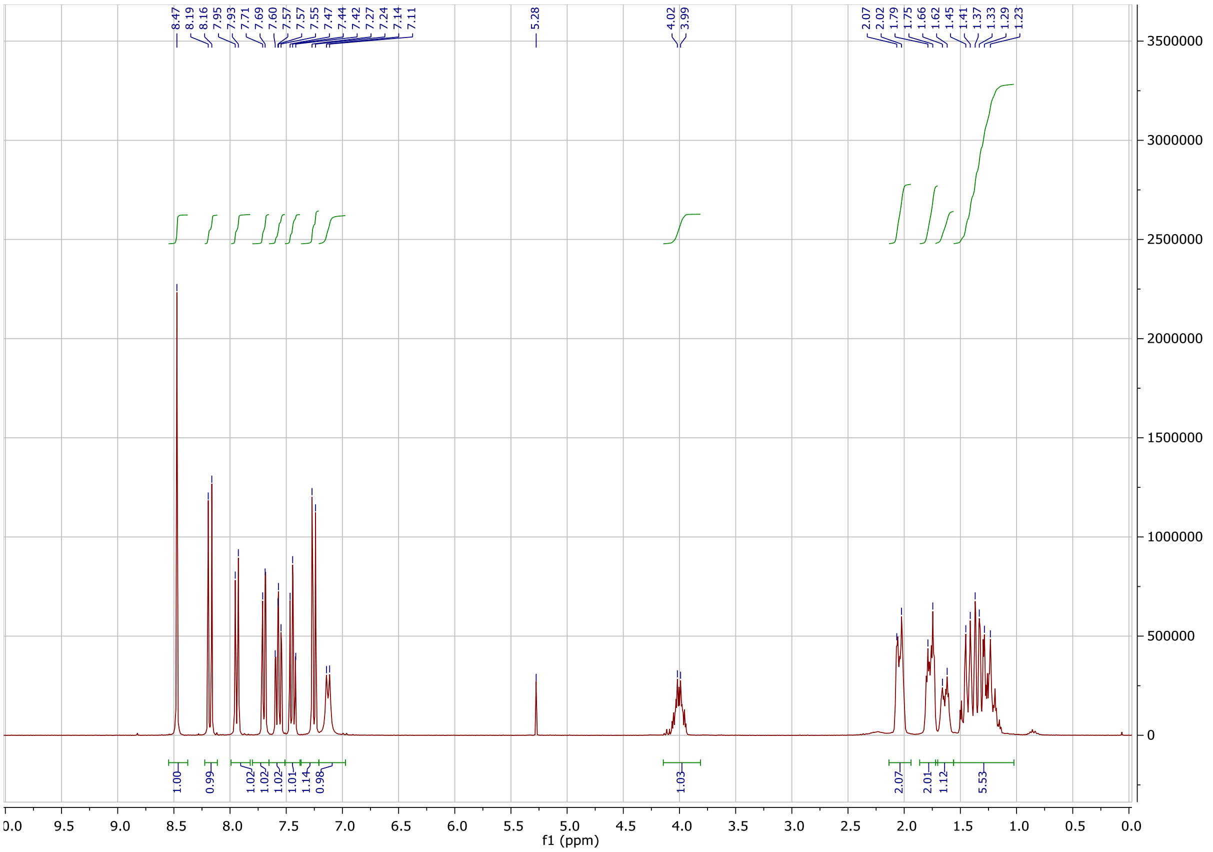
8.47
8.19
8.16
7.95
7.93
7.71
7.69
7.60
7.57
7.57
7.55
7.47
7.44
7.42
7.27
7.24
7.14
7.11
5.28
4.02
3.99
2.07
2.02
1.79
1.75
1.66
1.62
1.45
1.41
1.37
1.33
1.29
1.23
1.00
0.99
1.02
1.02
1.02
1.01
1.14
0.98
1.03
2.07
2.01
1.12
5.53
f1 (ppm)

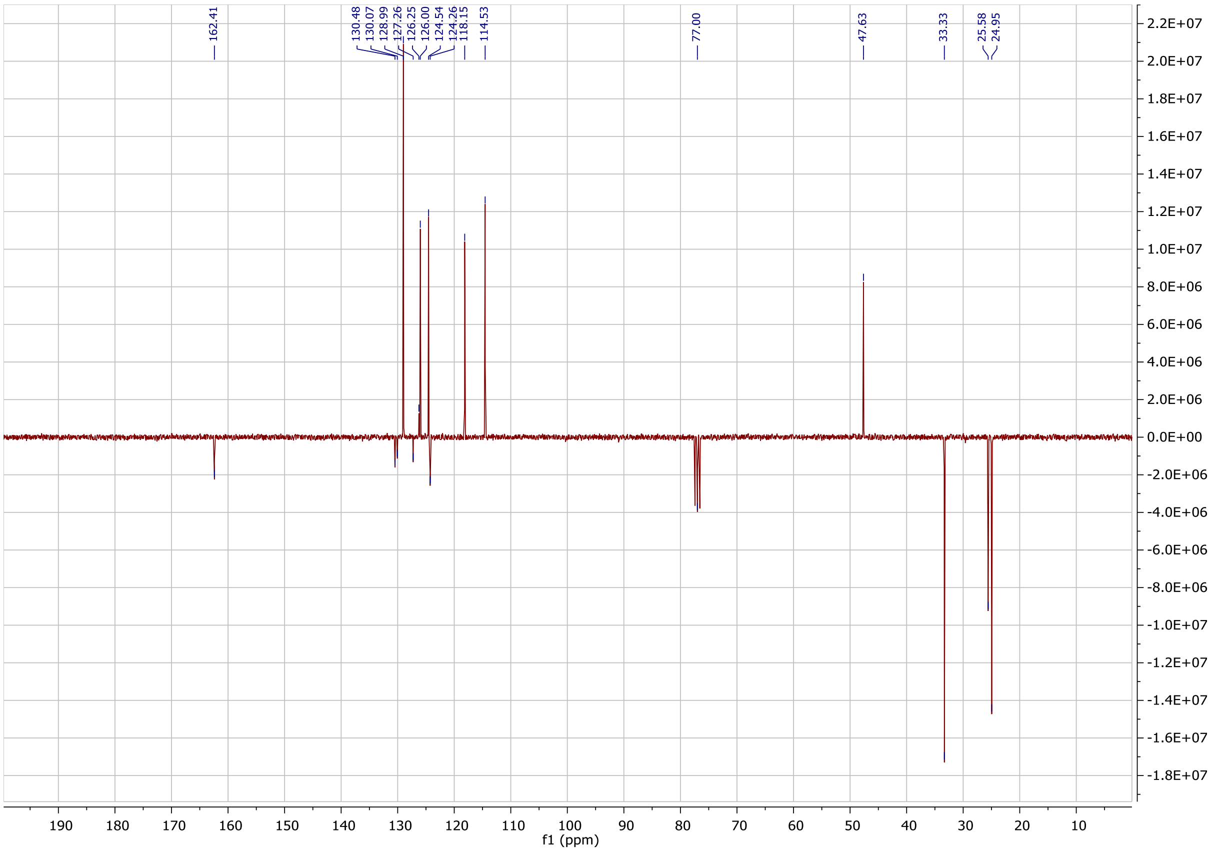
162.41
130.48
130.07
128.99
127.26
126.25
126.00
124.54
124.26
118.15
114.53
77.00
47.63
33.33
25.58
24.95
f1 (ppm)

Single Mass Analysis

Tolerance = 5.0 PPM / DBE: min = -1.5, max = 50.0
Element prediction: Off

Monoisotopic Mass, Odd and Even Electron Ions
136 formula(e) evaluated with 1 results within limits (all results (up to 1000) for each mass)
Elements Used:
C: 0-100 H: 0-100 N: 0-5 O: 0-5

DCI-CH4 GCT Premier CAB109 03-Jun-201910:21:06
20190603-CL9-21 16 (0.267) Cm (13:21-81:84x5.000) TOF MS CI+
2.52e+004

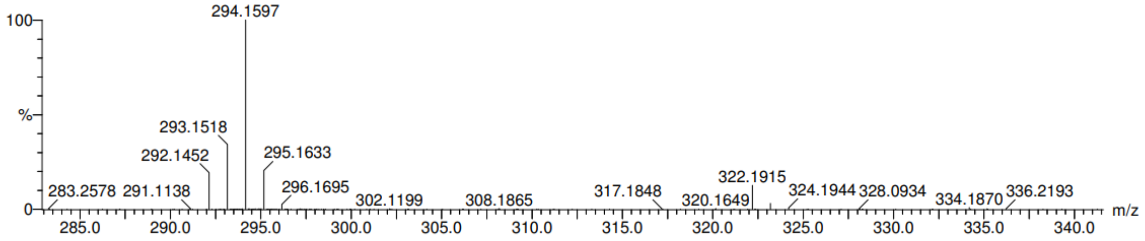

Minimum:				-1.5		
Maximum:		1.4	5.0	50.0		
Mass	Calc. Mass	mDa	PPM	DBE	i-FIT	Formula
294.1597	294.1606	-0.9	-3.1	10.5	8.1	C18 H20 N3 O

Compound 4f

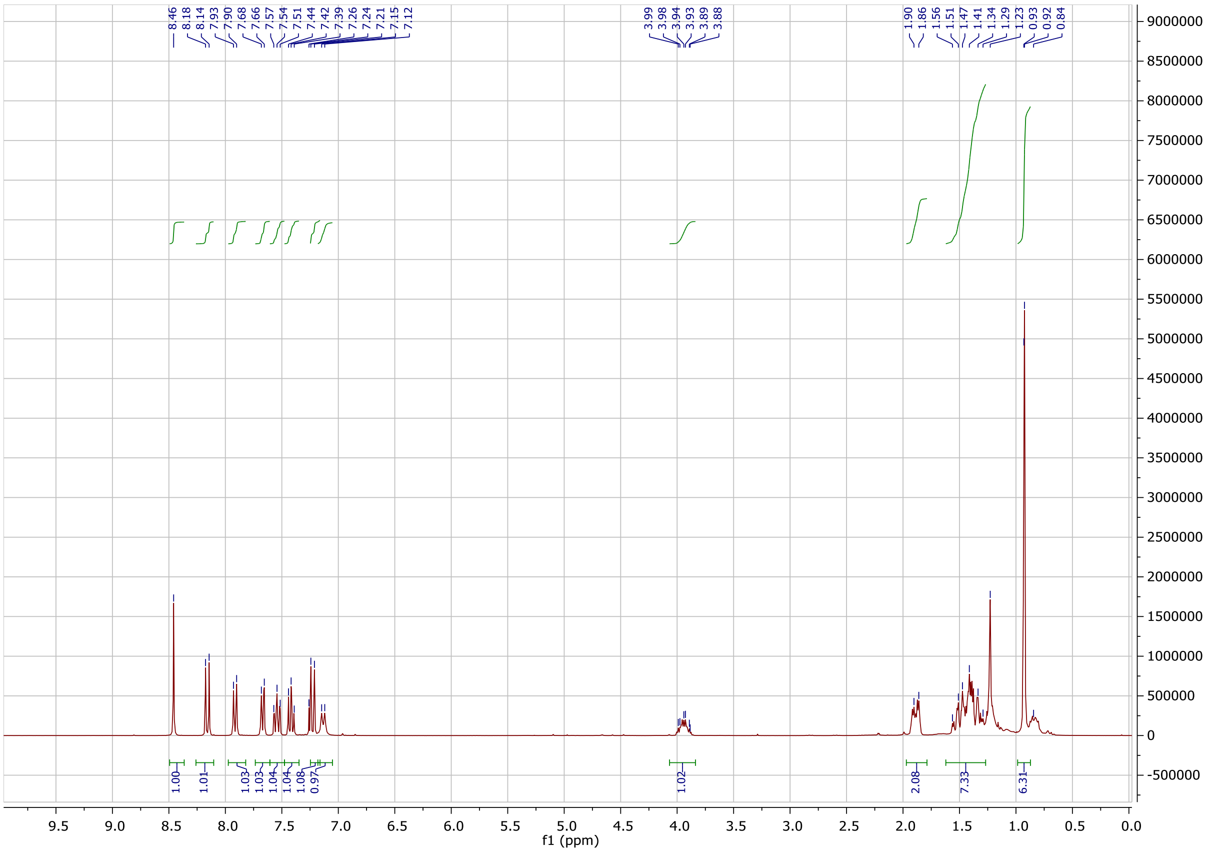

8.46 8.18 8.14 7.93 7.90 7.68 7.66 7.57 7.54 7.51 7.44 7.42 7.39 7.26 7.24 7.21 7.15 7.12
3.99 3.98 3.94 3.93 3.89 3.88
1.90 1.86 1.56 1.51 1.47 1.41 1.34 1.29 1.23 0.93 0.92 0.84
1.00 1.01 1.03 1.03 1.04 1.04 1.08 0.97 1.02 2.08 7.33 6.31
f1 (ppm)

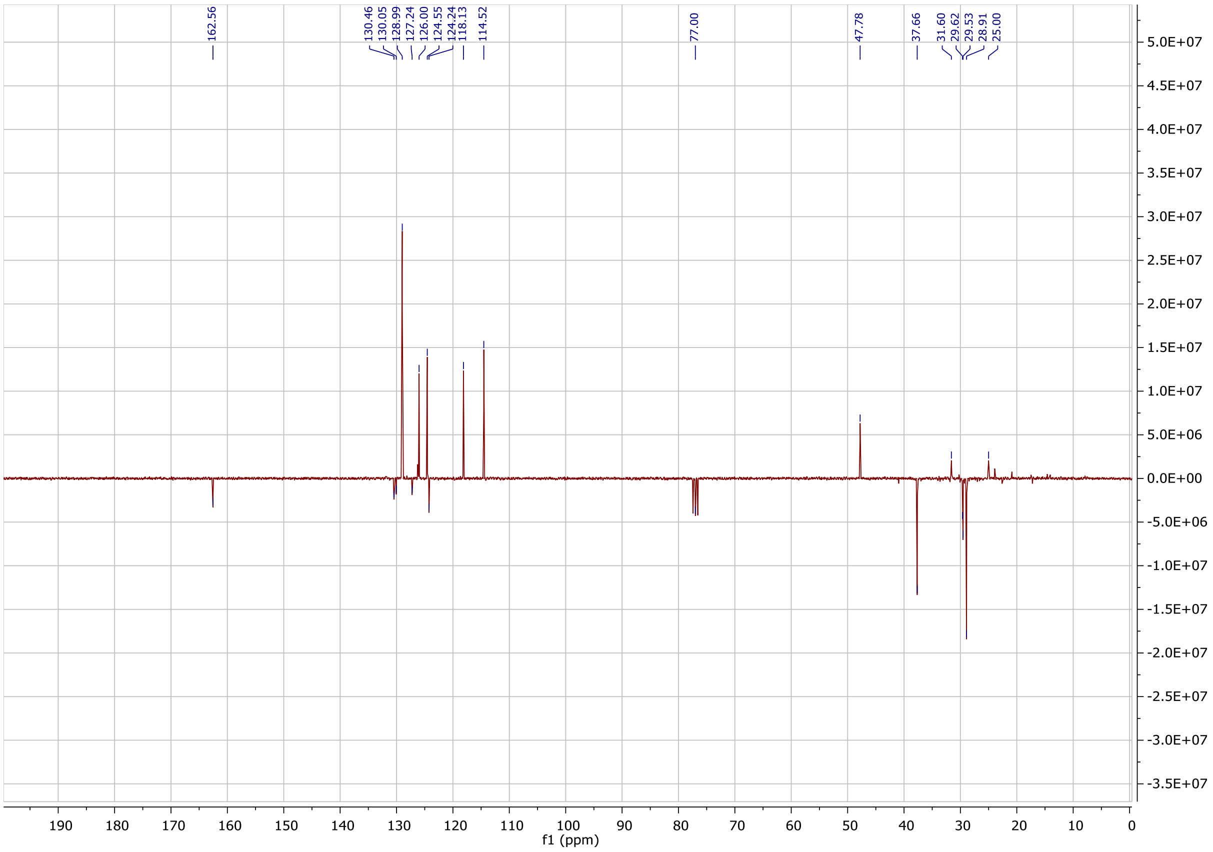

162.56
130.46 130.05 128.99 127.24 126.00 124.55 124.24 118.13 114.52
77.00
47.78
37.66 31.60 29.62 29.53 28.91 25.00
f1 (ppm)

Single Mass Analysis
Tolerance = 5.0 PPM / DBE: min = -1.5, max = 80.0
Element prediction: Off

Monoisotopic Mass, Odd and Even Electron Ions
213 formula(e) evaluated with 3 results within limits (all results (up to 1000) for each mass)
Elements Used:
C: 0-100 H: 0-100 N: 0-8 O: 0-5

DCI-CH4 GCT Premier CAB109 07-Jul-202211:32:38
20220707-CL9-62 51 (0.850) Cm (45:51-126:131x5.000) TOF MS CI+
4.81e+004

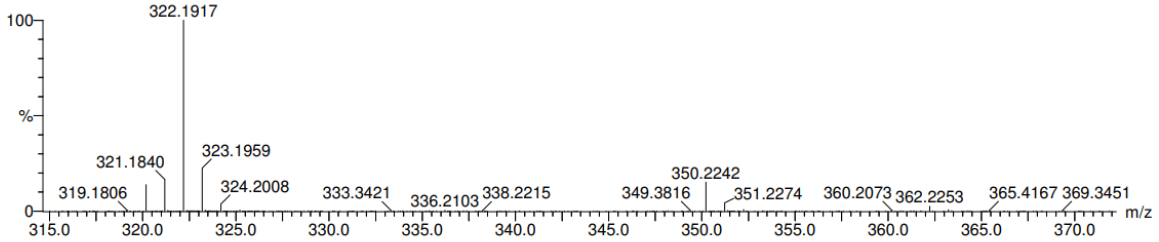

Minimum:				-1.5		
Maximum:		1.5	5.0	80.0		
Mass	Calc. Mass	mDa	PPM	DBE	i-FIT	Formula
322.1917	322.1919	-0.2	-0.6	10.5	40.7	C20 H24 N3 O
	322.1906	1.1	3.4	11.0	75.9	C18 H22 N6
	322.1933	-1.6	-5.0	10.0	61.5	C22 H26 O2

Compound 4g

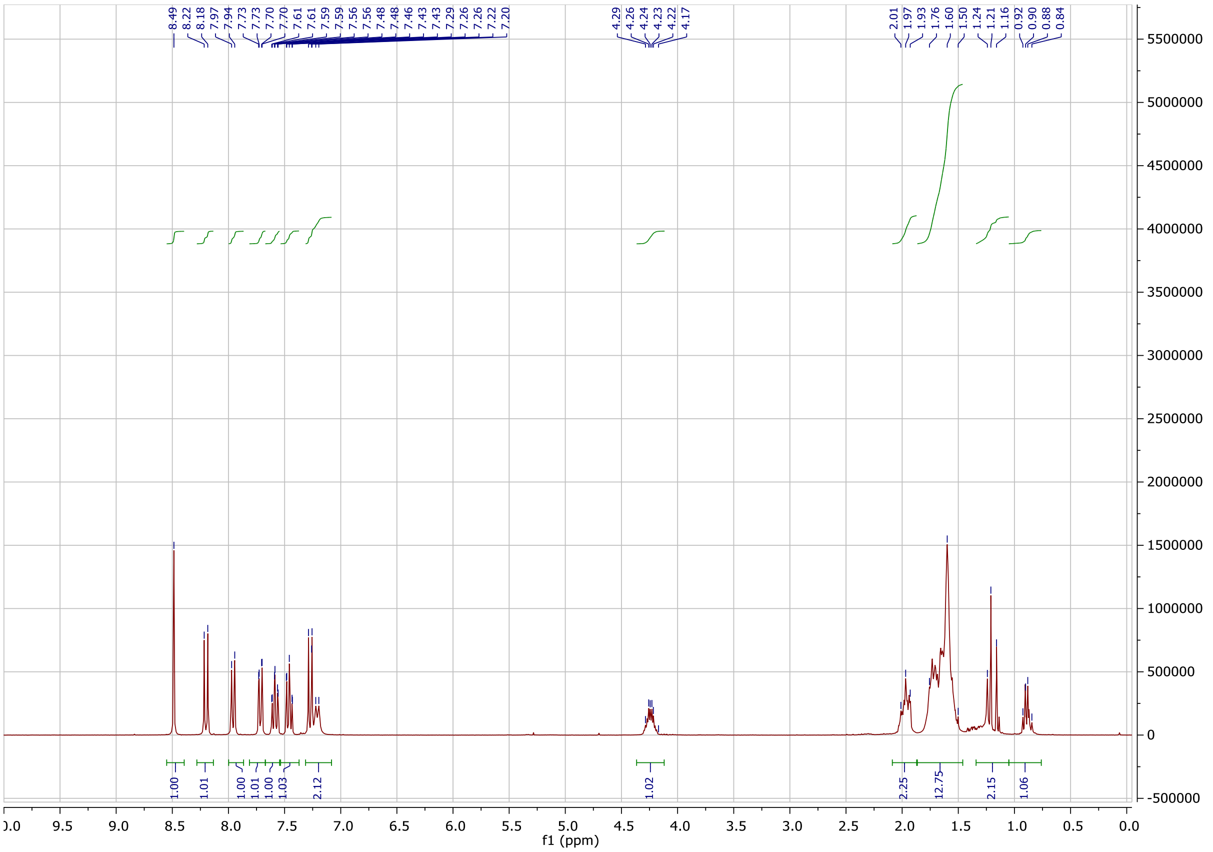
8.49
8.22
8.18
7.97
7.94
7.73
7.73
7.70
7.70
7.61
7.61
7.59
7.59
7.56
7.56
7.48
7.48
7.46
7.43
7.43
7.29
7.26
7.26
7.22
7.20
4.29
4.26
4.24
4.23
4.22
4.17
2.01
1.97
1.93
1.76
1.60
1.50
1.24
1.21
1.16
0.92
0.90
0.88
0.84
1.00
1.01
1.00
1.01
1.00
1.03
2.12
1.02
2.25
12.75
2.15
1.06
f1 (ppm)

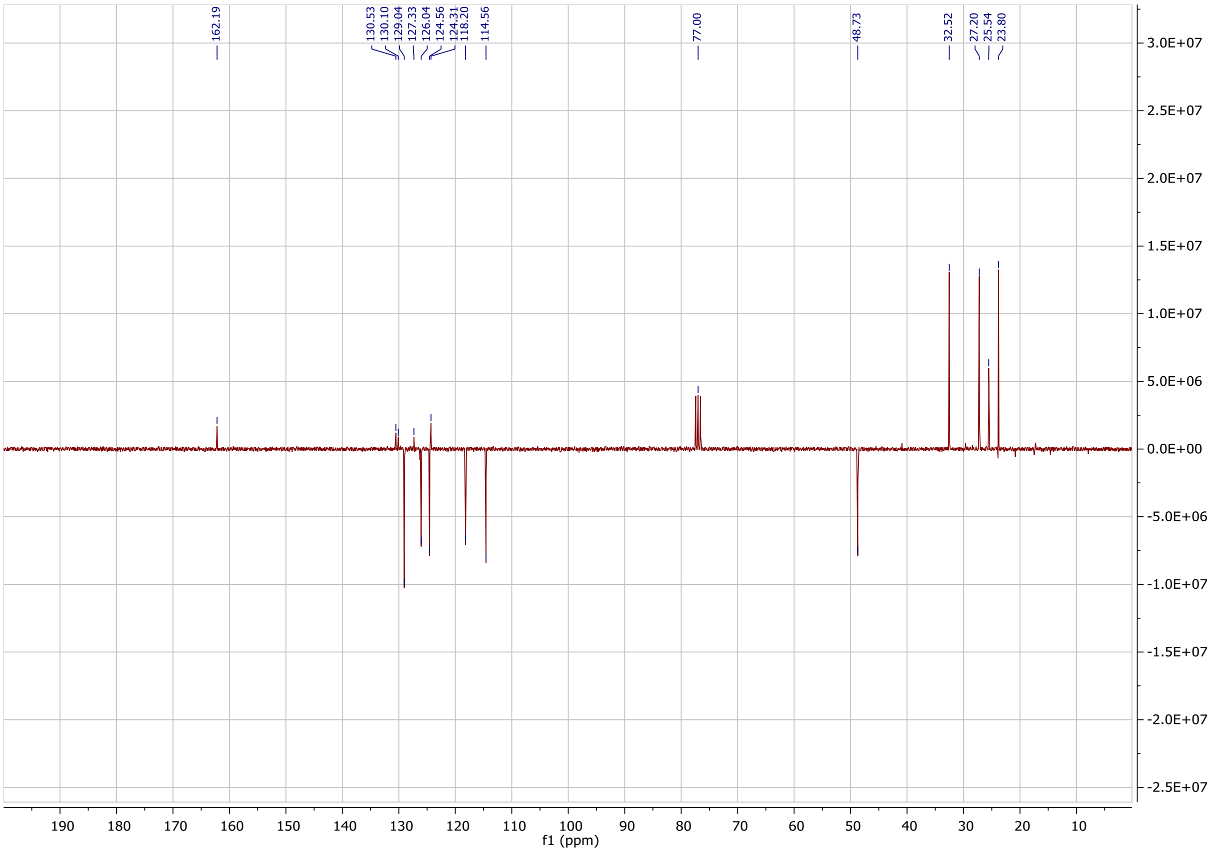
162.19
130.53
130.10
129.04
127.33
126.04
124.56
124.31
118.20
114.56
77.00
48.73
32.52
27.20
25.54
23.80
f1 (ppm)

Single Mass Analysis

Tolerance = 5.0 PPM / DBE: min = -1.5, max = 50.0
Element prediction: Off

Monoisotopic Mass, Odd and Even Electron Ions
148 formula(e) evaluated with 2 results within limits (all results (up to 1000) for each mass)
Elements Used:
C: 0-100 H: 0-100 N: 0-5 O: 0-5

DCI-CH4 GCT Premier CAB109 03-Jun-201909:55:56
20190603-CL9-14 19 (0.317) Cm (17:21-127:128x5.000) TOF MS CI+ 6.23e+004

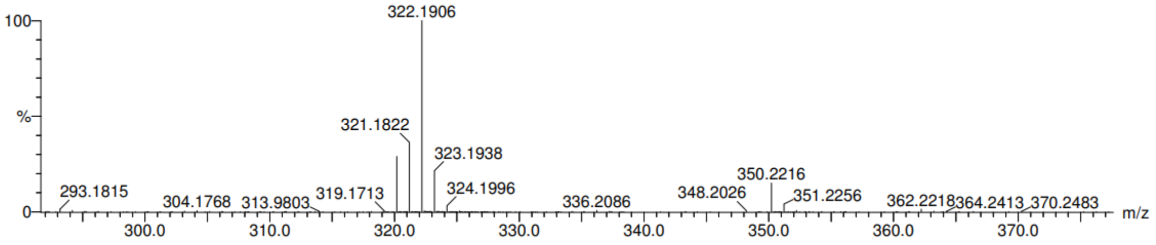

Minimum:				-1.5		
Maximum:		1.4	5.0	50.0		
Mass	Calc. Mass	mDa	PPM	DBE	i-FIT	Formula
322.1906	322.1893	1.3	4.0	6.0	24.9	C17 H26 N2 O4
	322.1919	-1.3	-4.0	10.5	75.5	C20 H24 N3 O

Compound 4h

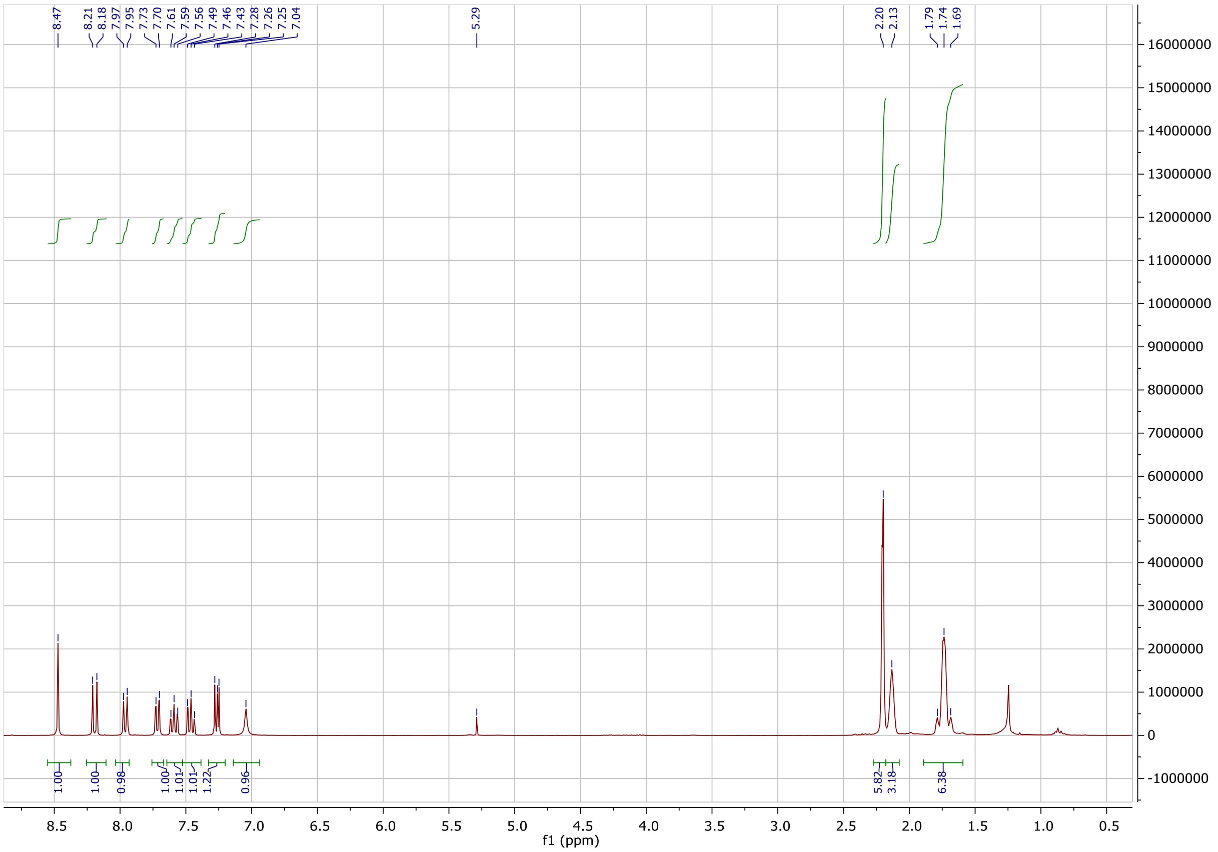

8.47
8.21
8.18
7.97
7.95
7.73
7.70
7.61
7.59
7.56
7.49
7.46
7.43
7.28
7.26
7.25
7.04
5.29
2.20
2.13
1.79
1.74
1.69
1.00
1.00
0.98
1.00
1.01
1.01
1.22
0.96
5.82
3.18
6.38
f1 (ppm)

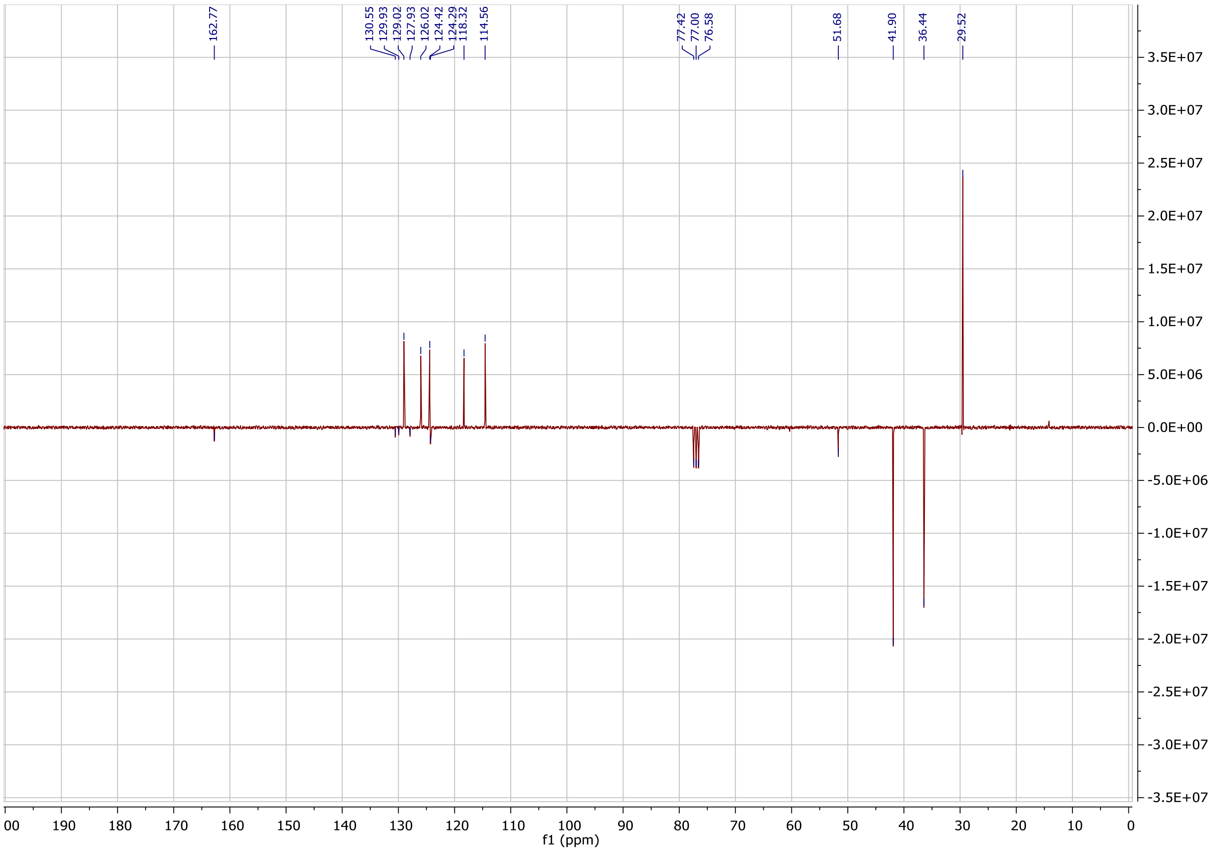

162.77
130.55
129.93
129.02
127.93
126.02
124.42
124.29
118.32
114.56
77.42
77.00
76.58
51.68
41.90
36.44
29.52
f1 (ppm)

Single Mass Analysis

Tolerance = 5.0 PPM / DBE: min = -1.5, max = 50.0
Element prediction: Off

Monoisotopic Mass, Odd and Even Electron Ions
156 formula(e) evaluated with 2 results within limits (all results (up to 1000) for each mass)
Elements Used:
C: 0-100 H: 0-100 N: 0-5 O: 0-5

DCI-CH4 GCT Premier CAB109 03-Jun-201910:05:03
20190603-CL9-15 57 (0.950) Cm (56:59-92:93x5.000) TOF MS CI+
4.43e+003

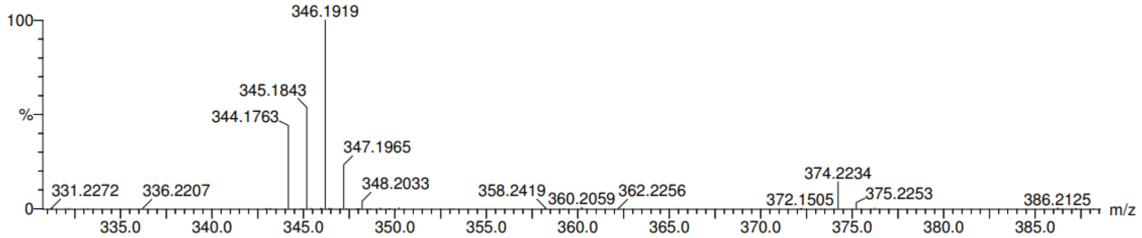

Minimum:				-1.5		
Maximum:		1.4	5.0	50.0		
Mass	Calc. Mass	mDa	PPM	DBE	i-FIT	Formula
346.1919	346.1919	0.0	0.0	12.5	7.1	C22 H24 N3 O
	346.1933	-1.4	-4.0	12.0	12.3	C24 H26 O2

Compound 4i

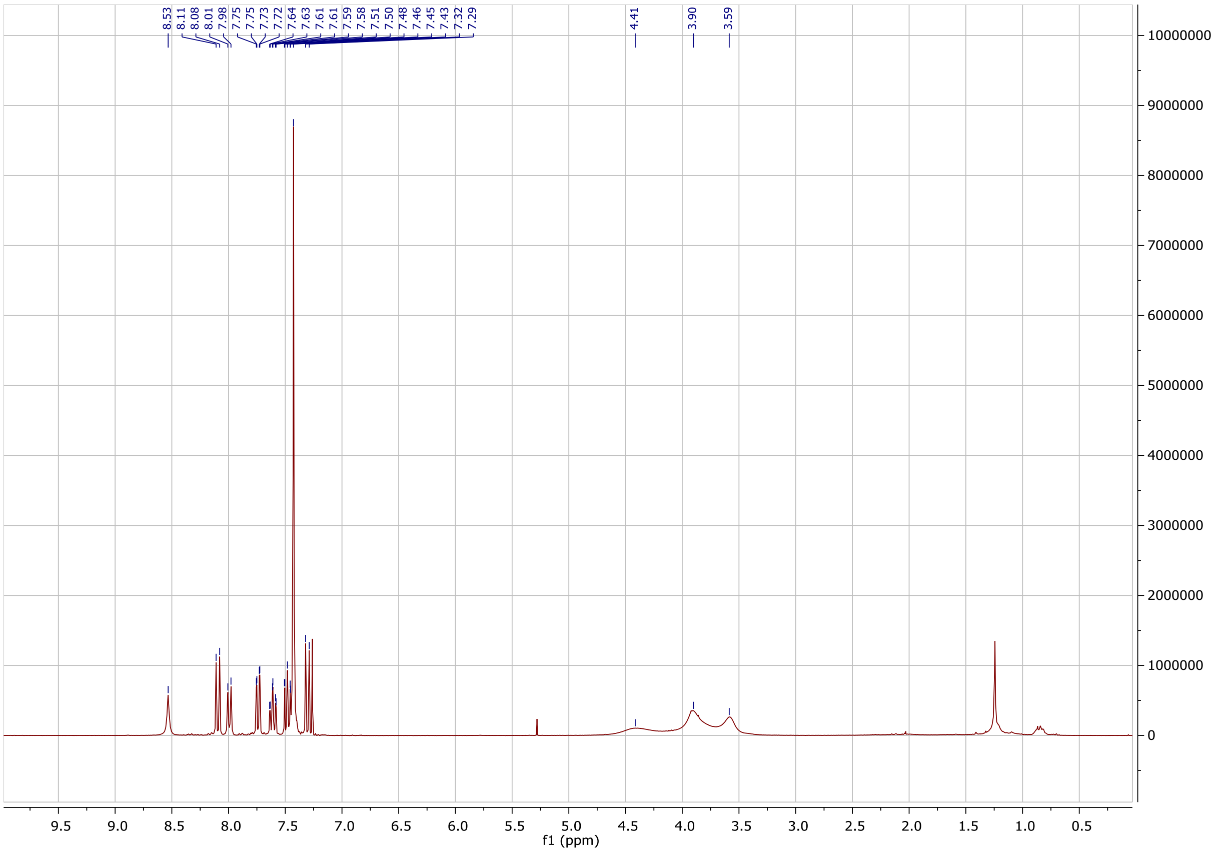
8.53
8.11
8.08
8.01
7.98
7.75
7.75
7.73
7.72
7.64
7.63
7.61
7.61
7.59
7.58
7.51
7.50
7.48
7.46
7.45
7.43
7.32
7.29
4.41
3.90
3.59
f1 (ppm)

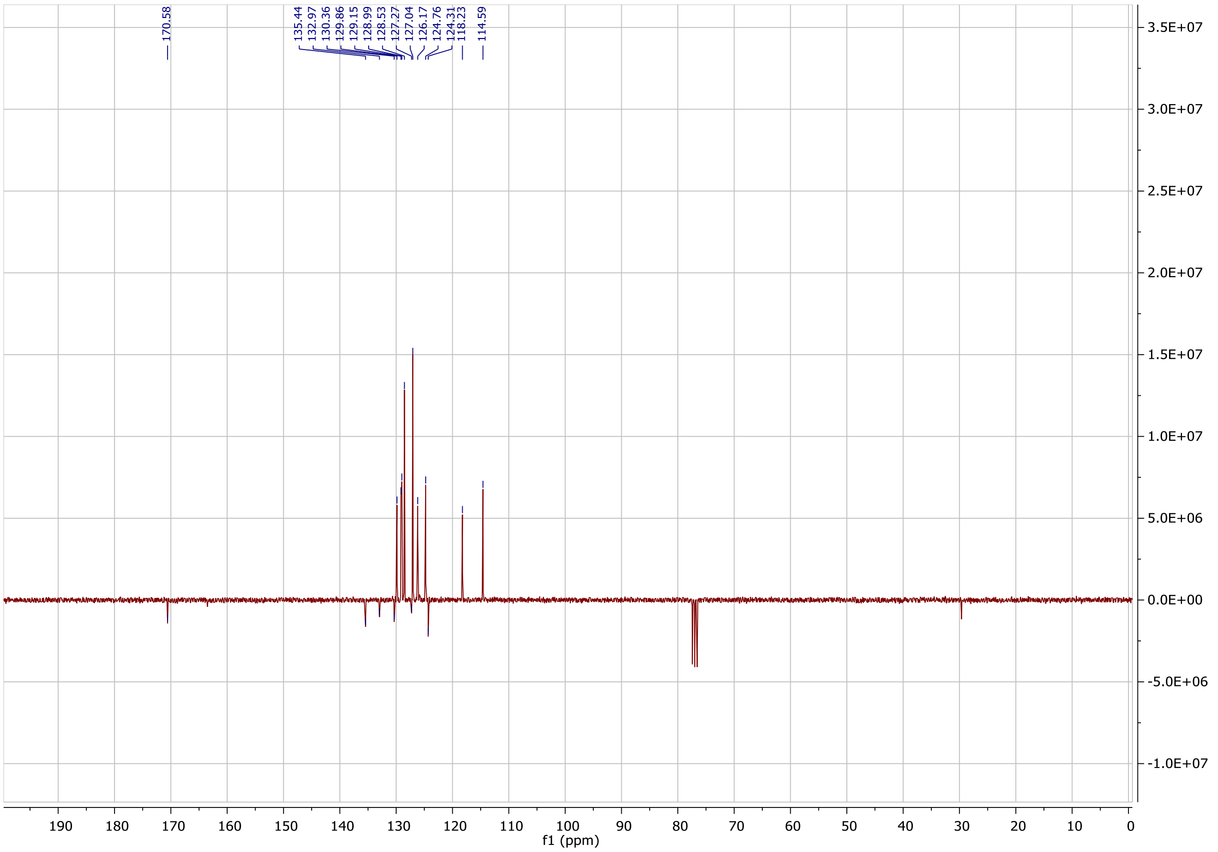
170.58
135.44
132.97
130.36
129.86
129.15
128.99
128.53
127.27
127.04
126.17
124.76
124.31
118.23
114.59
f1 (ppm)

Single Mass Analysis

Tolerance = 5.0 PPM / DBE: min = -1.5, max = 80.0
Element prediction: Off

Monoisotopic Mass, Odd and Even Electron Ions
177 formula(e) evaluated with 2 results within limits (all results (up to 1000) for each mass)
Elements Used:
C: 0-100 H: 0-100 N: 0-5 O: 0-5

DCI-CH4 GCT Premier CAB109 08-Jul-202210:03:47
20220708-CL9-58 27 (0.450) Cm (26:31-1:6x5.000) TOF MS CI+
2.04e+004

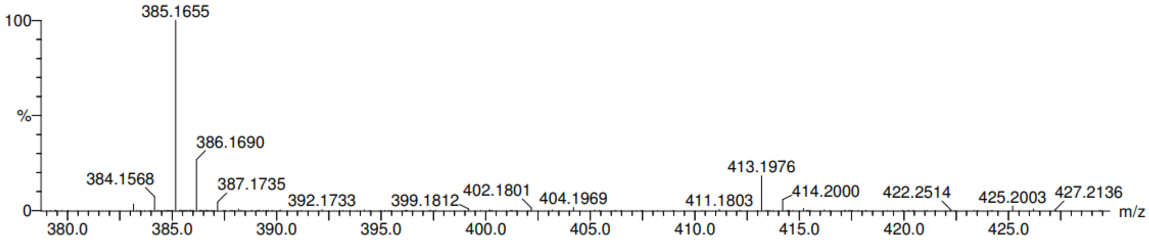

Minimum:				-1.5		
Maximum:		1.5	5.0	80.0		
Mass	Calc. Mass	mDa	PPM	DBE	i-FIT	Formula
385.1655	385.1665	-1.0	-2.6	15.5	3.3	C23 H21 N4 O2
	385.1638	1.7	4.4	11.0	29.5	C20 H23 N3 O5

Compound 4j

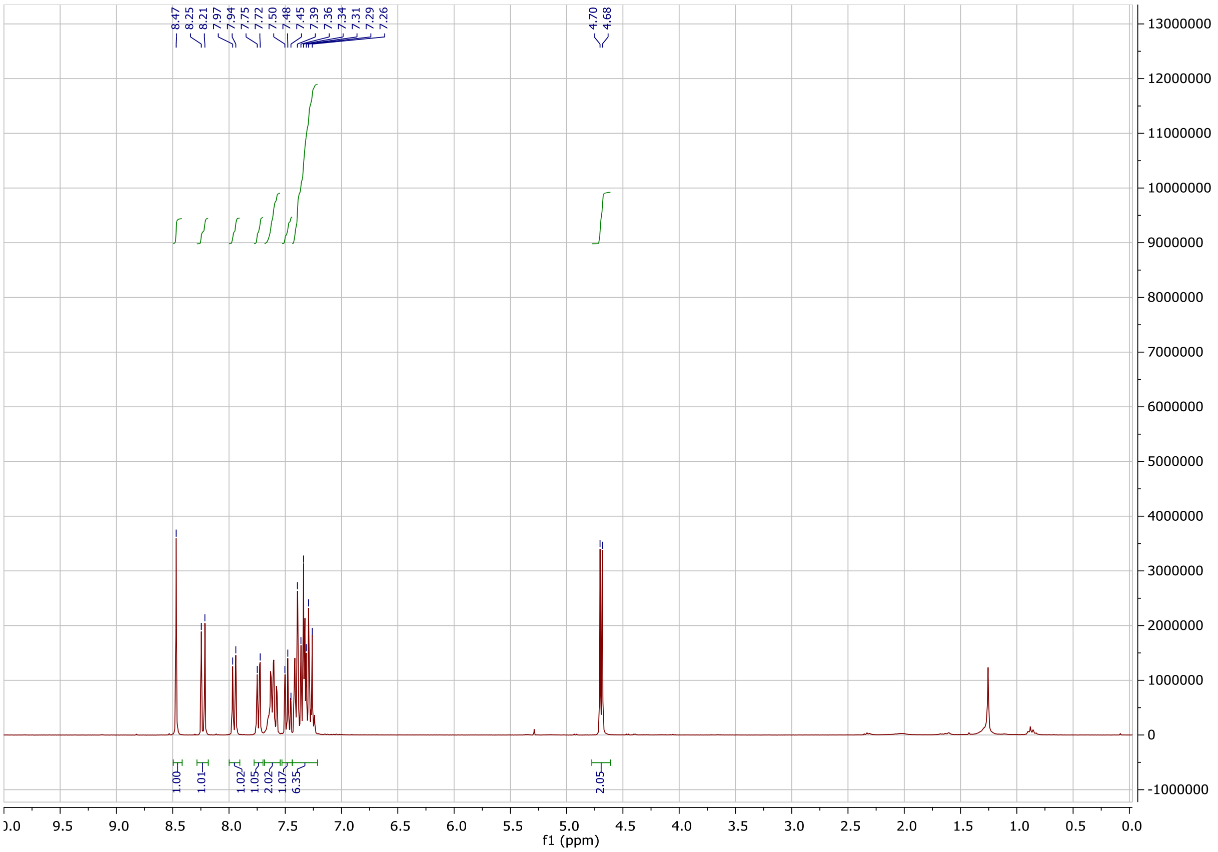
8.47
8.25
8.21
7.97
7.94
7.75
7.72
7.50
7.48
7.45
7.39
7.36
7.34
7.31
7.29
7.26
4.70
4.68
1.00
1.01
1.02
1.05
2.02
1.07
6.35
2.05
f1 (ppm)

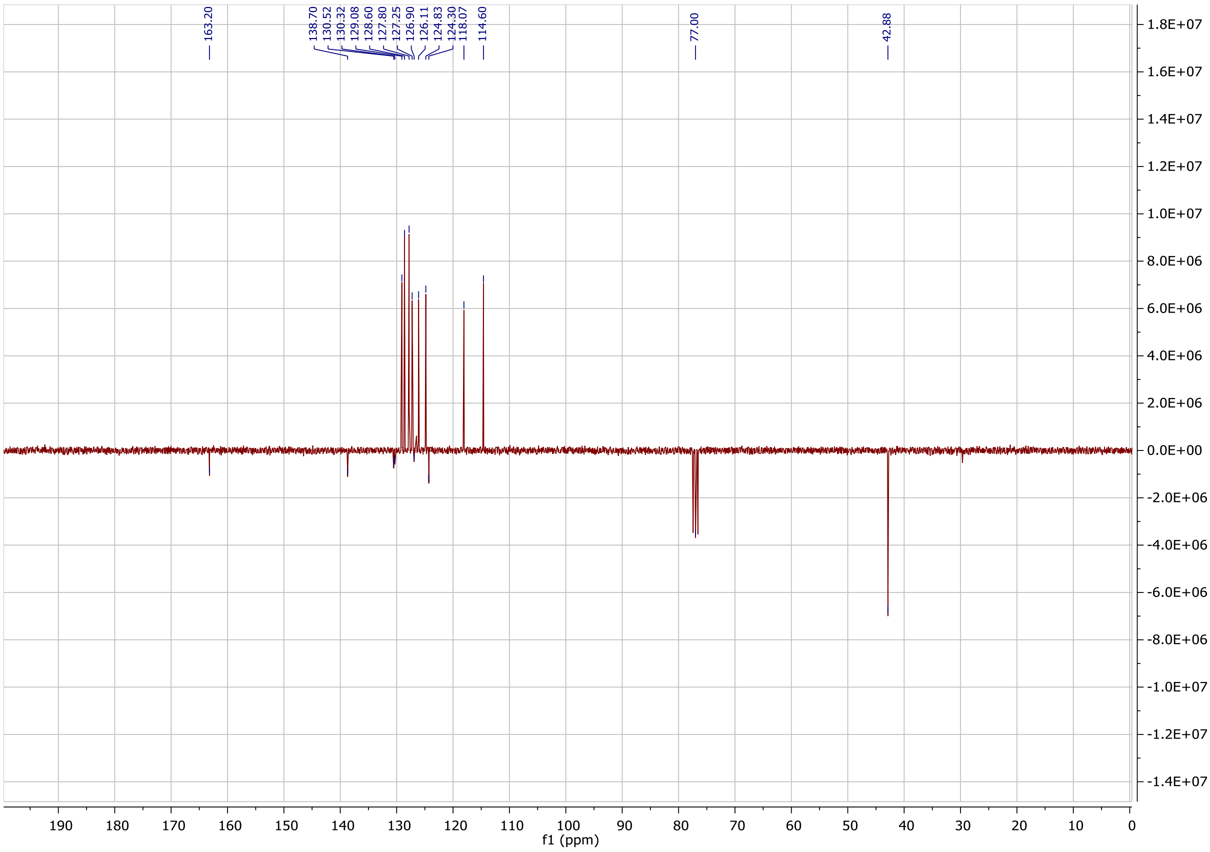
163.20
138.70
130.52
130.32
129.08
128.60
127.80
127.25
126.90
126.11
124.83
124.30
118.07
114.60
77.00
42.88
f1 (ppm)

Single Mass Analysis

Tolerance = 5.0 PPM / DBE: min = -1.5, max = 50.0
Element prediction: Off

Monoisotopic Mass, Odd and Even Electron Ions
138 formula(e) evaluated with 1 results within limits (all results (up to 1000) for each mass)
Elements Used:
C: 0-100 H: 0-100 N: 0-5 O: 0-5

DCI-CH4 GCT Premier CAB109 03-Jun-201910:13:13
20190603-CL9-19 21 (0.350) Cm (17:25-73:77x5.000) TOF MS CI+
5.37e+003

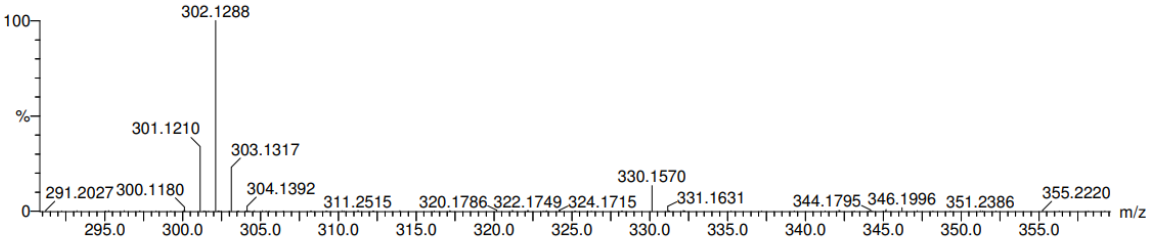

Minimum:				-1.5		
Maximum:		1.4	5.0	50.0		
Mass	Calc. Mass	mDa	PPM	DBE	i-FIT	Formula
302.1288	302.1293	-0.5	-1.7	13.5	1.7	C19 H16 N3 O

Compound 4k

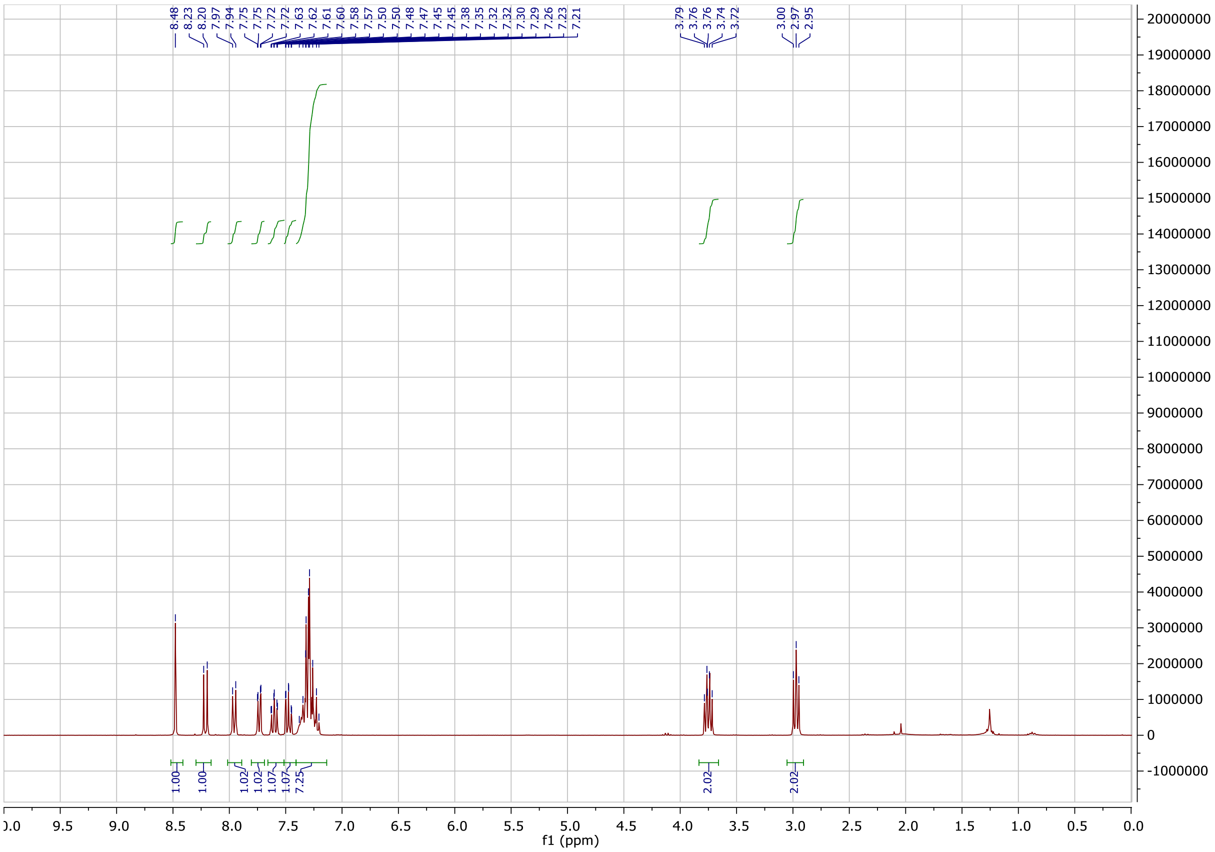

8.48
8.23
8.20
7.97
7.94
7.75
7.75
7.72
7.72
7.63
7.62
7.61
7.60
7.58
7.57
7.50
7.48
7.47
7.45
7.45
7.38
7.35
7.32
7.32
7.30
7.29
7.26
7.23
7.21
3.79
3.76
3.76
3.74
3.72
3.00
2.97
2.95
1.00
1.00
1.02
1.02
1.07
1.07
7.25
2.02
2.02
f1 (ppm)

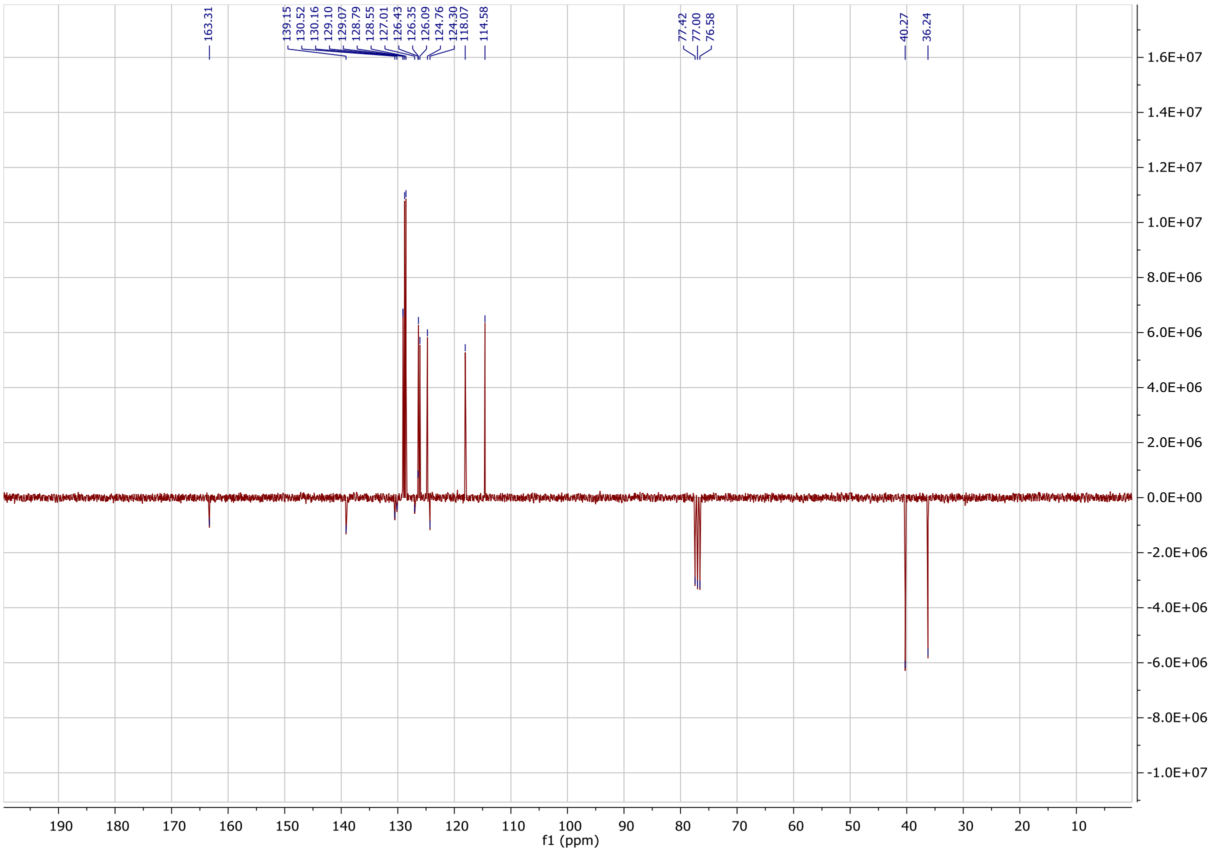

163.31
139.15
130.52
130.16
129.10
129.07
128.79
128.55
127.01
126.43
126.35
126.09
124.76
124.30
118.07
114.58
77.42
77.00
76.58
40.27
36.24
f1 (ppm)

Single Mass Analysis
Tolerance = 5.0 PPM / DBE: min = -1.5, max = 50.0
Element prediction: Off

Monoisotopic Mass, Odd and Even Electron Ions
398 formula(e) evaluated with 4 results within limits (all results (up to 1000) for each mass)
Elements Used:
C: 0-100 H: 0-100 N: 0-10 O: 0-10

DCI-CH4 GCT Premier CAB109 27-Jun-201910:34:00
20190627-CL9-25 27 (0.450) Cm (27:34-113:117x5.000) TOF MS CI+
2.36e+004

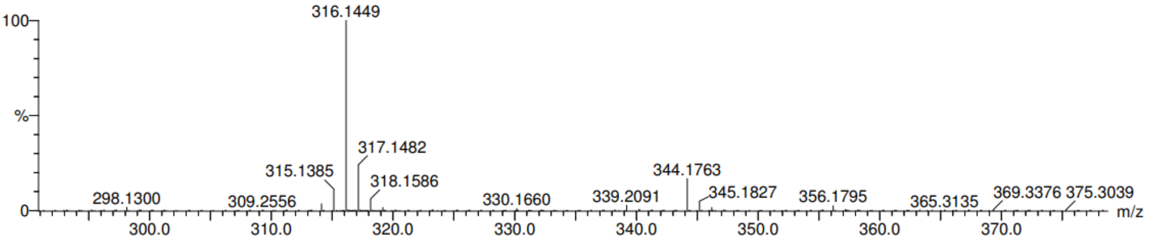

Minimum:				-1.5		
Maximum:		1.4	5.0	50.0		
Mass	Calc. Mass	mDa	PPM	DBE	i-FIT	Formula
316.1449	316.1463	-1.4	-4.4	13.0	136.8	C22 H20 O2
	316.1450	-0.1	-0.3	13.5	181.7	C20 H18 N3 O
	316.1436	1.3	4.1	14.0	249.2	C18 H16 N6
	316.1455	-0.6	-1.9	1.0	1189.5	C6 H20 N8 O7

Compound 4l

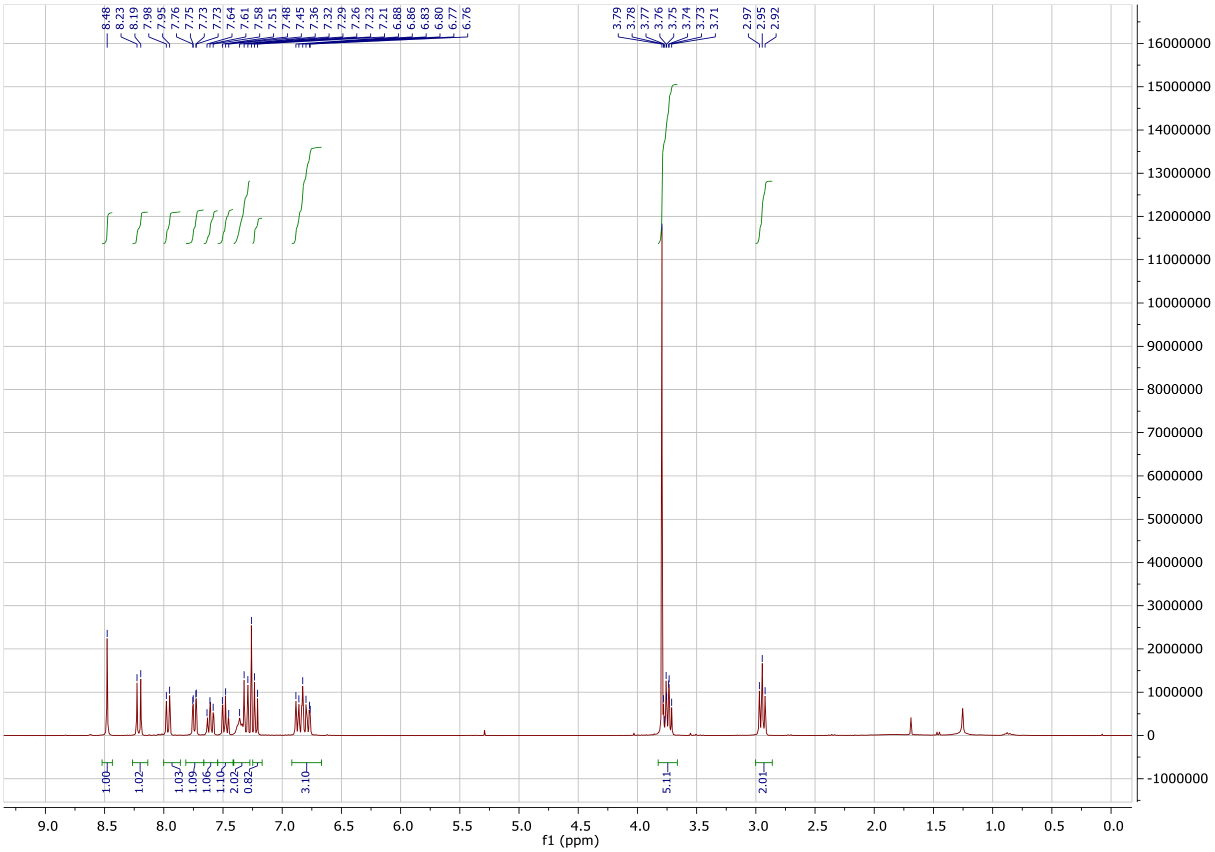

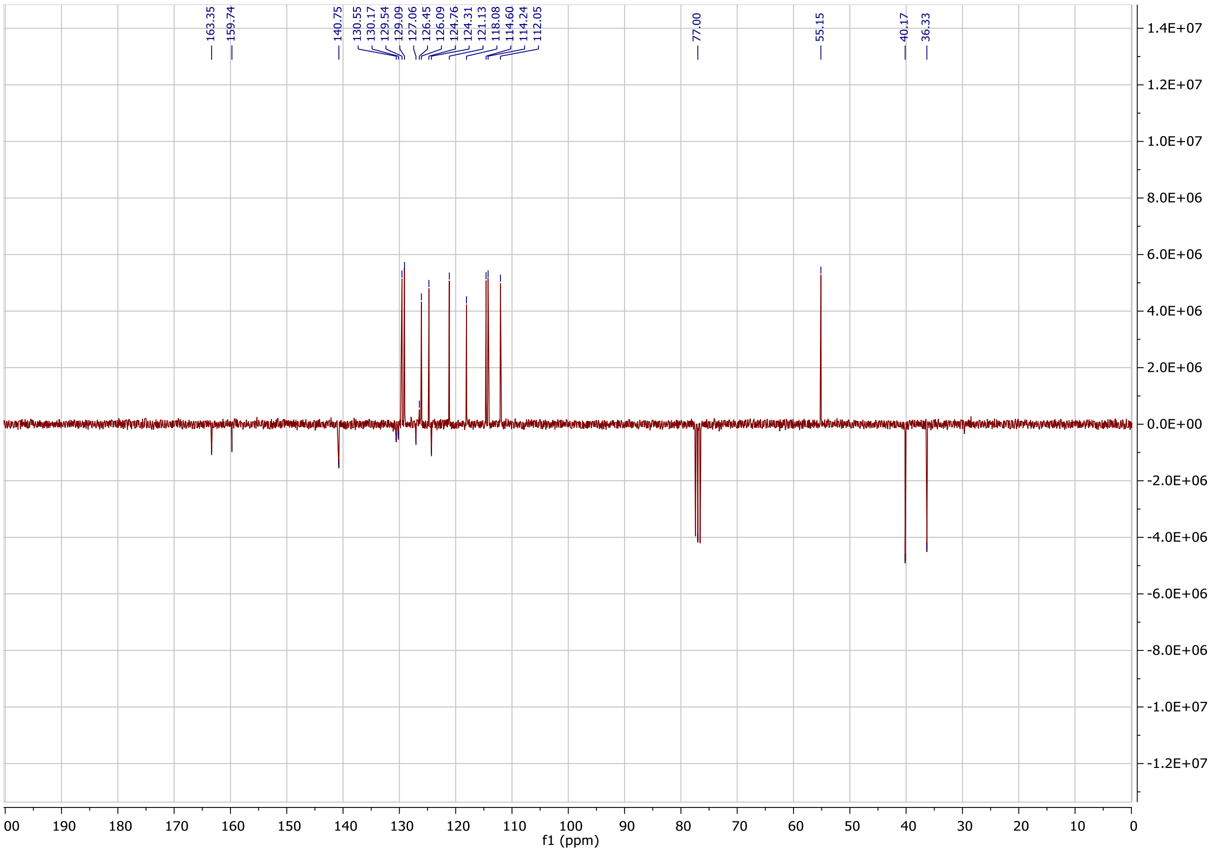

Single Mass Analysis

Tolerance = 5.0 PPM / DBE: min = -1.5, max = 80.0
Element prediction: Off

Monoisotopic Mass, Odd and Even Electron Ions
227 formula(e) evaluated with 2 results within limits (all results (up to 1000) for each mass)
Elements Used:
C: 0-100 H: 0-100 N: 0-8 O: 0-5

DCI-CH4 GCT Premier CAB109 07-Jul-202211:25:04
20220707-CL9-36 38 (0.634) Cm (38:47-164:171x5.000) TOF MS CI+
1.67e+004

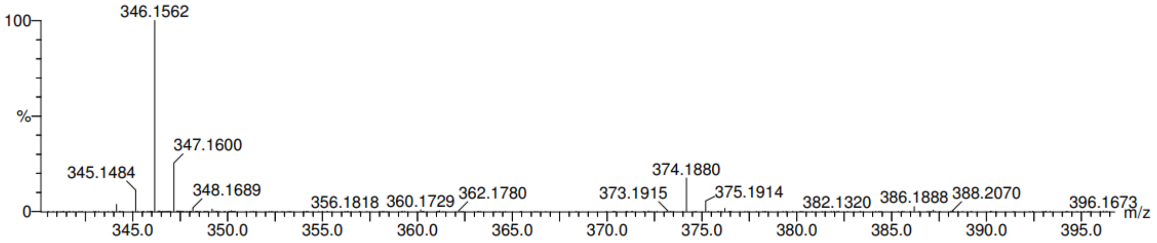

Minimum:				-1.5		
Maximum:		1.5	5.0	80.0		
Mass	Calc. Mass	mDa	PPM	DBE	i-FIT	Formula
346.1562	346.1556	0.6	1.7	13.5	124.1	C21 H20 N3 O2
	346.1569	-0.7	-2.0	13.0	201.0	C23 H22 O3

Compound 4m

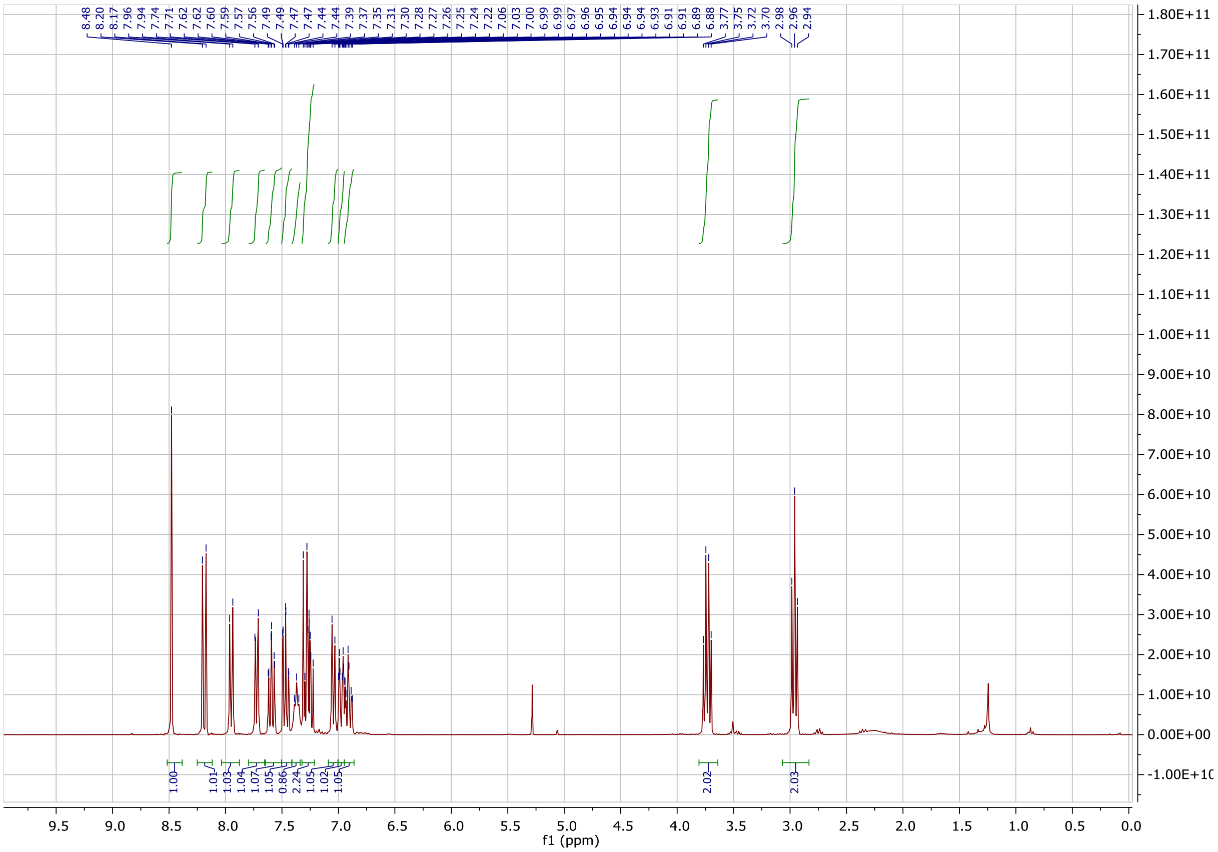
8.48
8.20
8.17
7.96
7.94
7.74
7.71
7.62
7.62
7.60
7.59
7.57
7.56
7.49
7.49
7.47
7.47
7.44
7.44
7.39
7.37
7.35
7.31
7.30
7.28
7.27
7.26
7.25
7.24
7.22
7.06
7.03
7.00
6.99
6.99
6.97
6.96
6.95
6.94
6.94
6.93
6.91
6.89
6.88
3.77
3.75
3.72
3.70
2.98
2.96
2.94
1.00
1.01
1.03
1.04
1.07
1.05
0.86
2.24
1.05
1.02
1.05
2.02
2.03
f1 (ppm)

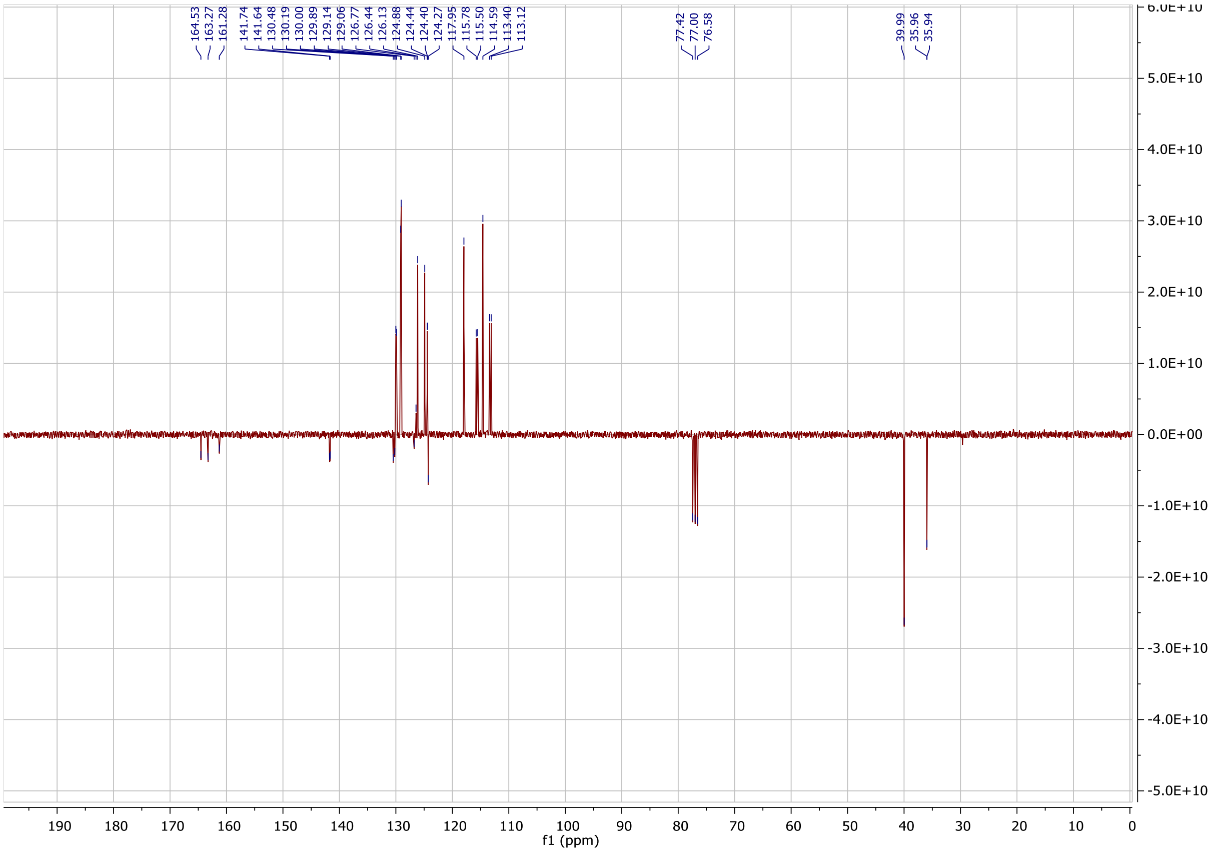
164.53
163.27
161.28
141.74
141.64
130.48
130.19
130.00
129.89
129.14
129.06
126.77
126.44
126.13
124.88
124.44
124.40
124.27
117.95
115.78
115.50
114.59
113.40
113.12
77.42
77.00
76.58
39.99
35.96
35.94
f1 (ppm)

Single Mass Analysis

Tolerance = 5.0 PPM / DBE: min = -1.5, max = 80.0
Element prediction: Off

Monoisotopic Mass, Odd and Even Electron Ions
210 formula(e) evaluated with 2 results within limits (all results (up to 1000) for each mass)
Elements Used:
C: 0-100 H: 0-100 N: 0-8 O: 0-5 F: 1-1

DCI-CH4 | GCT Premier CAB109 | 08-Jul-202209:55:19
20220708-CL9-60 22 (0.367) Cm (21:24-2:4x5.000) | TOF MS CI+ 7.23e+004

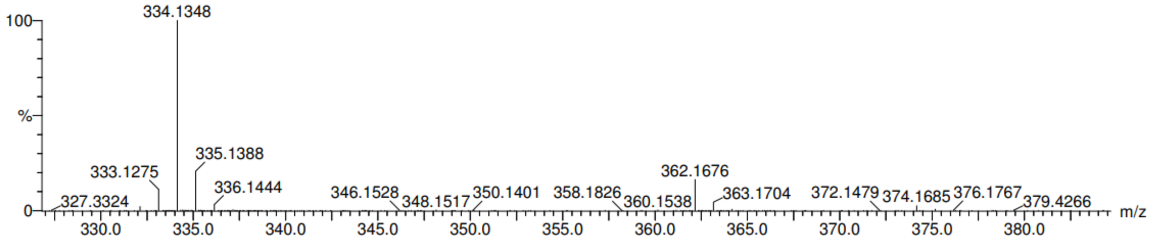

Minimum:				-1.5		
Maximum:		1.5	5.0	80.0		
Mass	Calc. Mass	mDa	PPM	DBE	i-FIT	Formula
334.1348	334.1342	0.6	1.8	14.0	101.2	C18 H15 N6 F
	334.1356	-0.8	-2.4	13.5	140.1	C20 H17 N3 O F

Compound 4n

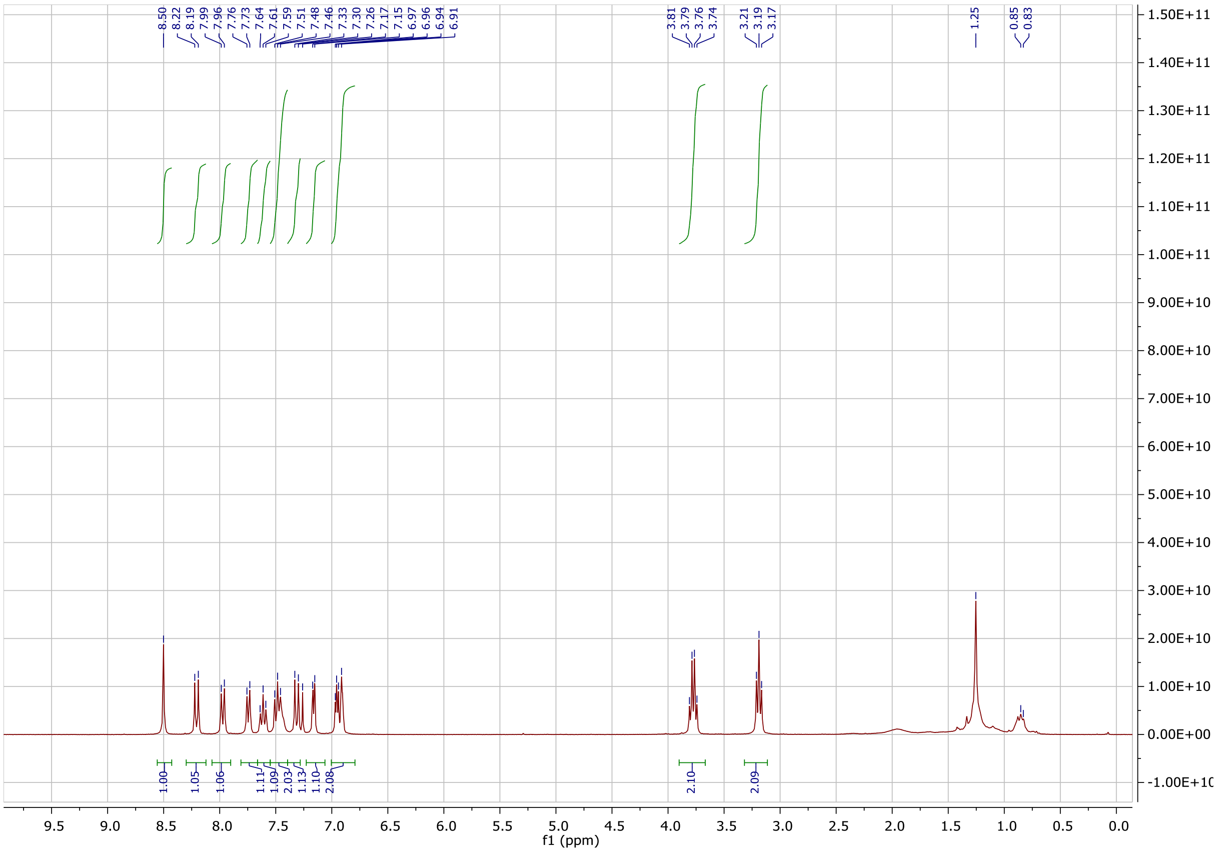
8.50
8.22
8.19
7.99
7.96
7.76
7.73
7.64
7.61
7.59
7.51
7.48
7.46
7.33
7.30
7.26
7.17
7.15
6.97
6.96
6.94
6.91
3.81
3.79
3.76
3.74
3.21
3.19
3.17
1.25
0.85
0.83
1.00
1.05
1.06
1.11
1.09
2.03
1.13
1.10
2.08
2.10
2.09
f1 (ppm)

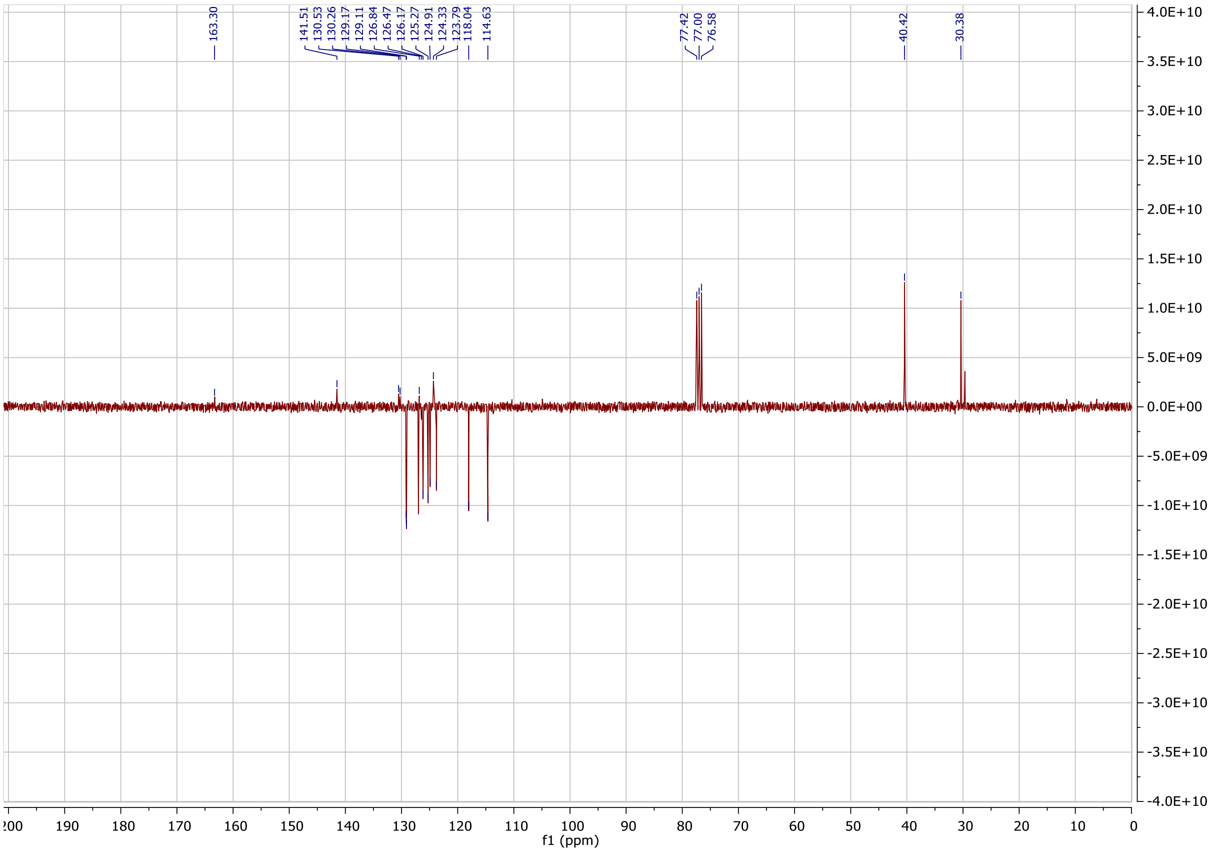
163.30
141.51
130.53
130.26
129.17
129.11
126.84
126.47
126.17
125.27
124.91
124.33
123.79
118.04
114.63
77.42
77.00
76.58
40.42
30.38
f1 (ppm)

Single Mass Analysis

Tolerance = 5.0 PPM / DBE: min = -1.5, max = 80.0
Element prediction: Off

Monoisotopic Mass, Odd and Even Electron Ions
191 formula(e) evaluated with 3 results within limits (all results (up to 1000) for each mass)
Elements Used:
C: 0-100 H: 0-100 N: 0-8 O: 0-5 S: 1-1

DCI-CH4 | GCT Premier CAB109 | 08-Jul-202209:47:46
20220708-CL9-61 29 (0.483) Cm (29:35-1:6x5.000) | TOF MS CI+ 1.43e+004

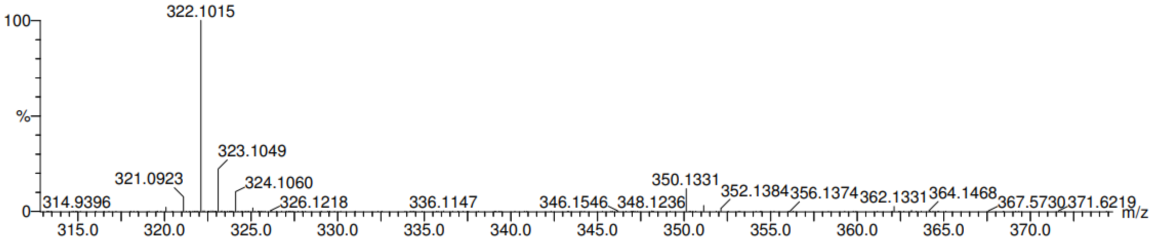

Minimum:				-1.5		
Maximum:		1.5	5.0	80.0		
Mass	Calc. Mass	mDa	PPM	DBE	i-FIT	Formula
322.1015	322.1014	0.1	0.3	12.5	68.2	C18 H16 N3 O S
	322.1028	-1.3	-4.0	12.0	57.4	C20 H18 O2 S
	322.1001	1.4	4.3	13.0	90.7	C16 H14 N6 S

Compound 4o

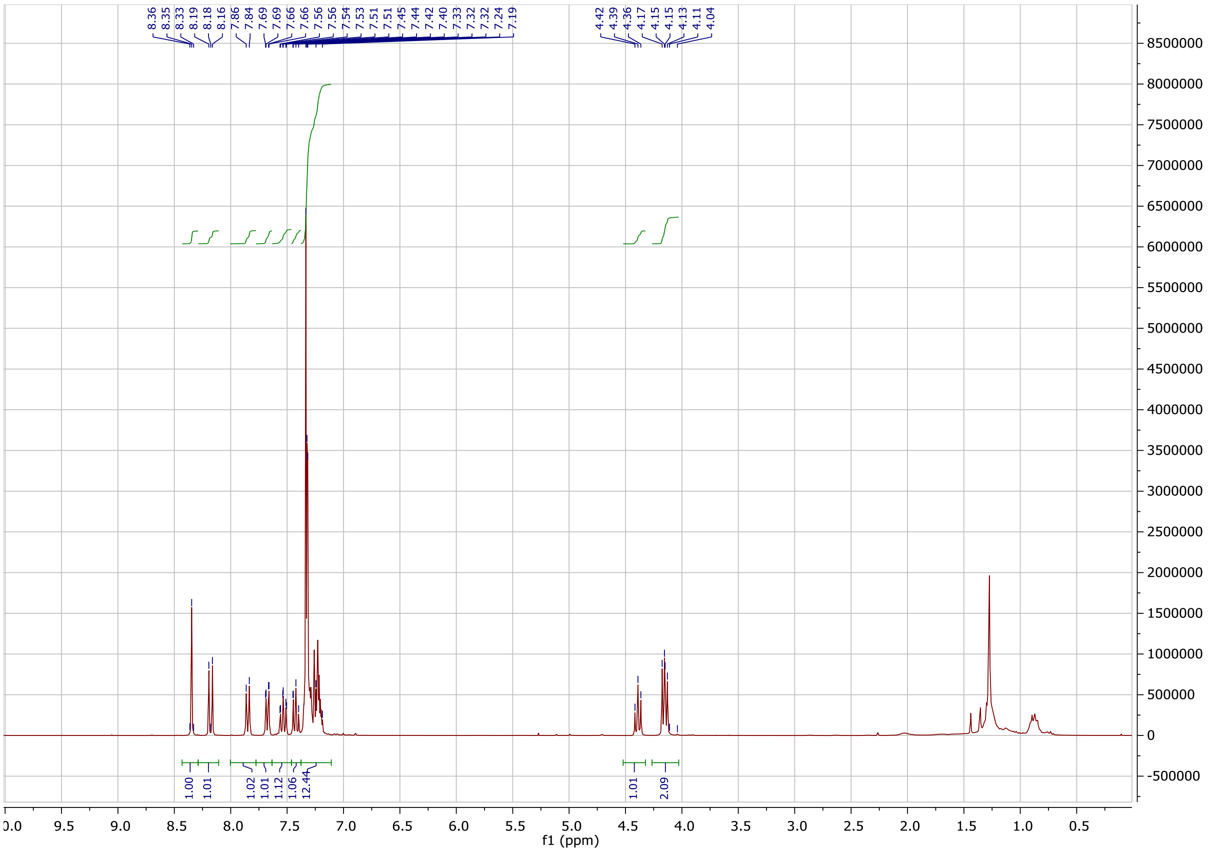

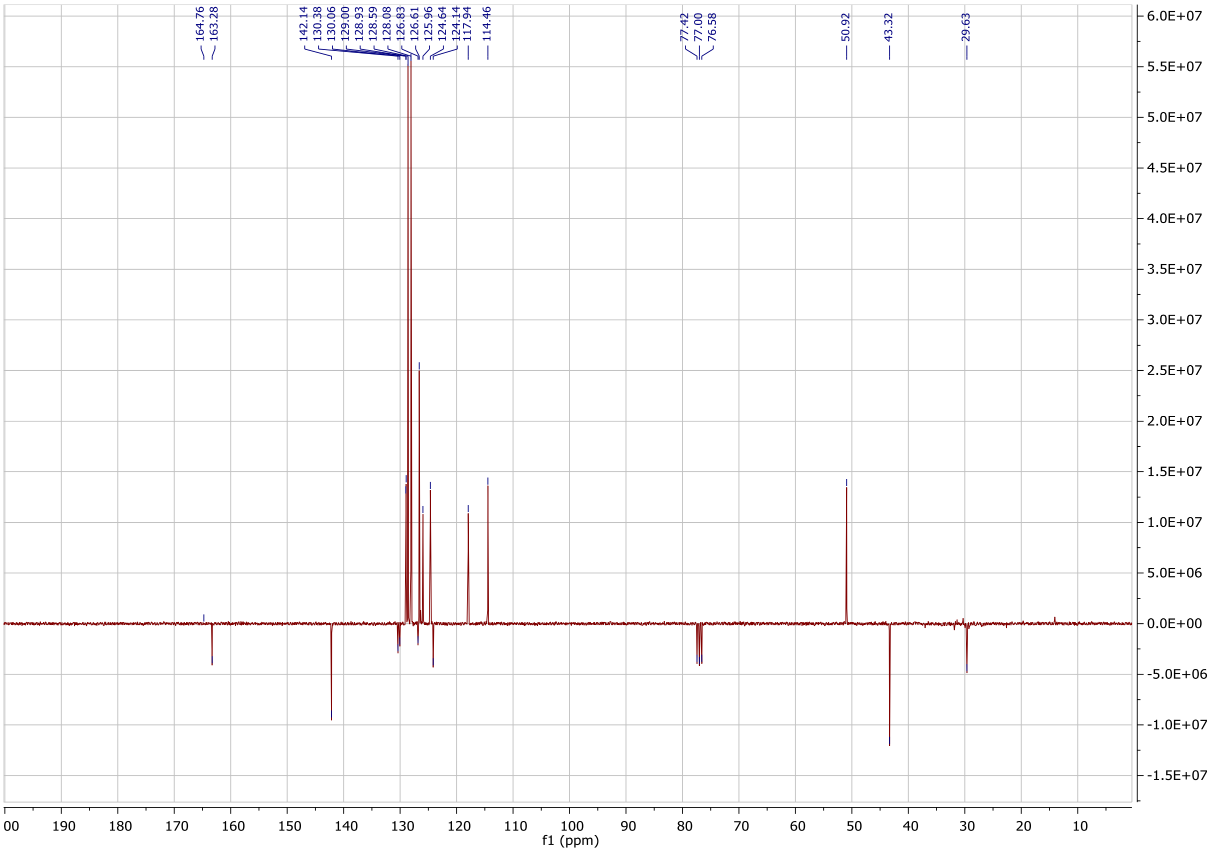

Single Mass Analysis
Tolerance = 5.0 PPM / DBE: min = -1.5, max = 80.0
Element prediction: Off

Monoisotopic Mass, Odd and Even Electron Ions
259 formula(e) evaluated with 2 results within limits (all results (up to 1000) for each mass)
Elements Used:
C: 0-100 H: 0-100 N: 0-8 O: 0-5

DCI-CH4 GCT Premier CAB109 07-Jul-202211:38:04
20220707-CL9-59 103 (1.717) Cm (102:112-3:11x5.000) TOF MS CI+ 3.42e+004

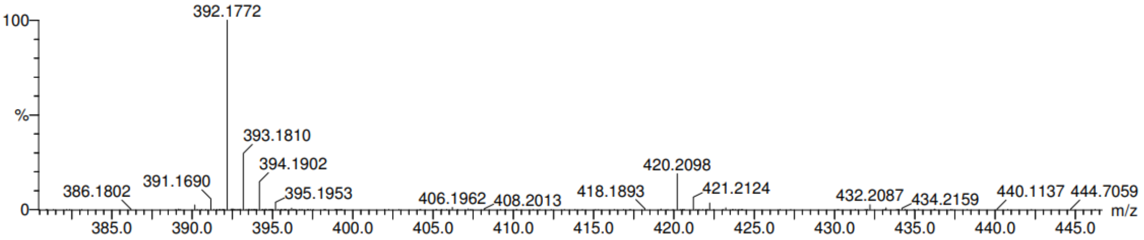

Minimum:				-1.5		
Maximum:		1.5	5.0	80.0		
Mass	Calc. Mass	mDa	PPM	DBE	i-FIT	Formula
392.1772	392.1776	-0.4	-1.0	17.0	1049.0	C28 H24 O2
	392.1763	0.9	2.3	17.5	1150.7	C26 H22 N3 O

Compound 4p

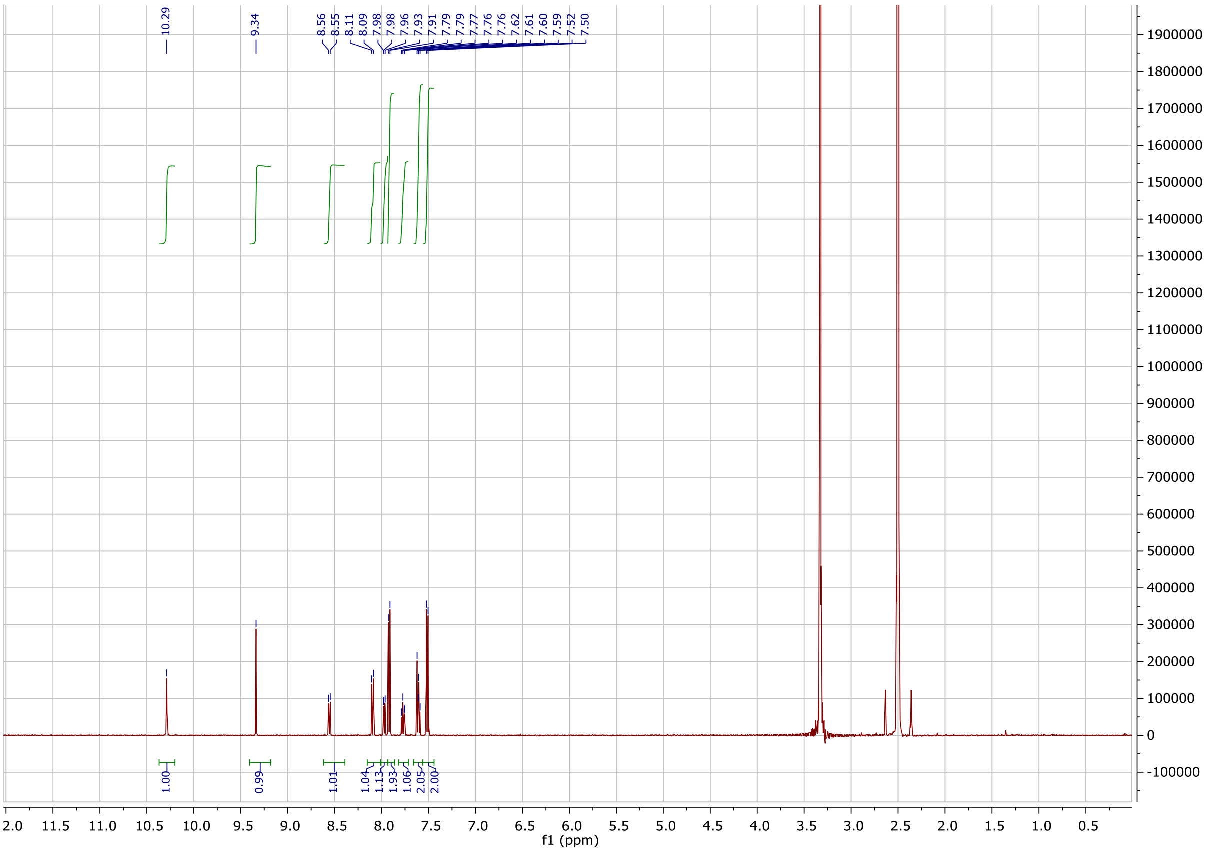
10.29
9.34
8.56
8.55
8.11
8.09
7.98
7.98
7.96
7.93
7.91
7.79
7.79
7.77
7.76
7.62
7.61
7.60
7.59
7.52
7.50
1.00
0.99
1.01
1.04
1.13
1.93
1.06
2.05
2.00
f1 (ppm)

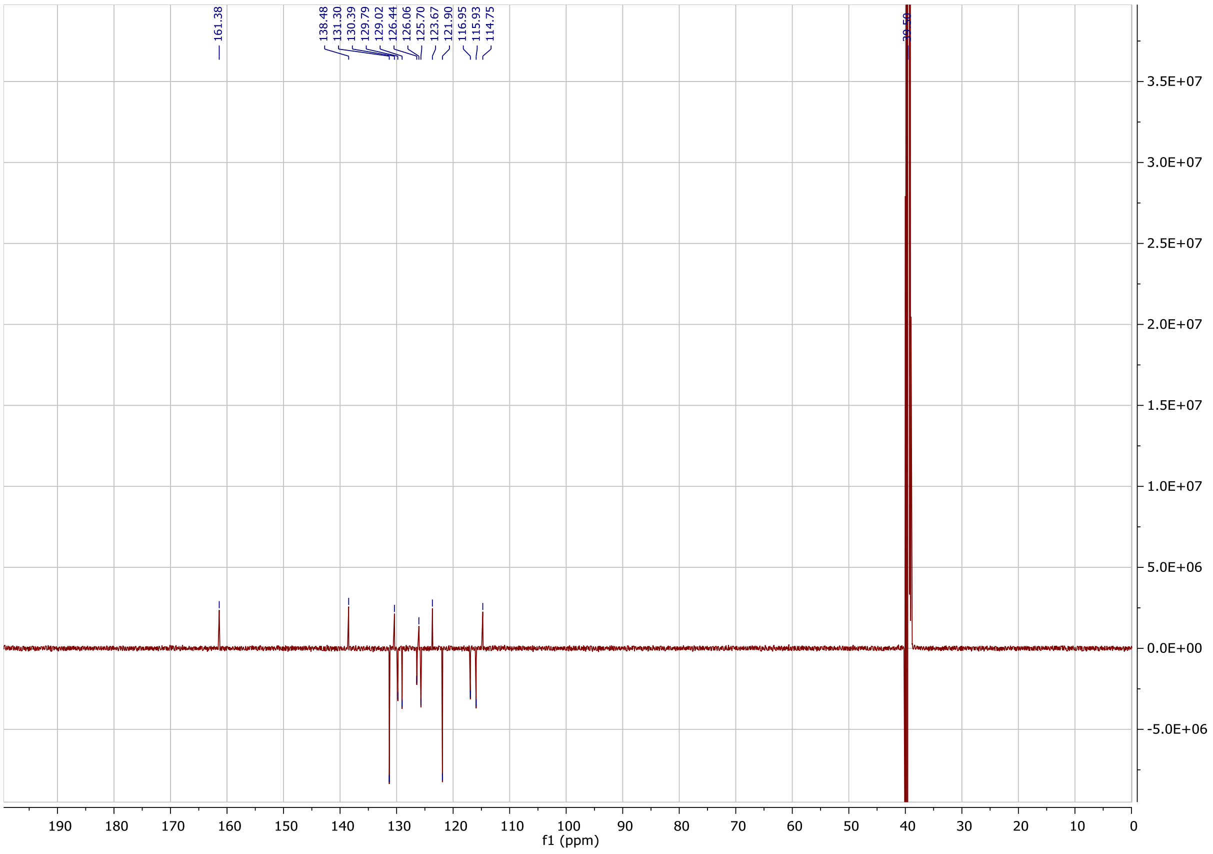
161.38
138.48
131.30
130.39
129.79
129.02
126.44
126.06
125.70
123.67
121.90
116.95
115.93
114.75
39.50
f1 (ppm)

Single Mass Analysis

Tolerance = 3.0 PPM / DBE: min = -5.0, max = 100.0
Element prediction: Off
Number of isotope peaks used for i-FIT = 3

Monoisotopic Mass, Even Electron Ions
948 formula(e) evaluated with 1 results within limits (up to 50 closest results for each mass)
Elements Used:
C: 0-80 H: 0-100 N: 0-10 O: 0-10 Br: 0-1

Cone voltage = 30 V Xevo G2 YCA210 03-Jun-2019 14:48:53
CL9-17 76 (0.476) AM2 (Ar,20000.0,0.00,0.00); Cm (74:82-115:120x2.000) 1: TOF MS ES+
2.51e+005

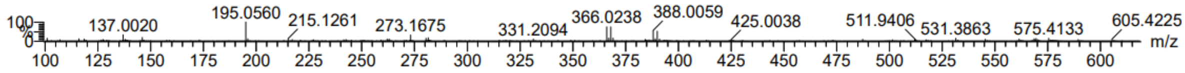


```
Minimum:                             -5.0
Maximum:                  1.0   3.0  100.0
```

Mass	Calc. Mass	mDa	PPM	DBE	i-FIT	Norm	Conf(%)	Formula
366.0238	366.0242	-0.4	-1.1	13.5	537.8	n/a	n/a	C18 H13 N3 O Br

Compound 4q

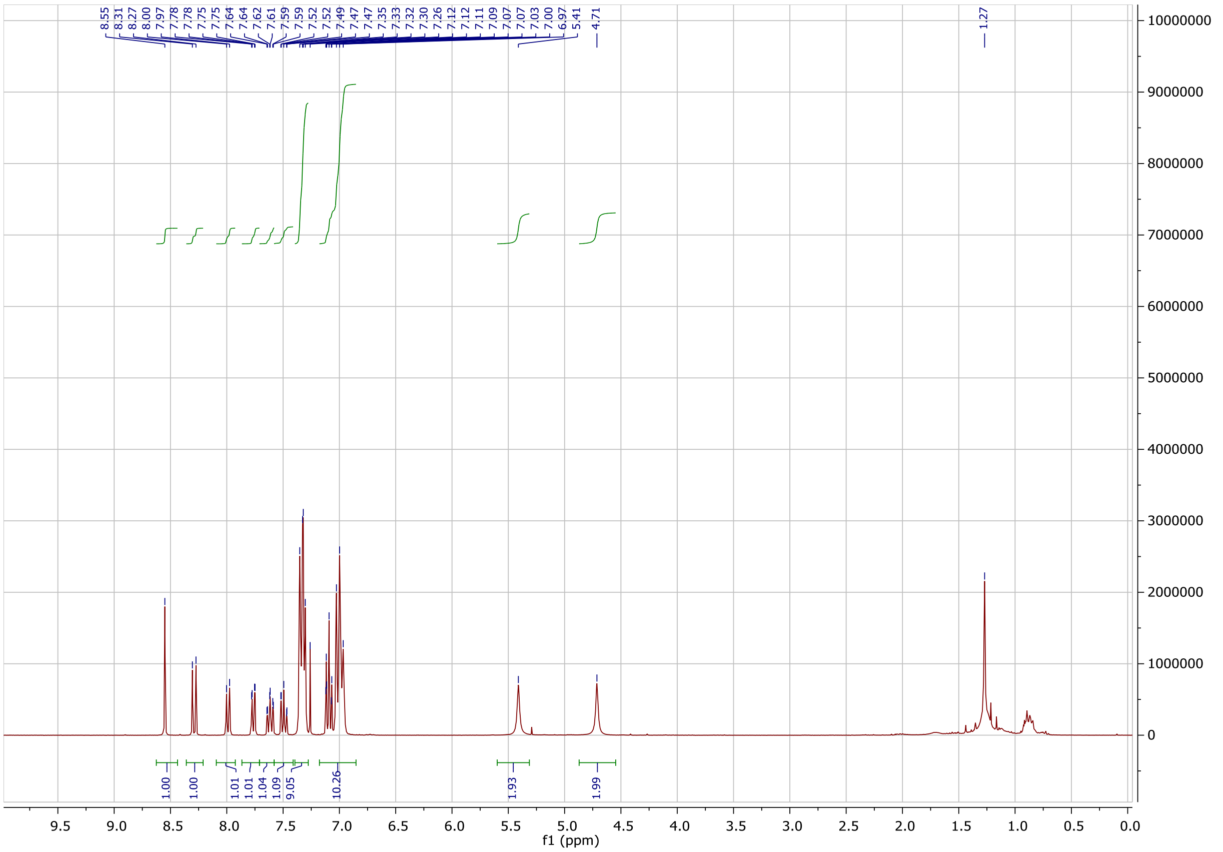
8.55
8.31
8.27
8.00
7.97
7.78
7.78
7.75
7.75
7.64
7.64
7.62
7.61
7.59
7.59
7.52
7.52
7.49
7.47
7.47
7.35
7.33
7.32
7.30
7.26
7.12
7.12
7.11
7.09
7.07
7.07
7.03
7.00
6.97
5.41
4.71
1.27
1.00
1.00
1.01
1.01
1.04
1.09
9.05
10.26
1.93
1.99
f1 (ppm)

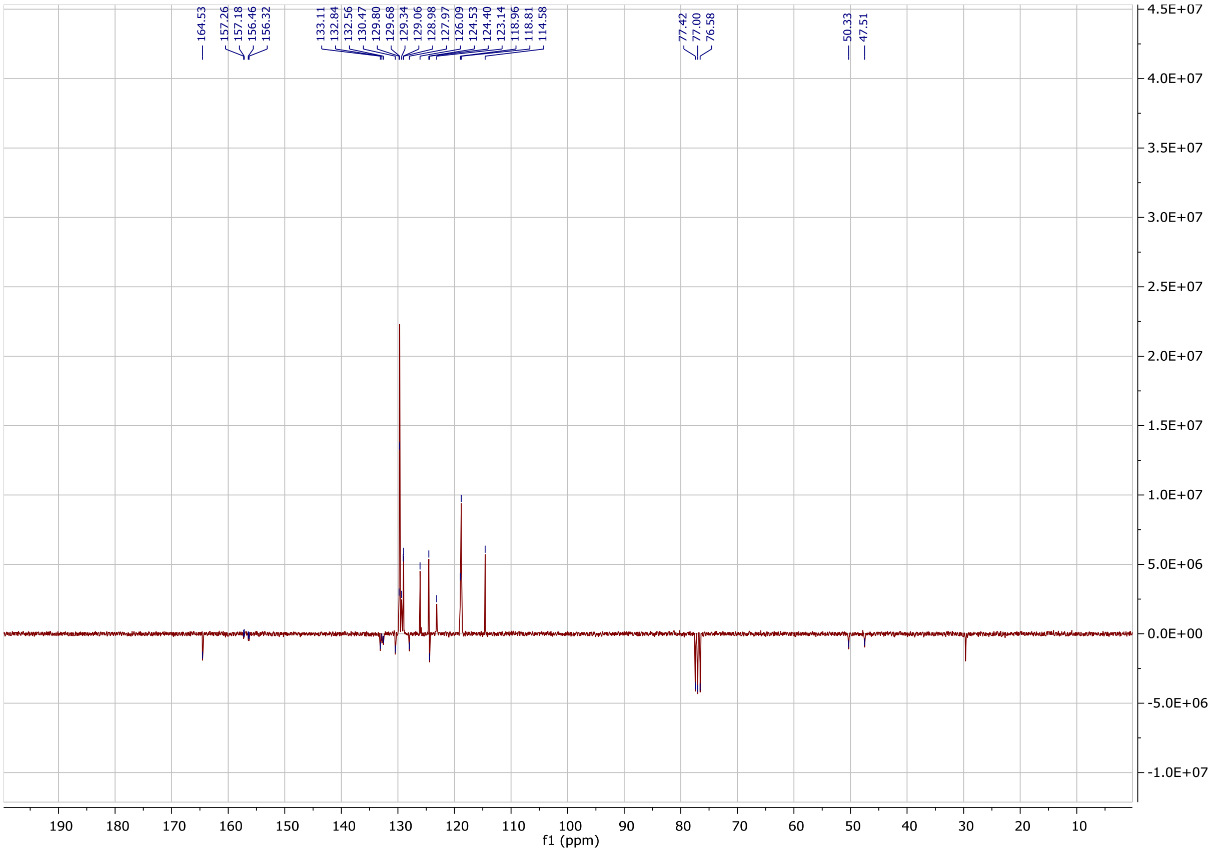
164.53
157.26
157.18
156.46
156.32
133.11
132.84
132.56
130.47
129.80
129.68
129.34
129.06
128.98
127.97
126.09
124.53
124.40
123.14
118.96
118.81
114.58
77.42
77.00
76.58
50.33
47.51
f1 (ppm)

Single Mass Analysis

Tolerance = 5.0 PPM / DBE: min = -1.5, max = 80.0
Element prediction: Off

Monoisotopic Mass, Odd and Even Electron Ions
258 formula(e) evaluated with 3 results within limits (all results (up to 1000) for each mass)
Elements Used:
C: 0-100 H: 0-100 N: 0-5 O: 0-5

DCI-CH4 GCT Premier CAB109 07-Jul-202211:07:50
20220707-CL9-64 31 (0.517) Cm (30:32-55:57x5.000) TOF MS CI+
8.79e+003

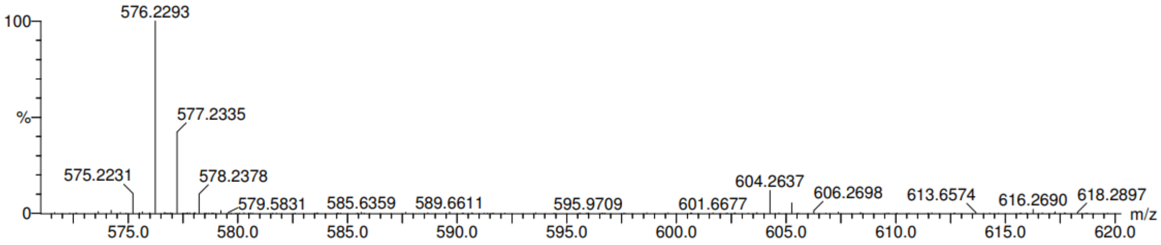

Minimum:				-1.5		
Maximum:		1.5	5.0	80.0		
Mass	Calc. Mass	mDa	PPM	DBE	i-FIT	Formula
576.2293	576.2287	0.6	1.0	25.5	2.7	C38 H30 N3 O3
	576.2301	-0.8	-1.4	25.0	8.6	C40 H32 O4
	576.2314	-2.1	-3.6	30.0	22.9	C41 H28 N4

Compound 4r

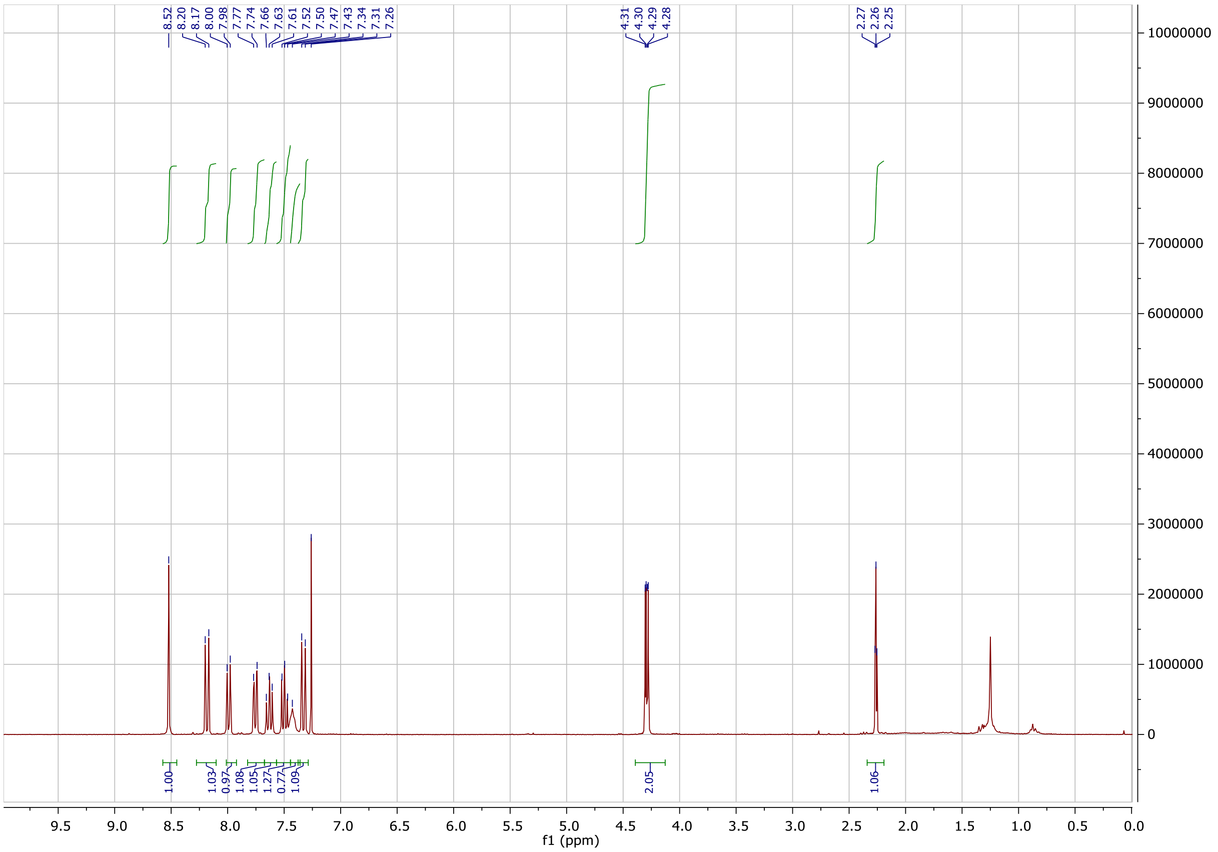

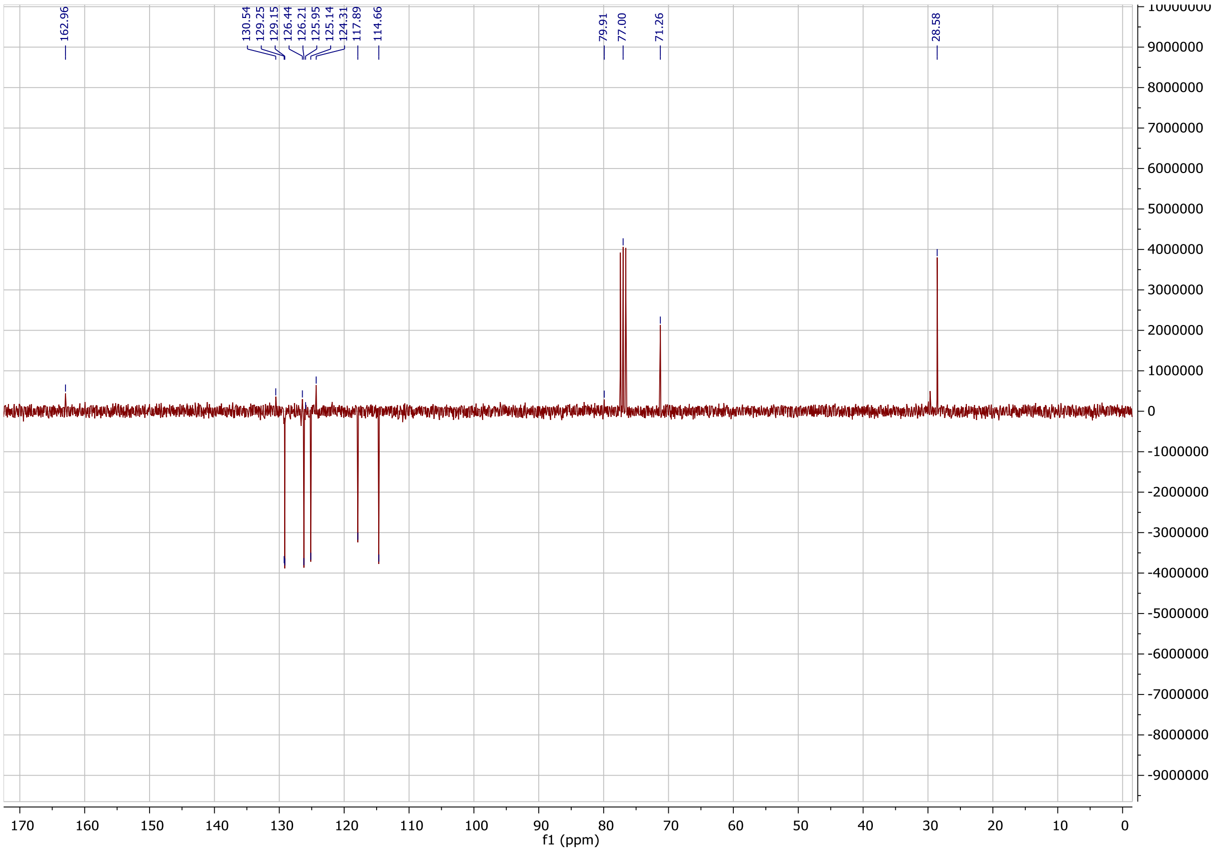

Single Mass Analysis

Tolerance = 5.0 PPM / DBE: min = -1.5, max = 80.0
Element prediction: Off

Monoisotopic Mass, Odd and Even Electron Ions
116 formula(e) evaluated with 1 results within limits (all results (up to 1000) for each mass)
Elements Used:
C: 0-100 H: 0-100 N: 0-5 O: 0-5

DCI-CH4 GCT Premier CAB109 07-Jul-202211:00:54
20220707-CL9-49 34 (0.567) Cm (27:34-161:172x5.000) TOF MS CI+
3.28e+004

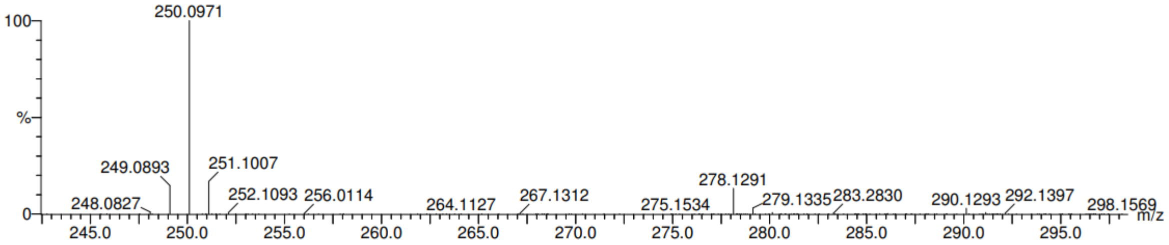

Minimum:				-1.5		
Maximum:		1.5	5.0	80.0		
Mass	Calc. Mass	mDa	PPM	DBE	i-FIT	Formula
250.0971	250.0980	-0.9	-3.6	11.5	373.7	C15 H12 N3 O

Compound 4s

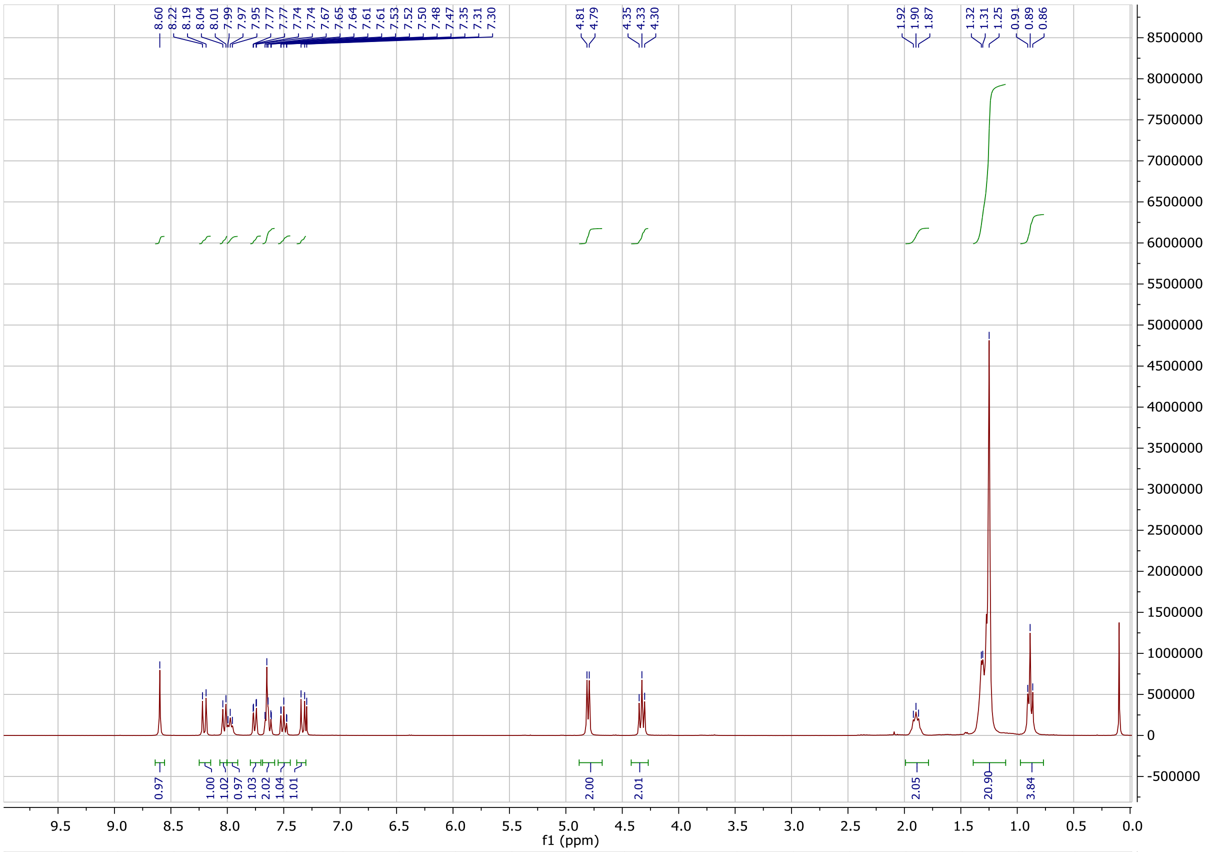
8.60
8.22
8.19
8.04
8.01
7.99
7.97
7.95
7.77
7.77
7.74
7.74
7.67
7.65
7.64
7.61
7.61
7.53
7.52
7.50
7.48
7.47
7.35
7.31
7.30
4.81
4.79
4.35
4.33
4.30
1.92
1.90
1.87
1.32
1.31
1.25
0.91
0.89
0.86
0.97
1.00
1.02
0.97
1.03
2.02
1.04
1.01
2.00
2.01
2.05
20.90
3.84
f1 (ppm)

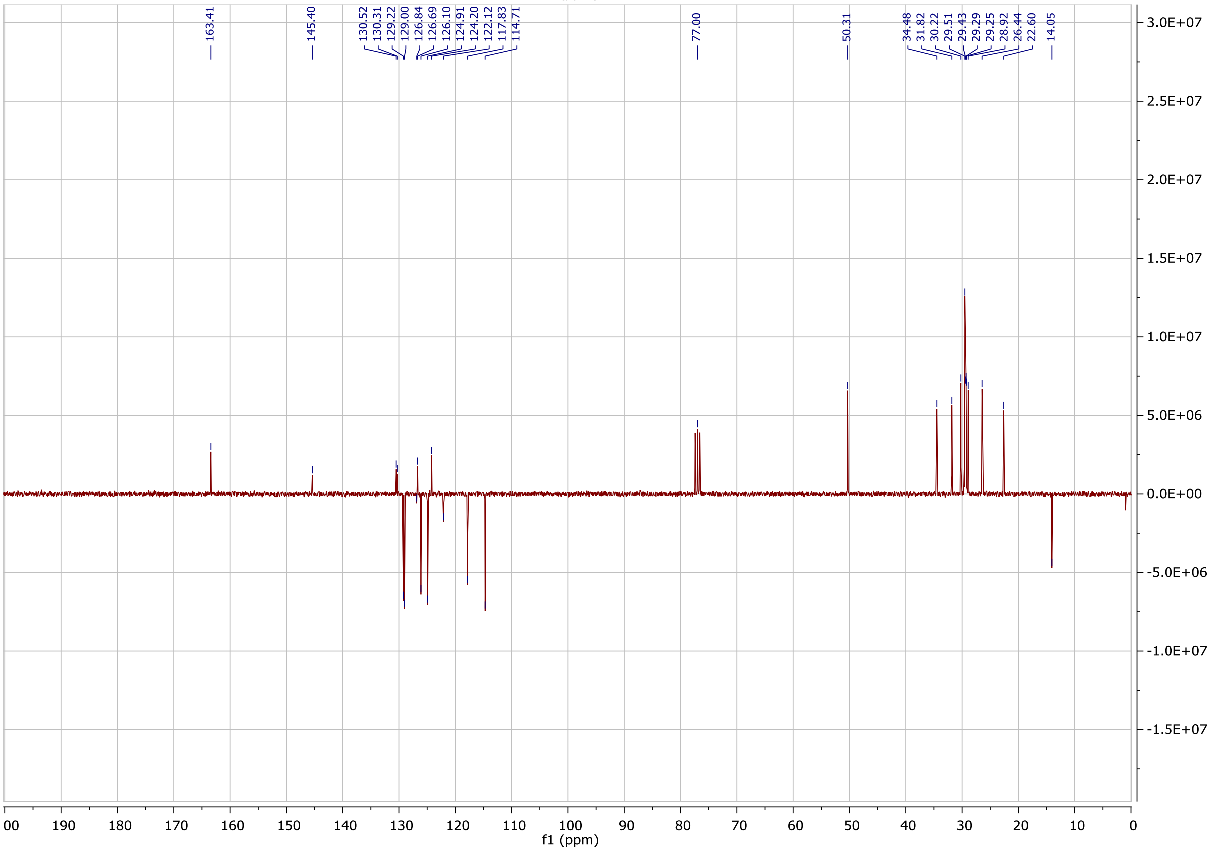
163.41
145.40
130.52
130.31
129.22
129.00
126.84
126.69
126.10
124.91
124.20
122.12
117.83
114.71
77.00
50.31
34.48
31.82
30.22
29.51
29.43
29.29
29.25
28.92
26.44
22.60
14.05
f1 (ppm)

Single Mass Analysis

Tolerance = 5.0 PPM / DBE: min = -1.5, max = 80.0
Element prediction: Off

Monoisotopic Mass, Odd and Even Electron Ions
306 formula(e) evaluated with 3 results within limits (all results (up to 1000) for each mass)
Elements Used:
C: 0-100 H: 0-100 N: 0-8 O: 0-5

DCI-CH4 GCT Premier CAB109 07-Jul-202211:16:41
20220707-CL9-51 27 (0.450) Cm (27:32-2:6x5.000) TOF MS CI+
5.11e+003

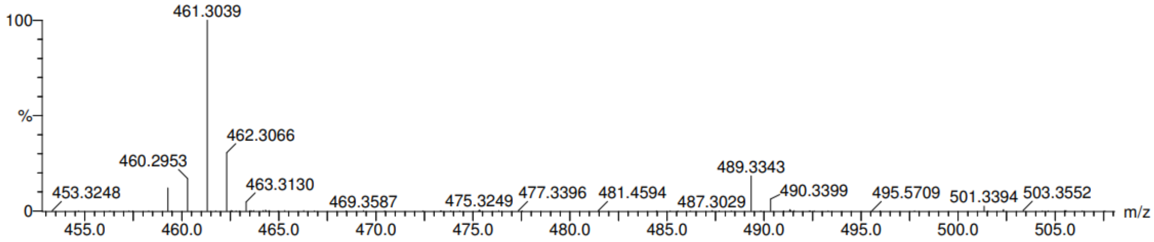

Minimum:				-1.5		
Maximum:		1.5	5.0	80.0		
Mass	Calc. Mass	mDa	PPM	DBE	i-FIT	Formula
461.3039	461.3042	-0.3	-0.7	12.0	18.1	C29 H39 N3 O2
	461.3029	1.0	2.2	12.5	7.8	C27 H37 N6 O
	461.3056	-1.7	-3.7	11.5	33.0	C31 H41 O3

Selected Crystallographic Data

Crystallographic data for compounds **4g** and **4h** were collected at 193(2) K using Mo K_α radiation (λ=0.71073 Å) on a Bruker-AXS Kappa APEX II Quazar diffractometer equipped with a 30W air-cooled microfocus source (**4h**) and on a Bruker-AXS D8-Venture diffractometer equipped with sealed tube, a multilayer TRIUMPH X-Ray mirror and a CMOS detector (**4g**). Phi- and omega-scans were used. Space group was determined on the basis of systematic absences and intensity statistics. Semi-empirical absorption correction was employed [1]. The structures were solved using an intrinsic phasing method (SHELXT), [2] and refined using the least-squares method on F^2 [3]. All non-H atoms were refined with anisotropic displacement parameters. Hydrogen atoms were refined isotropically at calculated positions using a riding model with their isotropic displacement parameters constrained to be equal to 1.2 times the equivalent isotropic displacement parameters of their pivot atoms. In both structures, H on Nitrogen atom was located from difference Fourier maps and freely refined.

CCDC-2358967 (**4g**) and CCDC-2358968 (**4h**) contain the supplementary crystallographic data for this paper. These data can be obtained free of charge from The Cambridge Crystallographic Data Centre via https://www.ccdc.cam.ac.uk/structures/.

Selected data for 4g : $C_{20}H_{23}N_3O$ M = 321.41, monoclinic, space group *Cc*, a = 16.5493(8) Å, b = 9.3643(5) Å, c = 12.5940(11) Å, β = 121.4202(14)°, V = 1665.54(19) Å^3, Z = 4, crystal size 0.30 x 0.30 x 0.20 mm^3, 29335 reflections collected (4037 independent, R_{int} = 0.0472), 221 parameters, 2 restraints, $R1$ [I>2σ(I)] = 0.0394, $wR2$ [all data] = 0.0845, largest diff. peak and hole: 0.183 and –0.238 eÅ^{-3}.

Selected data for 4h : $C_{22}H_{23}N_3O$ M = 345.43, monoclinic, space group $P2_1/c$, a = 7.7403(5) Å, b = 6.9103(4) Å, c = 32.243(2) Å, β = 96.004(4)°, V = 1715.14(18) Å^3, Z = 4, crystal size 0.20 x 0.06 x 0.04 mm^3, 23491 reflections collected (4323 independent, R_{int} = 0.0525), 239 parameters, $R1$ [I>2σ(I)] = 0.0468, $wR2$ [all data] = 0.1291, largest diff. peak and hole: 0.419 and –0.211 eÅ^{-3}.

References

1. Bruker. *SADABS*; Bruker AXS Inc.: Madison, WI, USA, 2008.
2. Sheldrick, G.M *ShelXT, Acta Crystallogr. Sect. A*; University of Göttingen: Göttingen, Germany, 2015.
3. Sheldrick, G.M *ShelXT, Acta Crystallogr. Sect. C*; University of Göttingen: Göttingen, Germany, 2015.
